# Effects of Heavy Metals and Arbuscular Mycorrhiza on the Leaf Proteome of a Selected Poplar Clone: A Time Course Analysis

**DOI:** 10.1371/journal.pone.0038662

**Published:** 2012-06-26

**Authors:** Guido Lingua, Elisa Bona, Valeria Todeschini, Chiara Cattaneo, Francesco Marsano, Graziella Berta, Maria Cavaletto

**Affiliations:** Dipartimento di Scienze e Innovazione Tecnologica, University of Piemonte Orientale “A. Avogadro”, Alessandria, Italy; Lawrence Berkeley National Laboratory, United States of America

## Abstract

Arbuscular mycorrhizal (AM) fungi establish a mutualistic symbiosis with the roots of most plant species. While receiving photosynthates, they improve the mineral nutrition of the plant and can also increase its tolerance towards some pollutants, like heavy metals. Although the fungal symbionts exclusively colonize the plant roots, some plant responses can be systemic. Therefore, in this work a clone of *Populus alba* L., previously selected for its tolerance to copper and zinc, was used to investigate the effects of the symbiosis with the AM fungus *Glomus intraradices* on the leaf protein expression. Poplar leaf samples were collected from plants maintained in a glasshouse on polluted (copper and zinc contaminated) or unpolluted soil, after four, six and sixteen months of growth. For each harvest, about 450 proteins were reproducibly separated on 2DE maps. At the first harvest the most relevant effect on protein modulation was exerted by the AM fungi, at the second one by the metals, and at the last one by both treatments. This work demonstrates how importantly the time of sampling affects the proteome responses in perennial plants. In addition, it underlines the ability of a proteomic approach, targeted on protein identification, to depict changes in a specific pattern of protein expression, while being still far from elucidating the biological function of each protein.

## Introduction

Heavy metal (HM) contamination of soils represents a serious concern for its possible consequences on the environment and human health [1]. Actually, the list of the 10 most polluted sites in the world includes 6 cases of HM excess, due to chromium, lead, mercury and various metal mixes, with millions of people potentially exposed to acute or chronic intoxication. As HMs cannot be degraded by biological or chemical processes, and thus tend to accumulate in soils and aquatic sediments, methods for the restoration of soils must be set up.

Phytoremediation, the plant-mediated reclamation of polluted soils, is receiving increasing attention because of its lower costs in comparison to more traditional approaches, its consensus in public opinion, and the possibility to restore the biological features of the soil and especially the microbial soil community [2], [3]. Early phytoremediation studies mainly focused on heavy metal hyperaccumulating plants. However, these are mostly herbaceous annuals of small size, therefore with severe limitations concerning the amount of extractable metals in a reasonable time period [4]. More recently, trees and woody perennials, and especially those of large size and fast growth, like poplars, have gained much interest. This attention is due to the large amount of metals they can accumulate in spite of the relatively low metal concentrations in their tissues [5], [6].

In order to improve the efficiency of the reclamation process, by increasing the uptake, translocation, accumulation and tolerance of heavy metals by the plant, various aspects of plant biology and ecology are under exploration, even in poplar species. These include the selection for tolerant varieties and useful plant traits [7]–[9], the investigation of the gene and protein expression of plants grown on polluted substrates [10]–[17], the introduction in the plant genome of genes increasing tolerance to HM-stress [18], [19], the study of some biochemical mechanisms known to be involved in defense or stress response [9], [13], the examination of the interactions between plants and soil microorganisms [11], [20]–[24]. Soil microorganisms are known to increase plant tolerance to stress [21] and can themselves be involved in soil restoration in a process taking the name of “bio-augmentation” [25]. In this respect, arbuscular mycorrhizal fungi (AMF) are especially important because they colonize most land plants in a huge variety of climatic conditions, improve plant nutrition and stress tolerance, and have also been shown to be useful for the revegetation of poor, marginal or polluted soils [26]–[29].

Although colonization by AMF is restricted to the root system, its effects are often detectable, even macroscopically, in the above-ground portion of plants [26]. Furthermore, leaves are responsible for carbon uptake and transpiration, and they can be the site of accumulation of some heavy metals [30]–[33]. Therefore, in order to better understand the mechanisms of tolerance, detoxification and stress response, the study of leaves of plants grown under HM stress is extremely relevant, both for basic knowledge and for application in phytoremediation approaches (especially for phytoextraction).

The responses of the poplar leaf proteome have been studied in a number of cases, including the exposition to cadmium [14]–[16], ozone [34], drought [35], [36] or heat stress [37], but not in the presence of AMF. In the context of phytoremediation, the effects of AMF on the plant stress response have been studied with a proteomic approach in the fronds and roots of the fern *Pteris vittata* grown under high arsenic concentrations [38], [39]. To our knowledge, there are no studies on the effects of the AM symbiosis on the leaf proteome of poplar plants grown on HM polluted soil.

In an effort to acquire further knowledge on metal detoxification and tolerance in a tree species, and in the context of a broader project on the use of poplar in phytoremediation, here we report a proteomic study concerning the leaves of a poplar clone selected for its metal tolerance, inoculated or not with the arbuscular mycorrhizal fungus *Glomus intraradices*, and grown on a soil with high copper and zinc concentrations. The final expected outcome of these studies should be an optimized system for phytoremediation, consisting of a selected poplar clone and a fungal symbiont with known molecular processes.

In the present case, attention was focused on the leaves of poplar because of the role of this organ in carbon fixation and because zinc is especially accumulated in its tissues. Furthermore, the analyses concerned three time points (4, 6 and 16 months after the establishing of the cultures, sampling S1, S2 and S3, respectively), allowing the consideration of time effects and long term adaptations to the heavy metal stress. This is the first time that plant proteome responses have been followed for such a long time lapse, revealing that changes in the protein expression patterns were strongly connected to the time of sampling.

## Results

### Poplars Biomass Production and Mycorrhizal Colonization

At sampling S3, plants grown on polluted soil showed the lowest values of biomass (Table 1). In plants inoculated with the AM fungus and grown on polluted soil (GiPoll), growth was restored to levels comparable to those of controls, with the exception of leaf biomass (Table 1).

**Table 1:** Root, stem and leaf dry weight (g) of poplar clone AL 35 at the final harvest (S3).

	C	Poll	Gi	GiPoll
**Root**	2.545±0.964 a	0.787±0.072 b	3.293±0.153 a	3.403±0.800 a
**Stem**	5.855±1.689 a	1.310±0.384 b	7.197±0.090 a	8.310±0.485 a
**Leaves**	2.523±0.858 a	0.503±0.072 b	2.170±0.214 a	0.433±0.038 b

C: plant grown on control (un-polluted) soil; Gi: plant grown on control soil and inoculated with *G. intraradices*; Poll: plant grown on polluted soil; GiPoll: plant grown on polluted soil and inoculated with *G. intraradices*. Different letters indicated significant differences (p<0.05) among the rows.

Metal presence did not affect mycorrhizal colonization (M%): at the end of the experiment M% was around 20% in the root system of plants inoculated with *G. intraradices* and grown on either polluted or non-polluted soil (Gi), as previously reported in a paper describing the variations of gene expression in the same individual plants [17].

### Metals and Phosphorus Concentration in Plant Organs

#### Copper

In leaves, and especially in those of plants grown on polluted soil, Cu accumulation increased with time, ranging between 10.86 (sampling S1) and 26.90 (sampling S3) mg/Kg dry weight (d. wt) (Table 2). Cu was mostly accumulated in roots, with the highest levels recorded in GiPoll plants (244.69 mg/Kg d. wt), a value significantly higher than those of the other treatments (Table 3).

**Table 2:** Metal and phosphorus concentration in poplar leaves.

	Leaves S1		
treatment	Cu	Zn	P
C	13.43±1.12 a	184.20±61.41 a	879.18±79.06 a
Poll	13.57±1.18 a	235.83±52.17 ab	825.77±74.34 a
Gi	10.86±0.86 a	197.62±17.67 a	805.71±72.46 a
GiPoll	13.10±1.21 a	284.10±25.44 b	734.53±66.07 a
	**Leaves S2**		
**treatment**	**Cu**	**Zn**	**P**
C	17.76±1.62 a	313.36±28.18 a	1796.82±161.68 a
Poll	17.88±1.64 a	442.10±39.81 b	1194.96±107.67 a
Gi	15.81±1.38 a	384.02±31.38 b	1323.26±118.89 a
GiPoll	13.99±1.23 a	522.07±47.08 c	1518.95±136.66 a
	**Leaves S3**		
**treatment**	**Cu**	**Zn**	**P**
C	13.76±1.31 a	286.50±60.87 a	1564.47±140.77 a
Poll	20.16±1.79 b	387.12±34.95 a	1535.03±137.99 a
Gi	13.01±1.23 a	284.97±26.01 a	1834.88±165.14 ab
GiPoll	26.90±2.38 c	461.18±41.73 b	2687.07±241.87 b

Data are mean and standard error of Cu, Zn and P concentration (mg/Kg d. wt) in leaves of *P. alba* plants at first (S1), second (S2) and third (S3) sampling. C – un-inoculated plants grown on a control soil; Gi – plants inoculated with *G. intraradices*, grown on control soil; Poll – plants grown on polluted soil; GiPoll – plants grown on polluted soil and inoculated with *G. intraradices*. Different letters in each column represented significant differences (p<0.05).

**Table 3:** Metal and phosphorus concentrations in stem, root and soil at S3 sampling.

	Stem		
treatment	Cu	Zn	P
C	8.45±0.69 a	82.09±7.28 a	1225.50±110.21 a
Poll	19.07±1.74 b	126.96±11.28 b	768.45±69.24 b
Gi	5.73±0.49 a	76.19±6.93 a	739.62±66.98 b
GiPoll	5.66±0.53 a	116.40±10.53 b	505.37±45.39 c
	**Root**		
	**Cu**	**Zn**	**P**
C	37.13±8.28 a	92.24±8.21 a	1908.38±171.82 a
Poll	97.56±8.65 b	98.50±8.89 a	1001.17±90.01 b
Gi	15.72±5.40 a	37.87±3.37 b	1321.19±118.98 bc
GiPoll	244.69±21.88 c	115.76±10.39 a	1726.62±155.30 c
	**Soil**		
	**Cu**	**Zn**	**P**
C	80.77±8.69 a	242.45±8.60 a	879.18±9.28 a
Poll	2396.40±8.79 b	2289.12±9.05 b	825.77±8.95 a
Gi	71.72±9.63 a	193.05±9.04 a	805.71±9.11 a
GiPoll	1083.61±8.44 c	1091.78±8.85 c	734.53±8.95 b

Table 3: Data are the means, with standard errors, of Cu, Zn and P concentration (mg/Kg d. wt) in stem, root and soil (total metals) of *P. alba* plants at harvest, third (S3) sampling. C – un-inoculated plants grown on a control soil; Gi – plants inoculated with *G. intraradices*, grown on control soil; Poll – plants grown on polluted soil; GiPoll – plants grown on polluted soil and inoculated with *G. intraradices*. Different letters in each column indicate significant differences (p<0.05).

#### Zinc

In general, Zn accumulation mainly occurred in leaves, with concentrations about one order of magnitude higher than those observed for Cu (Table 2). In this organ, Zn concentration significantly increased from the first to the second sampling. At the third sampling, the metal concentration was higher than that measured one year before in the same period (July), but lower than that recorded at the end of the first growing season. Plants grown on polluted soil (and especially GiPoll ones) always showed the highest Zn concentration in leaves (Table 2).

At the end of the experiment, Zn accumulation in the stems was lower than in the leaves, with significant differences between plants grown on control (82.09 mg/Kg d. wt) or polluted soil (126.96 and 116.40 mg/Kg d. wt, in Poll and GiPoll plants respectively) (Table 3).

**Table 4:** List of poplar leaf proteins from the first sampling, identified by MS/MS analysis, including average ratio of protein abundance.

Spot (Cor.)^a)^	Pep.^b)^	Seq. Cov.	Protein (BLAST results)	M_r_ (kDa)/pI Theor	M_r_ (kDa)/pI Exp	AC number (gi NCBI) and reference organism
**104_I**	2	6%	RuBisCO large subunit	52.9/6.14	70.0/5.68	gi|2961315 *Spigelia anthelmia*
**112_I**	2	6%	Heat shock protein 70	71.4/5.07	71.1/5.13	gi|6911551 *Cucumis sativus*
**124_I**	6	17%	ATP synthase beta subunit	51.8/5.20	71.0/5.20	gi|14718046 *Eucryphia lucida*
**130_I**	4	12%	Predicted protein (Enolase)	47.9/5.67	50.3/5.70	gi|224136806 *Populus trichocarpa*
**153_I**	15	51%	ATP synthase beta subunit	53.6/5.09	62.6/4.92	gi|110227086 *Populus alba*
**154_I**	28	73%	ATP synthase beta subunit	53.6/5.09	62.6/5.15	gi|110227086 *Populus alba*
**165_I**	4	6%	RuBisCO large subunit	49.6/6.60	49.6/5.80	gi|46326306 *Salvia chamaedryoides*
**230_I**	1	2%	Putative clathrin binding protein (epsin)	30.8/9.30	45.9/5.64	gi|3763925 *Arabidopsis thaliana*
**247_I (174_II) (613_III)**	5	21%	Unknown (Fructose bisphosphate aldolase)	42.9/8.17	43.5/6.24	gi|118489355 *Populus trichocarpa x Populus deltoides*
**283_I**	3	21%	Unknown (Thiamine biosynthetic enzyme)	29.3/5.26	38.7/5.74	gi|118488026 *Populus trichocarpa*
**304_I**	8	42%	Predicted protein	29.1/5.69	36.3/6.12	gi|224072767 *Populus trichocarpa*
**314_I (245_II) (301_III)**	2	11%	Predicted protein (NAD-dependent epimerase/dehydratase	27.0/5.68	38.8/5.30	gi|224090705 *Populus trichocarpa*
**397_I**	6	10%	RuBisCO large subunit	52.9/5.88	23.5/5.40	gi|1346967 *Brassica oleracea*
**470_I**	2	17%	Heat shock protein 17.0	17.0/5.78	17.0/6.47	gi|1122315 *Pennisetum glaucum*
**471_I**	7	24%	Isomerase peptidyl-prolyl cis-trans isomerase	28.2/9.40	17.0/6.48	gi|224057792 *Populus trichocarpa*
**484_I**	2	4%	BiP isoform B	73.4/5.11	73.4/5.11	gi|475600 *Glycine max*
**485_I**	4	8%	Unknown (Hsp70)	71.1/5.10	71.1/5.10	gi|219885633 *Zea mays*
**491_I**	2	1%	Hypothetical protein SORBIDRAFT_03g039980 (Laccase-8)	60.2/6.49	43.9/4.89	gi|242054991 *Sorghum bicolor*
**494_I**	4	11%	Predicted protein (Elongation factor Tu)	52.7/6.00	53.7/5.37	gi|224053971 *Populus trichocarpa*

a) In brackets, corresponding spot number in the other samplings (manually checked and confirmed by MS/MS analysis).

b) Number of identified peptides.

Graphical representation of the average ratios of the protein abundance is shown in Table S1 of the supplementary materials.

Root Zn concentration was lowest in Gi plants (37.87 mg/Kg d. wt.), if compared to the other treatments (Table 3).

#### Phosphorus

Phosphorus concentration in leaves increased from sampling S1 to S3 (Table 2). The four treatments did not show significant differences for the first two samplings. At sampling S3, plants inoculated with *G. intraradices* showed a higher P concentration than their uninoculated counterparts, and GiPoll plants presented the highest P accumulation (2687.07 mg/Kg d. wt).

Stem P concentration ranged between 505.37 and 1225.50 mg/Kg d. wt in GiPoll and control (C) plants respectively (Table 3). No significant differences were recorded between Gi and Poll plants.

In roots, phosphorus concentration was highest in control plants, with significant differences in comparison to the other treatments, while the lowest value was recorded in Poll plants. No differences were detected between Gi plants and those grown on polluted soil, inoculated or not.

### Leaf Proteome Response

**Figure 1:**
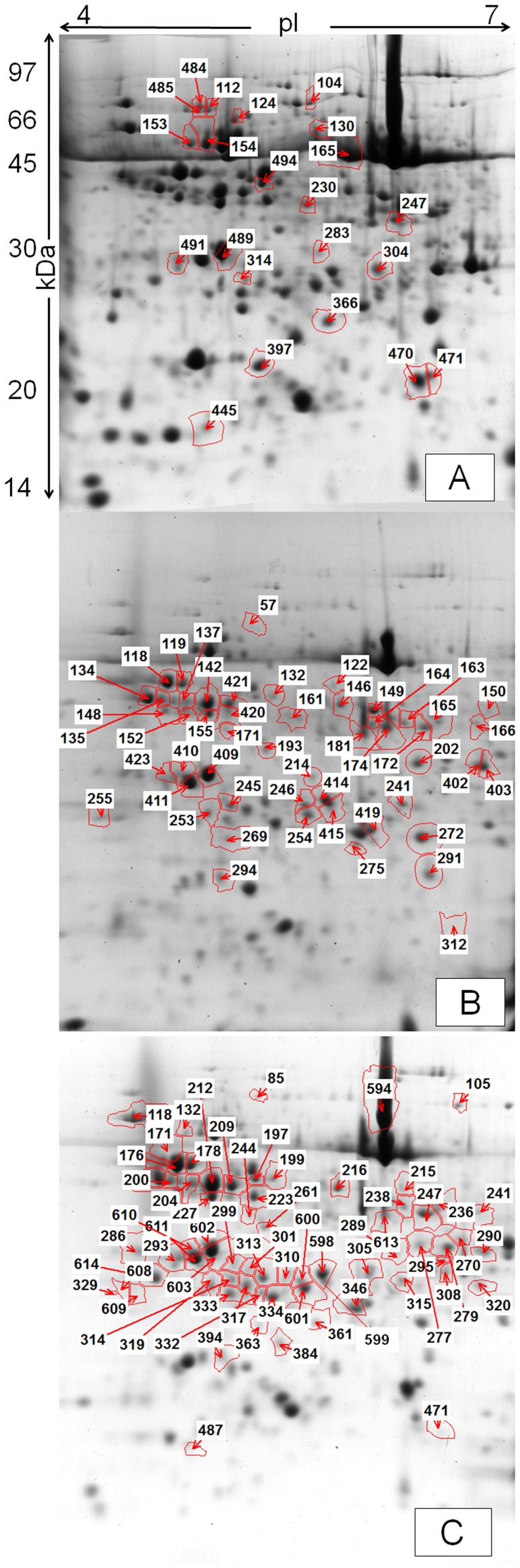
Two-dimensional maps of poplar leaf proteins. Representative 2-DE maps of poplar leaf proteins (500 µg) stained with Blue silver, colloidal Coomassie, (**a**) sampling S1, (**b**) sampling S2, and (**c**) sampling S3. IEF was performed with 13 cm IPG strips pH 4–7, followed by SDS-PAGE on 12% gel. Differently expressed spots are highlighted.

The 2D maps of leaf proteins, stained with Colloidal Coomassie, showed a mean of 450 spots reproducibly separated for each of the three samplings (Figures 1A–C and Figure S1 of supplementary materials). Statistically significant variations were detected for 22 spots (of which 19 were identified) at sampling S1, 52 spots (47 identified) at sampling S2, 66 spots (59 identified) at sampling S3.

Tables 4, 5, 6 list the number of identified peptides, sequence coverage, BLAST results, theoretical and experimental molecular weight and pI accession number and reference organism of each identified protein for the three samplings (the graph of the relative expression level is available in the supplementary materials, Tables S1, S2, S3). Moreover the corresponding spot, possibly identified in other samplings, is indicated. In the supplementary materials, Tables S4, S5, S6 list the raw data of optical densities and the respective ANOVA P-values; Tables S7, S8, S9 list the MS/MS data (precursor ions, peptide sequence, ion score, modifications, protein name, entries and BLAST results); Tables S10, S11, S12 report BLAST result details.

**Table 5:** List of poplar leaf proteins from the second sampling, identified by MS/MS analysis, including average ratio of protein abundance.

Spot (Cor.)^a)^	Pep.^b)^	Seq. Cov.	Protein(BLAST result)	M_r_ (kDa)/pI Theor	M_r_ (kDa)/pI Exp	AC number (gi NCBI) and reference organism
**118_II (176_III)**	14	43%	Unknown (RuBisCO activase)	52.0/6.28	51.5/4.90	gi|118487547 *Populus trichocarpa*
**119_II (178_III)**	12	38%	Unknown (RuBisCO activase)	52.0/6.28	51.5/5.00	gi|118487547 *Populus trichocarpa*
**122_II**	4	13%	Predicted protein (Phosphoglycerate kinase)	50.2/8.25	51.5/5.90	gi|224109060 *Populus trichocarpa*
**132_II**	1	3%	Elongation factor Tu	52.1/6.21	50.0/5.50	gi|2494261 *Glycine max*
**134_II (199_III)**	10	31%	Unknown (RuBisCO activase)	50.6/8.36	51.9/4.90	gi|118489408 *Populus trichocarpa x Populus deltoides*
**135_II (200_III)**	9	23%	Unknown (RuBisCO activase)	51.9/5.26	51.9/4.90	gi|118486739 *Populus trichocarpa*
**137_II**	10	25%	Unknown (RuBisCO activase)	52.1/6.28	51.9/5.00	gi|118487547 *Populus trichocarpa*
**142_II (212_III)**	11	18%	Unnamed protein product (RuBisCO activase)	51.9/5.15	51.9/5.15	gi|157345989 *Vitis vinifera*
**146_II (216_III)**	12	36%	Predicted protein (Phosphoglycerate kinase)	50.2/8.25	48.6/5.90	gi|224109060 *Populus trichocarpa*
**148_II**	1	1%	Putative plastid isopentenyl diphosphate/dimethylallyl diphosphate synthase precursor	49.9/5.38	51.8/4.90	gi|209402463 *Mantoniella squamata*
**149_II**	5	27%	Predicted protein (Glutamine synthetase)	39.2/5.52	47.7/6.15	gi|224079530 *Populus trichocarpa*
**150_II**	3	10%	Predicted protein (Uroporphyrinogen decarboxylase)	44.5/7.14	47.7/6.85	gi|224145917 *Populus trichocarpa*
**152_II**	8	30%	Predicted protein (Phosphoribulokinase)	45.0/5.90	51.8/5.00	gi|224071429 *Populus trichocarpa*
**155_II (227_III)**	11	40%	Predicted protein (Phosphoribulokinase)	45.0/5.90	51.8/5.15	gi|224071429 *Populus trichocarpa*
**161_II**	3	13%	Unknown (Protein disulfide isomerase, putative)	34.9/5.31	46.5/5.70	gi|118482960 *Populus trichocarpa*
**162_II**	11	46%	Predicted protein (Malate dehydrogenase)	35.7/6.11	45.0/6.25	gi|224102193 *Populus trichocarpa*
**163_II**	3	14%	Cytosolic malate dehydrogenase	35.5/5.92	44.9/6.30	gi|10334493 *Cicer arietinum*
**164_II**	7	22%	Predicted protein (Aldo/keto reductase AKR)	37.4/5.97	45.7/6.14	gi|224069096 *Populus trichocarpa*
**165_II**	2	7%	Cytosolic malate dehydrogenase	35.5/5.92	44.9/6.53	gi|10334493 *Cicer arietinum*
**166_II**	3	12%	Cytosolic malate dehydrogenase	35.5/5.92	44.9/6.80	gi|10334493 *Cicer arietinum*
**171_II**	2	15%	RuBisCO activase	25.9/5.01	45.6/5.28	gi|100380 *Nicotiana tabacum*
**172_II**	2	6%	Hypothetical protein	20.1/5.54	44.9/6.40	gi|147835353 *Vitis vinifera*
**174_II (247_I) (613_III)**	3	14%	Unknown (Fructose-bisphosphate aldolase)	42.9/8.17	44.9/6.21	gi|118489355 *Populus trichocarpa x Populus deltoides*
**181_II**	3	15%	Unknown (Fructose-bisphosphate aldolase)	42.8/7.55	44.0/6.08	gi|118487575 *Populus trichocarpa*
**193_II**	2	8%	GGDP synthase	39.2/5.38	41.4/5.52	gi|9971808 *Tagetes erecta*
**202_II**	3	9%	Ferredoxin-NADP+ reductase	40.1/8.66	40.0/6.40	gi|5730139 *Arabidopsis thaliana*
**245_II (314_I) (301_III)**	4	25%	Predicted protein (NAD-dependent epimerase/dehydratase)	27.0/5.68	33.9/5.34	gi|224090705 *Populus trichocarpa*
**246_II**	7	41%	Predicted protein (Ascorbate peroxidase)	27.3/5.53	34.1/5.70	gi|224104631 *Populus trichocarpa*
**253_II (319_III)**	3	18%	Predicted protein (Groes chaperonin)	27.1/7.77	32.0/5.22	gi|224141565 *Populus trichocarpa*
**254_II**	5	39%	Putative ascorbate peroxidase	22.4/4.83	32.0/5.70	gi|46911557 *Populus x Canadensis*
**255_II**	6	42%	Predicted protein (Ribose-5-phosphate isomerase, putative)	30.9/5.36	32.0/4.60	gi|224130670 *Populus trichocarpa*
**269_II**	8	20%	Predicted protein (Tau class glutathione transferase)	25.4/5.31	29.7/5.33	gi|224117556 *Populus trichocarpa*
**272_II**	3	22%	Hypothetical protein POPTRDRAFT_551203 (Photosystem II reaction center psbP Protein)	28.2/7.68	29.7/6.53	gi|224062595 *Populus trichocarpa*
**275_II**	3	9%	Predicted protein (ATP-dependent Clp protease)	32.7/6.79	27.0/6.00	gi|224068558 *Populus trichocarpa*
**291_II**	7	33%	Hypothetical protein POPTRDRAFT_818640 (Probable oxygen-evolving enhancer protein 2)	28.1/8.65	24.3/6.54	gi|224085421 *Populus trichocarpa*
**294_II**	3	12%	RuBisCO activase precursor	40.8/7.59	23.5/5.33	gi|3687652 *Datisca glomerata*
**402_II**	2	10%	Esterase d, s-formylglutathione hydrolase	31.9/6.17	40.0/6.80	gi|224086942 *Populus trichocarpa*
**403_II**	8	28%	Predicted protein (Ferredoxin–NADP reductase)	40.4/8.71	40.2/6.85	gi|224074257 *Populus trichocarpa*
**409_II (603_III)**	2	27%	Putative protein (Oxygen-evolving enhancer protein 1)	18.5/5.17	38.3/5.10	gi|190898996 *Populus tremula*
**410_II**	4	18%	Photosystem II protein 33 kD	26.6/5.01	38.3/5.10	gi|224916 *Spinacia oleracea*
**411_II (610_III)**	11	37%	Unknown (Photosystem II oxygen-evolving complex 33)	35.1/5.62	35.1/5.17	gi|118489901 *Populus trichocarpa x Populus deltoides*
**414_II (598_III)**	3	14%	Ascorbate peroxidase	27.5/5.52	34.1/5.80	gi|42558486 *Rehmannia glutinosa*
**415_II**	4	14%	Predicted protein (Protein THYLAKOID FORMATION1)	33.6/7.59	33.9/5.80	gi|224146717 *Populus trichocarpa*
**419_II**	2	16%	Predicted protein (Manganese superoxide dismutase)	25.3/6.80	30.0/6.14	gi|224124440 *Populus trichocarpa*
**420_II (209_III)**	8	19%	Unknown (RuBisCO Activase)	52.0/6.28	48.3/5.31	gi|118489105 *Populus trichocarpa x Populus deltoides*
**421_II**	4	59%	Actin	17.2/4.73	48.8/5.31	gi|2887459 *Cucumis sativus*
**423_II**	3	27%	Putative protein (OEE protein 1)	18.5/5.17	38.3/5.00	gi|190898996 *Populus tremula*

a) In brackets, corresponding spot number in the other samplings (manually checked and confirmed by MS/MS analysis).

b) Number of identified peptides and sequence coverage.

Graphical representation of the average ratios of the protein abundance is shown in Table S2 of the supplementary materials.

**Table 6:** List of poplar leaf proteins from the third sampling, identified by MS/MS analysis, including average ratio of protein abundance.

Spot (Cor.)^a)^	Pep^b)^	Cov.	Protein (BLAST results)	M_r_ (kDa)/pI Theor	M_r_ (kDa)/pI Exp	AC number (gi NCBI) and reference organism
**85_III**	2	3%	Heat shock 70 kDa protein	70.8/5.37	70.1/5.5	gi|123601 *Glycine max*
**105_III**	2	4%	Predicted protein (heat shock protein 70 (HSP70)-interacting protein, putative)	65.5/6.17	70.1/6.60	gi|224071575 *Populus trichocarpa*
**118_III**	26	48%	Predicted protein (putative rubisco subunit binding-protein alpha subunit (Chaperonin))	62.0/5.24	62.0/5.24	gi|224104681 *Populus trichocarpa*
**132_III**	17	45%	ATP synthase beta subunit	52.0/5.05	52.0/5.05	gi|62085107 *Cespedesia bonplandii*
**171_III**	7	19%	Predicted protein (Phosphoribulose kinase, putative)	45.0/6.11	51.0/4.96	gi|224138316 *Populus trichocarpa*
**176_III (118_II)**	21	44%	Unknown (RuBisCO activase 1)	52.0/6.28	50.5/4.96	gi|118489105 *Populus trichocarpa x Populus deltoides*
**178_III (119_II)**	13	24%	RuBisCO activase	48.0/8.20	50.5/5.03	gi|3914605 *Malus x domestica*
**197_III**	6	20%	Predicted protein (EF-Tu protein)	46.6/5.60	49.5/5.50	gi|224074859 *Populus trichocarpa*
**199_III (134_II)**	18	37%	Unknown (RuBisCO activase (RCA))	50.7/8.36	49.5/4.94	gi|118489408 *Populus trichocarpa x Populus deltoides*
**200_III (135_II)**	5	13%	RuBisCO activase 2	48.3/5.06	49.5/4.96	gi|12620883 *Gossypium hirsutum*
**209_III (420_II)**	19	38%	Unknown (RuBisCO activase 1)	52.0/6.28	49.5/5.42	gi|118489105 *Populus trichocarpa x Populus deltoides*
**212_III (142_II)**	22	44%	Unknown (RuBisCO activase)	52.1/6.28	49.5/5.33	gi|118487547 *Populus trichocarpa*
**215_III**	7	16%	Predicted protein (Sedo-heptulose-1,7-bisphospha-tase, chloroplast, putative)	42.4/5.77	47.4/4.96	gi|224112589 *Populus trichocarpa*
**216_III (146_II)**	15	38%	Predicted protein (Phosphoglycerate kinase)	50.2/8.25	48.6/5.90	gi|224109060 *Populus trichocarpa*
**223_III**	5	17%	Predicted protein (Phosphoribulose kinase, putative)	45.0/6.11	47.7/5.50	gi|224138316 *Populus trichocarpa*
**227_III (155_II)**	13	38%	Predicted protein (Phosphoribulose kinase, putative)	45.0/5.90	47.0/5.40	gi|224071429 *Populus trichocarpa*
**236_III**	6	27%	Unknown (Alcohol dehydrogenase, putative)	40.6/8.49	45.0/6.50	gi|118488941 *Populus trichocarpa x Populus deltoides*
**238_III**	2	5%	Isovaleryl-CoA Dehydrogenase; auxin binding protein (ABP44)	44.5/6.27	45.0/6.33	gi|5869965 *Pisum sativum*
**241_III**	2	5%	Hypothetical protein (Aldo/keto reductase, putative)	40.5/6.69	45.0/6.70	gi|225446767 *Vitis vinifera*
**244_III**	3	11%	Predicted protein (Pyruvate dehydrogenase(acetyl-transferring))	38.6/5.87	44.0/5.47	gi|224053535 *Populus trichocarpa*
**247_III**	14	46%	Unknown (Alcohol dehydrogenase, putative)	40.6/8.49	44.9/6.40	gi|118488941 *Populus trichocarpa x Populus deltoides*
**261_III**	9	22%	Predicted protein	38.4/5.87	41.9/5.54	gi|224073126 *Populus trichocarpa*
**270_III**	3	6%	RuBisCO large subunit	52.0/6.10	38.1/6.60	gi|1293020 *Polyscias guilfoylei*
**277_III**	3	4%	RuBisCO large subunit	49.5/6.60	37.9/6.40	gi|46326306 *Salvia chamaedryoides*
**279_III**	2	5%	RuBisCO large subunit	48.6/6.80	37.9/6.50	gi|14585745 *Veronica arguta*
**286_III**	3	13%	Predicted protein	30.2/5.36	35.1/5.24	gi|224110036 *Populus trichocarpa*
**289_III**	2	10%	Chain A, Profilin I	14.1/4.70	37.9/6.31	gi|157836856 *Arabidopsis thaliana*
**290_III**	2	7%	Predicted protein (Ferredoxin–NADP reductase, putative)	40.4/8.71	37.9/6.70	gi|224074257 *Populus trichocarpa*
**293_III**	3	13%	Predicted protein (2-deoxyglucose-6-phosphate phosphatase, putative)	28.9/5.12	35.1/5.00	gi|224093744 *Populus trichocarpa*
**295_III**	4	12%	Predicted protein (Plastid-specific 30S ribosomal protein 1)	34.1/6.78	37.9/6.50	gi|224118512 *Populus trichocarpa*
**301_III (314_I) (245_II)**	3	23%	Predicted protein (NAD-dependent epimerase/dehydratase)	27.0/5.68	35.1/5.45	gi|224090705 *Populus trichocarpa*
**305_III**	4	17%	Unknown	33.4/6.97	34.8/6.10	gi|118484329 *Populus trichocarpa*
**308_III**	3	20%	Predicted protein (3-hydroxyisobutyrate dehydrogenase, putative)	30.6/6.45	34.8/6.50	gi|224129290 *Populus trichocarpa*
**310_III**	6	29%	Predicted protein (Cytosolic ascorbate peroxidase 1)	27.3/5.53	34.8/5.68	gi|224104631 *Populus trichocarpa*
**313_III**	11	56%	Predicted protein (NAD-dependent epimerase/dehydratase)	27.0/5.68	34.8/5.57	gi|224090705 *Populus trichocarpa*
**314_III**	8	39%	Predicted protein (NAD-dependent epimerase/dehydratase)	27.0/5.68	33.5/5.35	gi|224090705 *Populus trichocarpa*
**315_III**	2	10%	Unknown (ATP synthase subunit mitochondrial)	27.8/8.50	34.8/6.33	gi|118484162 *Populus trichocarpa*
**317_III**	4	21%	Predicted protein (Carboxy-methylenebutenolidase, putative)	26.2/5.24	32.0/5.45	gi|224131618 *Populus trichocarpa*
**319_III (253_II)**	5	18%	Predicted protein (Groes chaperonin, putative)	27.1/7.77	32.0/5.22	gi|224141565 *Populus trichocarpa*
**320_III**	2	10%	Predicted protein (Chloroplast drought-induced stress protein, putative)	26.3/5.94	34.8/6.70	gi|224085954 *Populus trichocarpa*
**332_III**	5	23%	Predicted protein (Chloroplast ferritin 2 precursor)	29.4/5.72	31.8/5.57	gi|224109256 *Populus trichocarpa*
**333_III**	5	34%	Predicted protein (Phi class glutathione transferase GSTF2)	24.6/5.52	31.8/5.35	gi|224065729 *Populus trichocarpa*
**334_III**	10	66%	Predicted protein (Glutathione-s-transferase theta)	24.6/5.52	31.8/5.60	gi|224065729 *Populus trichocarpa*
**346_III**	6	42%	Unknown (Light-harvesting complex I protein Lhca3)	29.6/9.10	29.6/6.00	gi|118489937 *Populus trichocarpa x Populus deltoides*
**361_III**	3	16%	Predicted protein (Heat shock protein, putative)	26.2/6.92	26.2/5.80	gi|224120952 *Populus trichocarpa*
**363_III**	2	9%	Predicted protein (Heat shock protein, putative)	26.2/6.92	26.0/5.57	gi|224120952 *Populus trichocarpa*
**384_III**	7	11%	RuBisCO	49.9/6.57	25.0/5.68	gi|6513629 *Ascarina* sp. Qiu-M149
**394_III**	4	7%	RuBisCO large subunit	51.1/6.33	23.5/5.35	gi|493246 *Disporum sessile*
**487_III**	8	39%	Predicted protein (Thylakoid lumenal 15 kDa protein, Chloroplast)	23.4/6.82	20.0/5.17	gi|224098455 *Populus trichocarpa*
**594_III**	32	50%	RuBisCO large subunit	52.7/5.91	62.0/6.29	gi|110227087 *Populus alba*
**598_III (414_II)**	11	53%	Predicted protein (Cytosolic ascorbate peroxidase 1)	27.3/5.53	34.8/5.80	gi|224104631 *Populus trichocarpa*
**600_III**	2	7%	Predicted protein	27.8/8.50	34.8/5.72	gi|224093896 *Populus trichocarpa*
**601_III**	12	41%	Unknown (Groes chaperonin, putative)	26.8/8.76	31.8/5.72	gi|118489858 *Populus trichocarpa x Populus deltoids*
**602_III**	5	14%	Unknown (2-deoxyglucose-6-phosphate phosphatase, putative)	35.2/8.00	37.9/5.33	gi|118488927 *Populus trichocarpa x Populus deltoides*
**603_III (409_II)**	8	38%	Unknown (Photosystem II oxygen-evolving complex 33 KDa subunit)	35.1/5.62	37.7/5.33	gi|118489901 *Populus trichocarpa x Populus deltoides*
**610_III (411_II)**	5	23%	Unknown (Photosystem II oxygen-evolving complex 33 KDa subunit)	35.1/5.62	35.1/5.17	gi|118489901 *Populus trichocarpa x Populus deltoides*
**611_II**	6	45%	Putative protein (Oxygen-evolving enhancer protein 1, chloroplast precursor, putative)	18.5/5.17	35.0/5.17	gi|190898996 *Populus tremula*
**613_III (247_I) (174_II)**	6	25%	Unknown (Fructose-bisphosphate aldolase, putative)	42.9/8.17	44.9/6.29	gi|118489355 *Populus trichocarpa x Populus deltoides*
**614_III**	10	47%	Predicted protein (DHAR class glutathione transferase DHAR1)	24.3/4.93	34.8/4.93	gi|224065178 *Populus trichocarpa*

a) In brackets, corresponding spot number in the other samplings (manually checked and confirmed by MS/MS analysis).

b) Number of identified peptides and sequence coverage.

Graphical representation of the average ratios of the protein abundance is shown in Table S3 of the supplementary materials.

**Figure 2:**
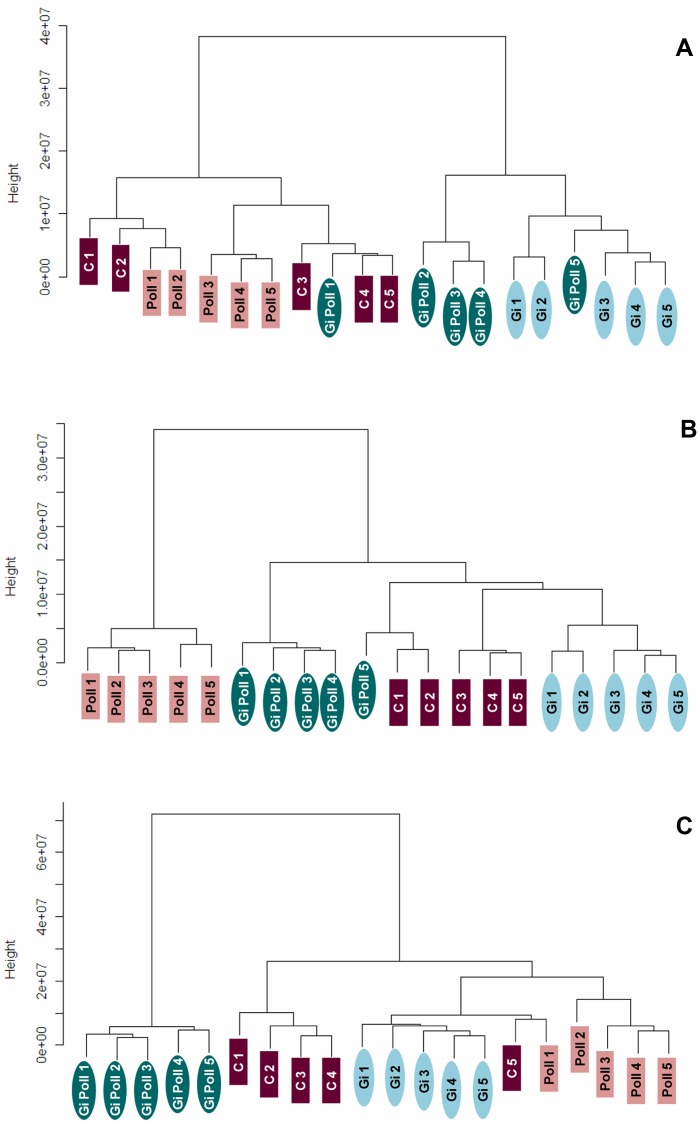
Cluster dendrograms. Cluster analysis performed using the optical densities of the differentially expressed spots for each replica using the software R (ver. 2.7.0); distances were calculated with the “Manhattan” method and a dendrogram was built with the “Ward” method. **(A)** sampling S1, **(B)** sampling S2, and **(C)** sampling S3. C – un-inoculated plants grown on a control soil; Gi – plants inoculated with *G. intraradices*, grown on control soil; Poll – plants grown on polluted soil; GiPoll – plants grown on polluted soil and inoculated with *G. intraradices*.

Cluster analysis of the optical density data from the 2D gels showed that the poplar leaf proteome changed with time as plants adapted to the metal stress and interacted with the root symbionts. Distinct clusters formed at each sampling date highlighting their differences (Figure 2). At sampling S1, two large clusters were formed, one of the mycorrhizal plants and the other of the non-inoculated poplars, regardless of the metal treatment (with the exception of replica 1 of the GiPoll plants) (Figure 2A). At sampling S2, when zinc concentrations were usually highest in the leaves, data from non-mycorrhizal plants grown on polluted soil clustered separately from the other treatments (Figure 2B). Finally, at sampling S3 (one year after S1), data from GiPoll plants clustered alone, showing a peculiar proteome profile induced by the simultaneous presence of both AM and HM (Figure 2C).

The two-way ANOVA (Tables S13, S14, S15 of the supplementary materials) indicated that at sampling S1, 100% of the varying proteins were affected by the factor “fungus”, 27% by the factor “metal” and 14% by the interaction of the two. At sampling S2 the situation was reversed, with 94% of the proteins significantly affected by the factor “metal”, 42% by the factor “fungus” and 29% by the interaction “fungus x metal”. At sampling S3 there was not a dominant factor, as 91% of the proteins showing significant variations were affected by the factor “fungus”, 92% by the factor “metal” and 42% by the interaction of the two.

**Figure 3:**
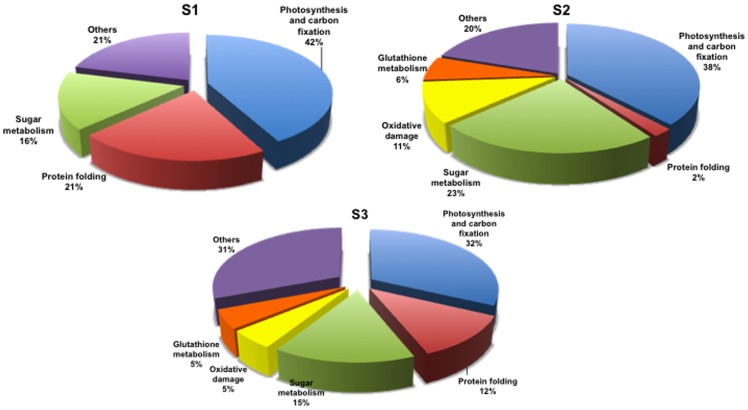
Proportion of identified proteins by functional categories. Pie charts showing percentages of the identified proteins belonging to different functional categories. S1: first sampling; S2: second sampling; S3: third sampling.

**Figure 4:**
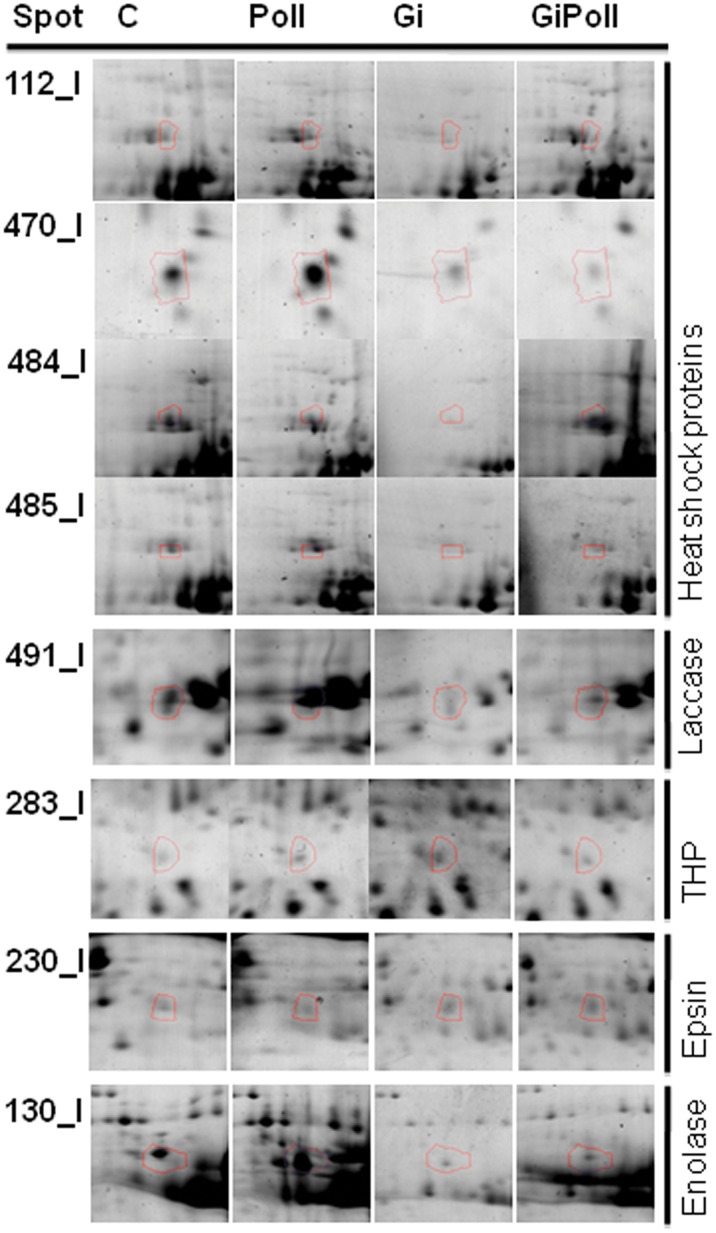
Enlarged details for some spots from S1 sampling. Details for the spots (112, 470, 484, 485, 491, 283, 230, 130) from C, Poll, Gi and GiPoll maps, including spot number and protein name. C – un-inoculated plants grown on a control soil; Gi – plants inoculated with *G. intraradices*, grown on control soil; Poll – plants grown on polluted soil; GiPoll – plants grown on polluted soil and inoculated with *G. intraradices*.

**Figure 5:**
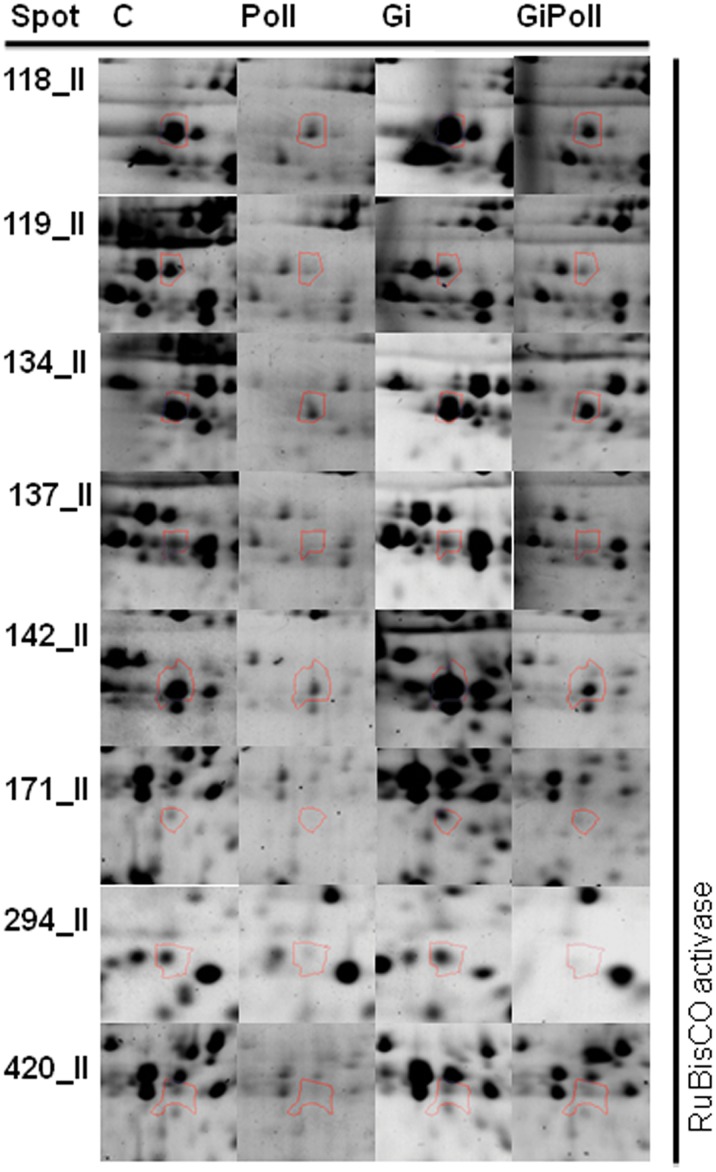
Enlarged details for some spots from S2 sampling. Details for the spots (118, 119, 134, 137, 142, 171, 294, 420) from C, Poll, Gi and GiPoll maps, including spot number and protein name. C – un-inoculated plants grown on a control soil; Gi – plants inoculated with *G. intraradices*, grown on control soil; Poll – plants grown on polluted soil; GiPoll – plants grown on polluted soil and inoculated with *G. intraradices*.

**Figure 6:**
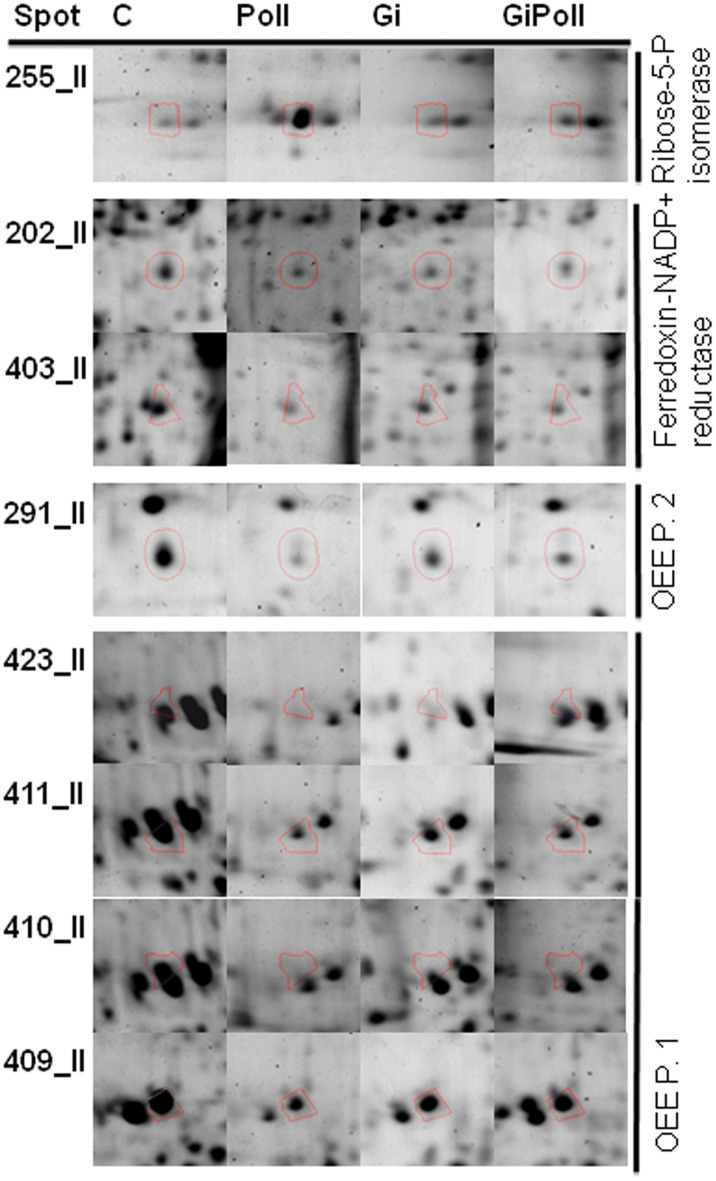
Enlarged details for some spots from S2 sampling. Details for the spots (255, 202, 403, 291, 423, 411, 410, 409) from C, Poll, Gi and GiPoll maps, including spot number and protein name. C – un-inoculated plants grown on a control soil; Gi – plants inoculated with *G. intraradices*, grown on control soil; Poll – plants grown on polluted soil; GiPoll – plants grown on polluted soil and inoculated with *G. intraradices*.

**Figure 7:**
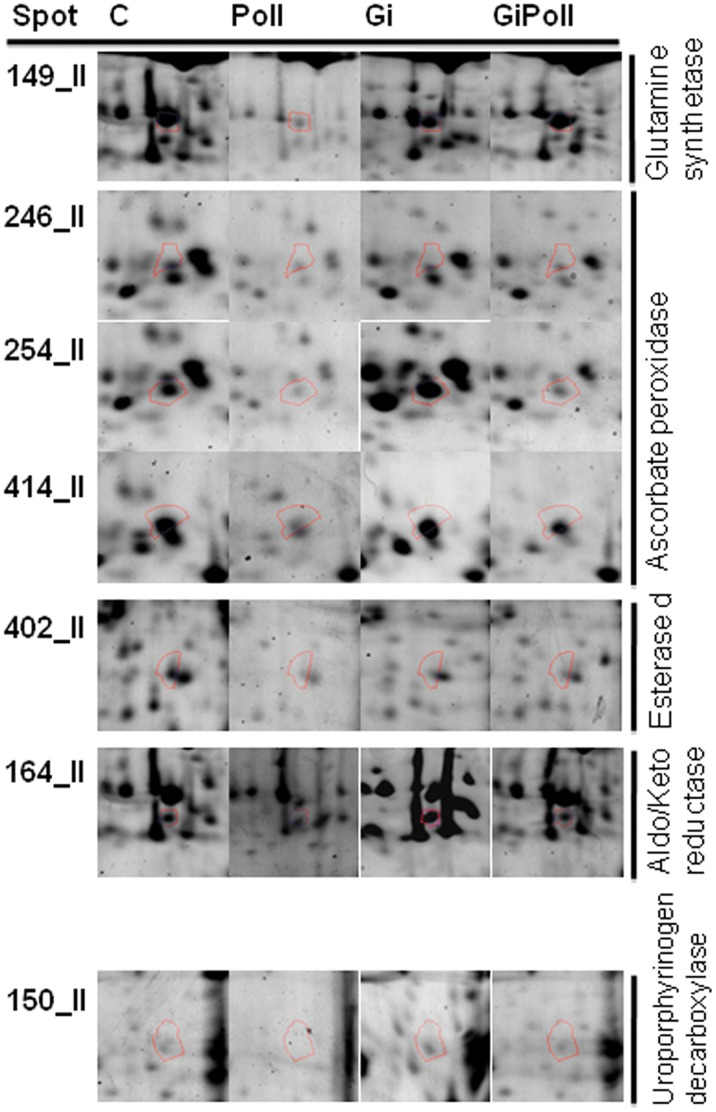
Enlarged details for some spots from S2 sampling. Details for the spots (149, 246, 254, 414, 402, 164, 150) from C, Poll, Gi and GiPoll maps, including spot number and protein name. C – un-inoculated plants grown on a control soil; Gi – plants inoculated with *G. intraradices*, grown on control soil; Poll – plants grown on polluted soil; GiPoll – plants grown on polluted soil and inoculated with *G. intraradices*.

**Figure 8:**
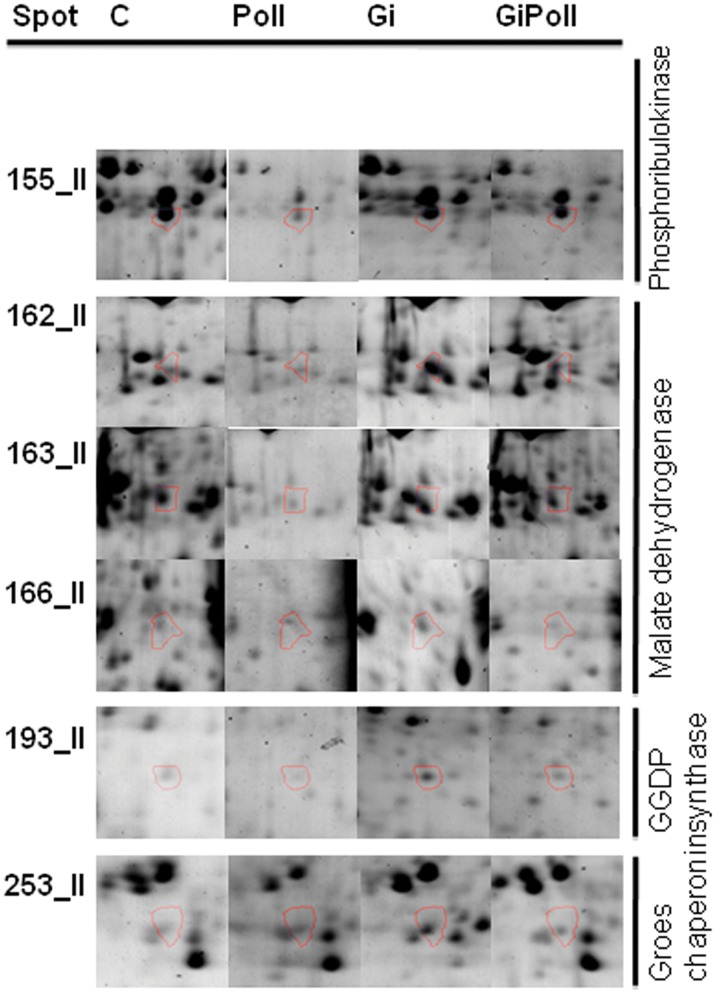
Enlarged details for some spots from S2 sampling. Details for the spots (155, 162, 163, 166, 193, 253) from C, Poll, Gi and GiPoll maps, including spot number and protein name. C – un-inoculated plants grown on a control soil; Gi – plants inoculated with *G. intraradices*, grown on control soil; Poll – plants grown on polluted soil; GiPoll – plants grown on polluted soil and inoculated with *G. intraradices*.

**Figure 9:**
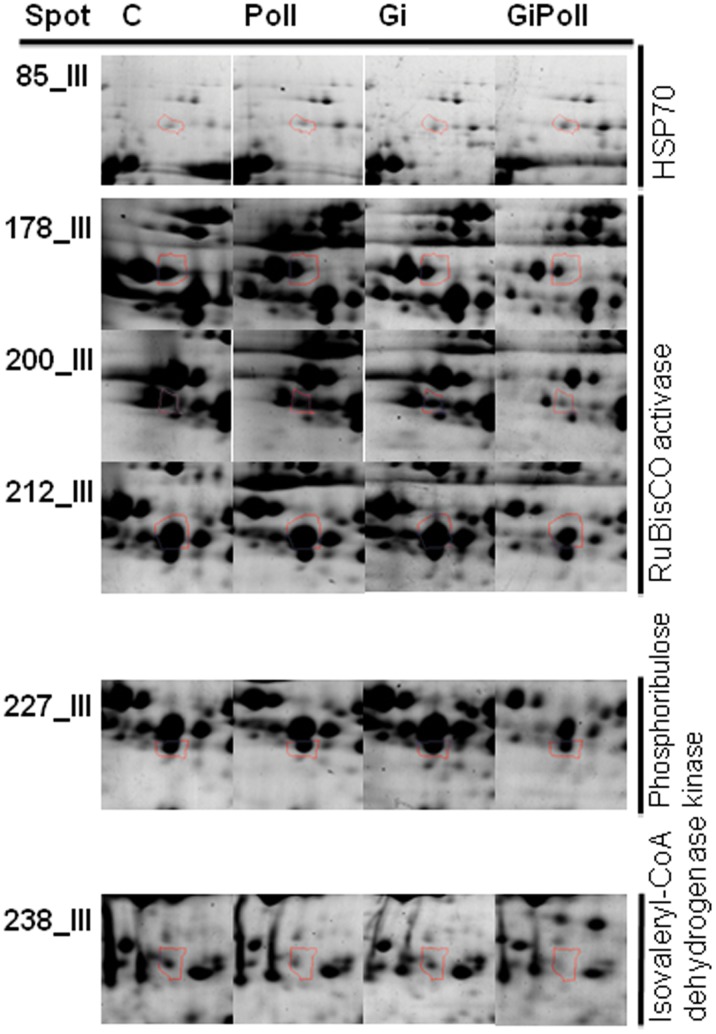
Enlarged details for some spots from S3 sampling. Details for the spots (85, 178, 200, 212, 227, 238) from C, Poll, Gi and GiPoll maps, including spot number and protein name. C – un-inoculated plants grown on a control soil; Gi – plants inoculated with *G. intraradices*, grown on control soil; Poll – plants grown on polluted soil; GiPoll – plants grown on polluted soil and inoculated with *G. intraradices*.

**Figure 10:**
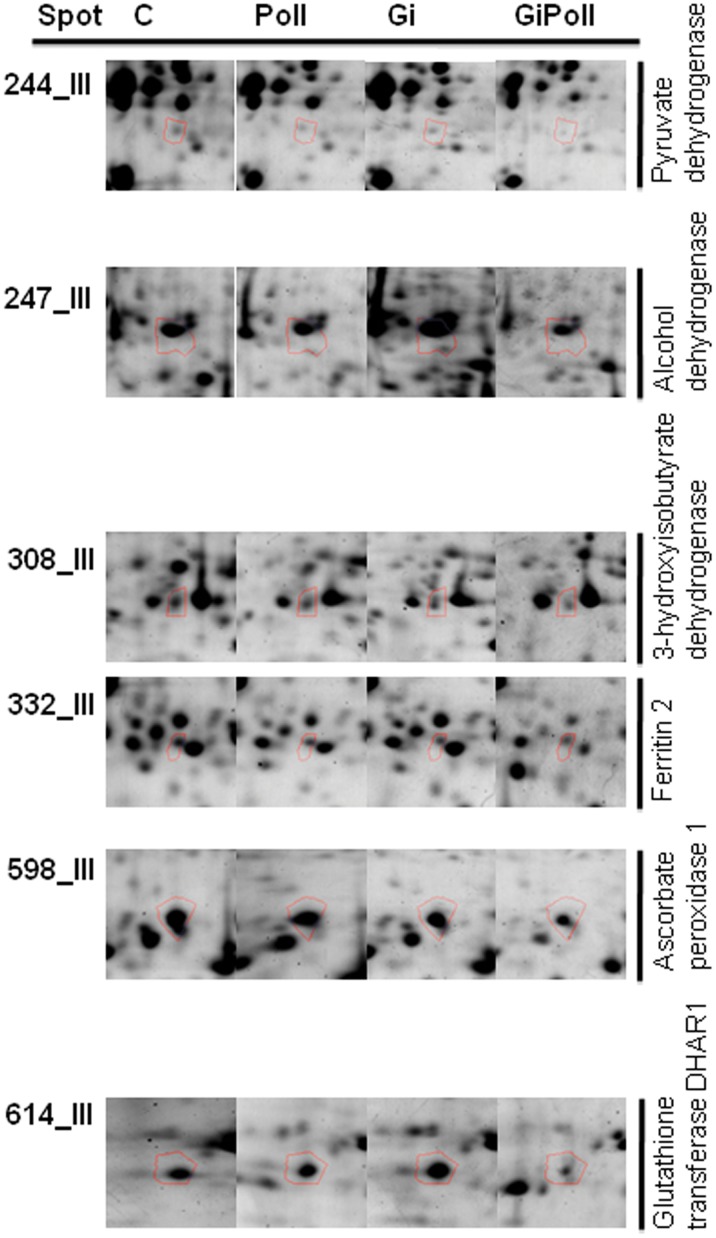
Enlarged details for some spots from S3 sampling. Details for the spots (244, 247, 308, 332, 598, 614) from C, Poll, Gi and GiPoll maps, including spot number and protein name. C – un-inoculated plants grown on a control soil; Gi – plants inoculated with *G. intraradices*, grown on control soil; Poll – plants grown on polluted soil; GiPoll – plants grown on polluted soil and inoculated with *G. intraradices*.

Figure 3 shows the percentage of identified proteins per sampling, according to their biological function. “Photosynthesis and carbon fixation” (32–42% of the total) and “Sugar metabolism” (15–23%) were largely represented at all samplings. “Protein folding” proteins were the second group by relevance at sampling S1 (21%), while their proportion dramatically decreased at samplings S2 (2%) and S3 (12%). The groups concerning “Glutathione metabolism” and “Oxidative damage” were not present at sampling S1 and appeared only from sampling S2 onwards. Figures 4, 5, 6, 7, 8, 9, 10 show magnified details of some identified spots from C, Poll, Gi and GiPoll maps, respectively, at samplings S1, S2 and S3.

#### Sampling S1 (four months of growth)

After four months of growth (S1), the comparison between C and Poll plants showed that three spots were significantly up-regulated by the metal treatment (104, 485, 491), and one (230), was down-regulated (Figures 1A and 4, Table 4).

The effect of AM inoculation was more marked (comparison: Gi vs. C), as five spots were up-regulated (247, 283, 304, 314, 397) and eleven were down-regulated (112, 124, 130, 153, 154, 165, 470, 471, 484, 491, 494), with most of the proteins involved in sugar - energy metabolism and protein folding.

The effect of metals on AM plants (comparison: GiPoll vs. Gi) resulted in the up- and down-regulation of one (484, a BiP isoform) and four (283, 304, 397, 470) spots respectively. Finally, the AM fungus modulated the proteome of plants grown on metal polluted soil (comparison: GiPoll vs. Poll) causing the up-regulation of four identified spots (230, 247, 314, 397), and the down-regulation of nine spots (104, 124, 130, 154, 470, 471, 485, 491, 494), mostly related to protein synthesis and folding.

#### Sampling S2 (six months of growth)

Six months after the establishing of the cultures, towards the end of the growing season (early autumn), the heavy metal treatment on non-mycorrhizal plants (comparison: Poll vs. C) was strongly inhibitory (Figures 1B and 5, 6, 7, 8, Table 5), as only one spot was significantly up-regulated [Ribose-5-phosphate isomerase (255)], while thirty-one were down regulated [seven isoforms of RuBisCO activase (118, 119, 137, 142, 171, 294, 420), ten proteins associated with photosynthesis and carbohydrate metabolism (152, 155, 181, 272, 291, 409, 410, 411, 415, 423), and seven proteins involved in oxidative stress response (164, 202, 246, 254, 403, 414, 419); moreover seven other spots were down-regulated (132, 146, 149, 161, 172, 275, 402)].

In the absence of metals, the effect of the AM symbiosis (comparison: Gi vs. C) pointed out the modulation of twenty-six identified spots, sixteen up-regulated (122, 134, 137, 146, 150, 161, 162, 163, 164, 165, 166, 171, 174, 253, 275, 421, many of them concerning carbohydrate and energy metabolism), and ten down-regulated (202, 269, 272, 291, 402, 403, 409, 410, 411, 423, mostly involved in photosynthesis and oxidative stress response).

In mycorrhizal plants, the metal treatment (comparison: GiPoll vs. Gi) led to the down-regulation of thirty-two spots [eight RuBisCO activases (118, 119, 134, 135, 137, 142, 171, 294), twelve proteins connected to photosynthesis, carbohydrate and energy metabolism (122, 146, 152, 155, 162, 163, 166, 168, 174, 181, 409, 415), four proteins implicated in oxidative stress response (164, 246, 254, 414), and eight miscellaneous proteins (150, 161, 172, 193, 245, 253, 275, 421)].

When plants were grown on heavy metal polluted soil (GiPoll vs. Poll), the inoculation with *G. intraradices* significantly up-regulated eight spots (148, 149, 171, 181, 202, 272, 291, 402), while two were down regulated (255, 269).

#### Sampling S3 (sixteen months of growth)

Just before the plant harvest, after sixteen months of growth (corresponding to the second growing season), the effect of heavy metals on non-mycorrhizal plants (comparison: Poll vs. C) was shown by the up-regulation of two spots [an ATP synthase beta subunit (132) and a phosphoribulokinase (171)] and the down-regulation of twenty-six proteins, [among them ten proteins belonging to photosynthesis and carbohydrate metabolism (199, 215, 270, 277, 279, 394, 487, 603, 610, 611), and four proteins linked to oxidative stress (290, 310, 320, 334); the remaining spots being: 236, 261, 289, 293, 295, 301, 308, 313, 314, 315, 600, 602] indicating again a generally inhibitory effect of the metals on protein expression (Figures 1C, 9, 10 and Table 6).

Considering the effects of *G. intraradices* on plants grown on control soil (comparison: Gi vs. C), the fungal colonization promoted the up-regulation of five spots (105, 270, 361, 363, 384), of which three were heat shock proteins, and the down-regulation of twenty-seven [among them twelve proteins from photosynthesis and carbohydrate metabolism (178, 199, 215, 223, 227, 279, 394, 487, 603, 610, 611, 613), three proteins of the oxidative stress response (290, 320, 334), and two proteins involved in protein folding (118, 601); the remaining ten spots were 236, 244, 261, 293, 301, 308, 315, 332, 600, 602].

In mycorrhizal plants, the growth on polluted soil (comparison: GiPoll vs. Gi) resulted in the up-regulation of three spots [a Hsp70 (85), a small Hsp (361) and RuBisCO large subunit (384)] and in the down-regulation of forty-three spots [sixteen proteins involved in photosynthesis and carbohydrate metabolism (176, 178, 199, 200, 209, 212, 216, 223, 279, 346, 394, 487, 594, 603, 611, 613), seven proteins of the oxidative stress response (241, 310, 320, 333, 334, 598, 614), three proteins implicated in protein folding (105, 319, 601); seventeen further spots were down-regulated: 197, 236, 238, 247, 261, 286, 289, 295, 301, 305, 308, 313, 314, 315, 317, 332, 600)].

Finally, when plants where grown on polluted soil (GiPoll vs. Poll), the AM symbiosis resulted in the up-regulation of five spots [a Hsp70 (85), two RuBisCO large subunits (270, 384), a small Hsp (361, 363)] and down-regulation of forty-seven spots [nineteen proteins concerning photosynthesis and carbohydrate metabolism (118, 132, 171, 176, 178, 199, 200, 209, 212, 216, 223, 227, 279, 346, 394, 487, 603, 611, 613), seven proteins of the oxidative stress response (241, 310, 320, 333, 334, 598, 614); the remaining twenty-one other proteins were: 197, 236, 238, 244, 247, 261, 286, 289, 293, 295, 301, 305, 308, 313, 314, 315, 317, 319, 332, 600, 601].

## Discussion

This long term experiment clearly showed the success of phytoremediation by mycorrhizal poplars, as both copper and zinc concentrations in soil were significantly reduced (Table 3). This is in accord with previous studies on poplar inoculated with different species of AM fungi [20], [40], [41]. Moreover, on polluted soil, fungal inoculation restored root and stem biomass, with the exception of leaf biomass (Table 1). It is worth mentioning that the present results are part of a project aiming at the optimization of a phytoremediation system including selected poplar clones and AM fungi. Plants of clone AL35 had been chosen for their ability to survive on metal-polluted soil and accumulate copper and zinc in their organs [9]. Therefore, the AM fungus modulated the proteome of a clone which is already metal tolerant.

It is well known that different metals are accumulated in different plant organs depending on the plant species [42]. In this case Cu was mainly accumulated in roots, and Zn in leaves. This metal distribution in poplar is in agreement with previous reports [6], [9], [20], [43], [44].

Cu accumulation in leaves was very low, consistent with the scarce translocation of this element to the shoot [11], [45], [46]. On the contrary, AM fungi enhanced zinc translocation in leaves from contaminated soil, in agreement with previously published results [20], [47]–[49]. The highest levels of zinc in the leaves were recorded at sampling S2 (September of the first growing season) when the leaves were mature but not as yet senescing. A similar increase of leaf metal concentration in relation to the plant age was also observed in *Aesculus hippocastanum* grown in a polluted site [50].

AM fungi did not enhance P concentration in the first growth season (i.e. S1 and S2), while they did in the second (S3). In fact, at the end of experiment, inoculation with *G. intraradices* improved phosphate nutrition either in plants grown on polluted or non-polluted soil. Implication of endomycorrhizal fungi in plant uptake of macronutrients as P has been widely demonstrated [26], [51], [52].

### At the First Sampling (S1) Leaf Proteome was Modified by AM Fungi

At sampling S1, the AM symbiosis modified leaf protein expression more than heavy metals. Mycorrhization induced a decrease of ATP synthase isoforms, Kieffer et al. [15], [16] reported a similar decrease on cadmium-exposed poplars. Moreover enolase expression was strongly inhibited by fungal colonization, together with a form of RuBisCO, while a specific fragment of RuBisCO was increased in the presence of AM fungi. Enolase is a multifunctional enzyme, responsive to many environmental stresses [11], [53]. The effect of mycorrhization on sugar metabolism is also underlined by the increase of fructose bisphosphate aldolase and NAD-epimerase/dehydratase, whose corresponding spots have been detected also in sampling S2 and S3. It is interesting that during this long-term exposure both proteins became progressively down-regulated.

The other class of proteins which characterized the proteomic change of sampling S1 belonged to protein folding. Heat shock proteins (Hsp) respond to various stresses in different plants, with specific pattern of expression [54]. These proteins are modulated not only by abiotic stresses but also during AM symbiosis, as demonstrated for the fronds of *P. vittata* [38]. In poplars, polluted soil induced the increase of one isoform of Hsp 70 (spot 485), while another isoform of Hsp 70 (spot 112) was decreased by *G. intraradices* colonization. At the same time, the BiP isoform (spot 484) and the Hsp 17 were down regulated by mycorrhization. BiP is a widely distributed and highly conserved member of the HSP70 family of molecular chaperones. Many biotic and abiotic stresses induce the accumulation of unfolded proteins in the ER that irreversibly bind BiP; this is thought to reduce the number of free BiP molecules leading to the induction of BiP transcription [55], [56]. BiP overexpression confers resistance to drought, as demonstrated by Valente et al. [57] in soybean and tobacco.

Laccase-8 (spot 491) is another example of protein affected by mycorrhization in poplar leaves, in fact it was down-regulated in both Gi and GiPoll plants, while it was up-regulated in Poll plants in respect to the controls. Laccases, or p-diphenol: O_2_ oxido-reductases, are copper-containing glycoproteins [58], in this case the up-regulation could be a strategy to detoxify copper. In plants, the role of laccases has not fully been clarified; however, based on their capacity to oxidize lignin precursors (p-hydroxycinnamyl alcohols), and their localization in lignifying xylem cell walls [59], [60] their involvement in lignin biosynthesis has been suggested [61]. The up-regulation of laccase in plants grown on polluted soil is in agreement with data published by Todeschini et al. [33], reporting cell wall modifications in plants treated with heavy metals.

The thiamine biosynthetic enzyme (THP) (spot 283) was up-regulated in Gi plants in respect to the controls and was down-regulated in GiPoll plants in respect to Gi ones. Thiamin pyrophosphate (TPP) is an essential cofactor required by enzymes involved in the intermediary metabolism [62]. Thiamin has been reported to alleviate the effects of several environmental stresses in plants. The exogenous application of thiamin was shown to counteract the harmful effects of salinity on growth [63] and to confer resistance to fungal, bacterial, and viral infections of *Oryza sativa*, *Arabidopsis thaliana* and in some crop species [64]. Thiamin was also implicated in responses to stress conditions such as sugar deprivation and hypoxia in *Arabidopsis* [65]. Protein levels of the important thiamin biosynthetic enzyme are modulated upon heat stress in *Populus euphratica* [37], and the rice homolog of this enzyme is connected to disease resistance [66], [67]. Under our experimental conditions, the up-regulation of THP could be linked with the observed better general conditions of Gi plants.

Epsin (spot 230) was down-regulated in Poll plants but up-regulated in GiPoll (fungus effect). Epsin plays important roles in various steps of protein trafficking in animal and yeast cells. It is involved in the trafficking of soluble proteins to the central (lytic) vacuole in *Arabidopsis* [68].

### At the Second Sampling (S2) Leaf Proteome was Strongly Modified by Metals

At sampling S2, when zinc concentration was highest in the leaves, data from Poll plants clustered separately from the others, indicating a strong effect of the metals. Several enzymes involved in carbon fixation were down-regulated, as was previously observed in rice leaves [69], in poplar leaves treated with cadmium [14] and reviewed by Ahsan et al., [70]. Soil pollution caused the consistent down-regulation of 66% of the identified proteins, of these 23% were isoforms of RuBisCo activase; the only up-regulated protein was a ribose-5-phosphate isomerase. The same down-regulation trend was repeated also in GiPoll plants, when mycorrhizal plants were grown in polluted soil. A characteristic pattern of expression has been identified for the two forms of phosphoglycerate kinase, with an increase in presence of the fungal colonization and a decrease induced by pollution, suggesting a strategy of “buffer defense” induced by AM fungi, which could help the plants in reacting against metal stress. The same trend is observed also for some forms of RuBisCO activase, aldoketo reductase, uroporphyrinogen decarboxylase, malate dehydrogenase and fructose bisphosphate aldolase, suggesting a protective role of AM fungi towards primary metabolism. Malate dehydrogenase has recently been identified as one of the ten drought-responsive phosphoproteins in rice [71] and as a target of arsenic stress in *P. vittata* fronds [38]. Uroporphyrinogen decarboxylase (UroD) catalyses the decarboxylation of uroporphyrinogen III to give coproporphyrinogen III in the heme and chlorophyll biosynthesis pathway(s). In wheat, the UroD protein abundance increased in response to both light and heat. The UroD content substantially declined under chill stress [72]. Also geranylgeranyl diphosphate (GGDP) synthase (spot 193), involved in the carotenoid biosynthetic pathway, was down regulated in GiPoll plants. A large variety of products are derived from isoprenoids in plants for their growth and response to environmental changes [73]. Geranylgeranyl diphosphate (GGPP) is one of the key isoprenoids to be converted into compounds necessary for plant growth, such as gibberellins, carotenoids, chlorophylls, isoprenoid quinones, and geranylgeranylated small G proteins such as Rho, Rac, and Rab [74], [75].

One of the key enzymes in nitrate assimilation leading to biosynthesis of glutamate, glutamine synthetase (spot 149), was also down-regulated by heavy metals, indicating that also the amino acid biosynthesis pathways were affected by heavy metals.

Moreover heavy metal pollution led to the down-regulation of proteins related to oxidative stress response, like three isoforms of ascorbate peroxidase (246, 254, 414), a superoxide dismutase (419) and the aldo/keto reductase (spot 164); this one has been described as responsive to HM stress in leaves [76], [77]. As shown by pie charts (Figure 3), the proteins involved in “Oxidative damage” and “Glutathione metabolism” are represented only at samplings S2 and S3 but not at the first one (S1).

Esterase d, S-formylglutathione hydrolase (spot 402), was down-regulated in Poll and Gi plants, but the simultaneous presence of fungal colonization and polluted soil (GiPoll) led to its up-regulation. This enzyme is involved in the detoxification of formaldehyde. In most prokaryotes and all eukaryotes, formaldehyde is detoxified by a three-step process [78], [79]. First, formaldehyde reacts spontaneously with glutathione, the major free cellular thiol, to form S-hydroxymethylglutathione. This glutathione adduct is then oxidized to S-formylglutathione by formaldehyde dehydrogenase [80]. Finally the S-formylglutathione is hydrolyzed to glutathione and formic acid by S-formylglutathione hydrolase (SFGH). Another protein of glutathione metabolism was differentially expressed at sampling S2, a form of glutathione transferase is down-regulated by AM fungi. Data on the glutathione metabolism enzymes have been previously reported on cadmium-treated poplars [14], [16].

### At S3 Sampling the Simultaneous Presence of AM Fungi and Metal Pollution Affected Leaf Proteome

At the last sampling, data from mycorrhizal plants grown on polluted soil clustered independently, showing a peculiar proteome profile induced by the simultaneous presence of both AM and HM. In particular, Hsp up-regulation in mycorrhizal plants, with or without metal presence, was confirmed and involved different isoforms. Under our growth conditions, the simultaneous presence of heavy metals and AM symbiosis induced a general down regulation of leaf proteins, confirmed by morphological data, as leaf dry weight was low even in the presence of mycorrhiza. In the leaf, negative effect on carbon fixation protein expression was salient, especially on ribulose-1,5-bisphosphate carboxylase/oxygenase (RuBisCO) and RuBisCO activase; moreover some enzymes involved in the light phase of photosynthesis were negatively affected. This result is in agreement with those of Durand et al. [81], showing cadmium effect on *Populus tremula* leaves. Moreover, at sampling S3 ATP synthase was up-regulated in Poll plants and down-regulated in GiPoll ones; this protein was also differentially expressed at sampling S1, which corresponded to the same season stage. On the contrary, at the end of the growing season (sampling S2) ATP synthase was not affected.

Other proteins down-regulated in Poll, Gi, and GiPoll plants were ferritin, glutathione transferase, a mitochondrial ATP synthase subunit and a drought induced stress protein. Ferritins are highly conserved proteins consisting of large multimeric shells that can store up to 4500 atoms of iron [82]. Ferritin can play a critical role in the cellular regulation of iron storage and homeostasis. While animal ferritins are mainly cytosolic proteins, the plant ones appear to be localized in chloroplasts of plant cells (or, more in general, in plastids) and in mitochondria [83]. Under conditions where iron is not a cause of stress, plant ferritin synthesis is developmentally regulated; it is almost undetectable in the plastids of vegetative organs like roots and leaves. However, in particular moments of the plant lifesuch as the time of fecundation, an activation of iron uptake at the root level has been observed, correlated with an accumulation of ferritin in flowers and developing seeds. Since in plants ferritins are localised in the plastids, they could play an important role in preventing oxidative damage by storing free iron in a safe form [84]. Such a hypothesis is supported by cytological studies that have demonstrated that an oxidising agent such as ozone induces ferritin accumulation in plants; the same results were obtained in a proteomic study in rice seedling after cold stress [85]. The poplar clone (AL35) used for this proteomic study shows constitutive ferritin over expression (in control plants) in mature leaves. These results could be linked with constitutive heavy metal tolerance demonstrated by this clone in a previous field study [9].

At the third sampling we observed a simultaneous mycorrhiza-metal induced down regulation of other enzymes involved in oxidative stress: aldo/keto reductase (241), ascorbate peroxidase (310, 598) and glutathione transferase DHAR (614), suggesting a stabilization/adaptation of the plant response under long term conditions of exposure to heavy metals. This result is in agreement with those demonstrated by Kieffer et al. [15], [16] that showed a reduction in ascorbate peroxidase after 56 days of cadmium treatment in poplar leaves. Ascorbate peroxidase (APX) plays a role in peroxide reduction by facilitating the oxidation of ascorbate. In literature it has been reported as an oxidative stress enzyme and its up regulation under stress condition is well documented in proteomic works [34] but different studies reported an APX down-regulation after, for example, cadmium stress [86].

It is noteworthy to highlight the down-regulation of a carboxymethylenebutenolidase by this study in GiPoll plants. This is the first time that the enzyme has been directly identified as a protein spot in a plant tissue. Carboxymethylenebutenolidase is an esterase involved in the degradation of aromatic compounds, it is poorly described in eukaryotes, while it has been described as a zinc dependent hydrolase in *Pseudomonas reinekei* [87].

Finally, three enzymes involved in fatty acids biosynthesis were down regulated in both Gi and GiPoll plants: isovaleryl-CoA dehydrogenase (238), pyruvate dehydrogenase acetyl-transferring (244) and 3-hydroxyisobutyrate dehydrogenase (308).

### Can the Leaf Proteome Explain the Plant Response to Metals and AM Fungi?

Our experimental design has been successful in the identification of a pattern of proteins involved in the leaf response to both AM colonization and metal stress.

However, the pattern is complex and the factor “time of sampling” has proven critical in giving rise to different changes in protein expression. It has not been possible to categorically identify the one or few proteins responsible for the phytoremediation activity of our biological system, caused by colonization of poplar by an AM fungus. The expectation of such a result is related to the fact that we anticipate a static picture of protein functions [88], while biochemical systems, like our poplar leaves, are dynamic. The proteomic approach represents one of the best tools to investigate dynamic changes in metabolism; the goal will be the integration of all the differently expressed proteins into a system of interacting enzymes. In doing this it is important to consider that the multifunctionality of proteins, frequently observed in proteomics [89], is fundamental for living organisms.

Considering all the differentially expressed proteins at sampling S2, we can point out a group of proteins sharing the same down-regulation pattern due to metal pollution; this group consists of some isoforms of RuBisCO activase (118, 119, 142), an ascobate peroxidase (414) and a phosphoribulokinase (155). In the literature it has been reported that phosphoribulokinase can be inhibited by the formation of supra-molecular complexes with other proteins under oxidizing conditions [90]. The same group of proteins (176, 178, 212, 598, 227) was down-regulated at sampling S3 too, in particular in GiPoll plants, indicating a long term response of the plant to metal stress and AM colonization. If our experiment had been limited to sampling S1, we could have never attributed the specific, above mentioned role to this group of proteins.

The results presently described are related to two previously published papers, reporting polyamine (PA) concentration and expression of the genes encoding for metallothioneins (MT) and for the enzymes involved in PA biosynthesis [17], and a transcriptome screening by cDNA-AFLP in leaves of poplar [91]. In both cases, the plants used for the experiments were exactly the same individuals used for this proteome analysis. MTs and PAs are not detectable with the techniques used in the present report; the concentration of free and conjugated PAs increases in plants inoculated with AM fungi and grown on polluted substrates. At the same time, the genes encoding for MTs and some of those involved in PA biosynthesis are overexpressed, resulting in restored growth (consistent with the report by Balestrazzi et al., [19] on the constitutive expression of a MT gene in poplar), comparable to that of plants grown on unpolluted soil [17]. The overall transcriptome study of four-month old plants [91] confirmed that both heavy metals and mycorrhiza affect gene expression in leaves, with different cDNA-AFLP patterns. Most of the affected genes are involved in secondary metabolism or in defense response [91]. The lack of a perfect match between transcriptome and proteome analyses had to be expected, because of the different sensitivity of the techniques and because of the post-transcriptional regulation mechanisms, and it confirms the necessity of a multi-technique approach in order to better understand the various responses of the plant.

Proteomic analysis (2-DE separation followed by MS protein identification) has been integrated with bioinformatic, statistical and cluster analyses (Figures 2, 3), the highlighted leaf responses were consistent with the general scheme of defence mechanisms triggered by heavy metals [70], involving changes in the abundance of chaperones, oxidative stress proteins and enzymes of primary metabolism. What distinguishes this work from other classical plant proteome studies is that this was the first long term experiment on a forestry plant grown on polluted soil and in the presence of an arbuscular mycorrhizal symbiosis. Our experimental system was very close to a real phytoremediation process. It was extremely interesting that the temporal feature affected the biological plant response: the first leaf reaction was dominated by the presence of AMF colonization, then it was the turn of the metals, and exactly one year after the first sampling, proteomic data were indicative of both a metal adaptation during the two years and a strong efficiency of mycorrhizal symbiosis in phytoextraction. These proteomic temporal features should be taken into account for the future development of metal tolerant plants.

## Materials and Methods

### Plant Material and Fungal Inoculation

The poplar clone *Populus alba* L. AL35 used in the present study was selected during a field trial [9] on a metal-polluted site, located next to the KME-Italy S.p.A. factory (Serravalle Scrivia, AL, Italy). Cuttings 20 cm long were collected from plants growing in the field. They were placed into 20 cm high plastic pots (750 mL) containing heat-sterilized (180°C, 3 h) quartz sand (3–4 mm diameter). Pots were inoculated with *Glomus intraradices* Schenck and Smith BB-E (supplied by Biorize, Dijon, France) as previously described [20], or were not inoculated (controls).

Inoculum was provided at 50% (v/v) concentration around each cutting, using a 50 mL bottomless Falcon tube around the cutting. Cuttings were fed on alternate days with 80 mL of Long Ashton solution, modified according to Trotta et al. [92]. After 1 month, the cuttings were transferred into sterilized 7.5 L plastic pots containing either polluted or unpolluted autoclaved soil (see below).

### Experimental Design and Growth Conditions

The soil originating from the above-mentioned polluted site is a sandy loam (according to USDA specifications) and has the following chemical features: organic matter 2.24% dry weight (d. wt); N 0.01 d. wt; K 0.0237% d. wt; P 0.0026% d. wt; pH 6.2, with a mean soil total zinc concentration of 950 mg kg-1 d. wt and 1300 mg kg-1 d. wt of copper [9]. The non-polluted soil, collected from a nearby unpolluted area, had similar features, and mean Zn and Cu concentrations of 60 and 14 mg kg-1 d. wt, respectively. The chemical analyses were carried out by inductively coupled plasma optic emission spectrometry (ICP-OES) as described in Lingua et al. [20]. The experimental design therefore consisted of growing the plants pre-inoculated or not with *G. intraradices* for two vegetative seasons (starting from March to July of the following year) in pots containing either polluted or non-polluted soil. Ten plants per treatment were prepared, placed in a greenhouse and automatically watered (from the top), before dawn, twice a week for 3 min; in July and August, plants were watered for 8 min on alternate days. Finally, four treatments were set up: C – un-inoculated plants grown on a control soil; Gi - plants inoculated with *G. intraradices*, grown on control soil; Poll - plants grown on polluted soil; GiPoll - plants grown on polluted soil and inoculated with *G. intraradices*.

Samples were taken as follows: first sampling, S1 (4-month-old plants, summer), second sampling, S2 (6-month- old plants, early autumn) and third sampling, S3 (end of experiment, 16-month-old plants, summer of the second year). In the first year, leaf samples, representative of the entire foliage of the plant (excluding the youngest unexpanded leaves), were taken from all plants in each treatment. In the second year, the whole plant was harvested; root, stem and leaf samples were collected and stored separately for fresh and dry weight measurements, and for the determination of Cu, Zn and P concentrations. The leaves from each treatment were pooled in order to have five biological repeats at each sampling time, frozen in liquid nitrogen and stored at –80°C for proteomic analyses or dried at 75°C up to constant weight for HM determinations.

### Chemical Analyses

Approximately 0.5 g d. wt from three biological replicates were used for the quantification of Cu, Zn and P in leaves, stems and roots, separately. Samples were digested, and their metal concentrations determined as described in Lingua et al. [20] by ICP-OES using an IRIS Advantage ICAP DUO HR series (Thermo Jarrell Ash, Franklin, MA, USA) spectrometer.

### Analysis of Growth and Mycorrhizal Colonisation

At the end of the experiment (S3 sampling), growth was evaluated on the basis of leaf, stem and root fresh and dry weights. The degree of mycorrhizal colonization of all plants, pre-inoculated or not, was evaluated microscopically using the method of Trouvelot et al. [93] on fifty 1 cm long root segments per plant. Microscopic observations were carried out at ×50–×630 magnifications. Results are expressed as intensity of colonization, i.e. percentage of colonized roots (M%). The production of arbuscules and vesicles was also investigated.

### Protein Extraction and Quantification

Protein extraction was performed according to Vâlcu and Schlink [94] with some modifications [39]. Nitrogen ground powder (about 2 g) was resuspended in 20 ml precooled (−20°C) precipitation solution (10% TCA and 20 mM DTT in acetone added with 1% Protease Inhibitor Cocktail for plant cell and tissue extracts (Sigma- Aldrich), DMSO solution). Proteins were precipitated overnight at −20°C and recovered by centrifugation (35000×g, 4°C). The pellet was dried for 10 min under vacuum, resuspended in solubilization buffer (7 M Urea, 2 M Thiourea, 100 mM DTT, 4% CHAPS, 2% v/v IPG Buffer (GE Healthcare Bio-Sciences, Cologno Monzese (MI), Italy) and centrifuged for 1 h at 16000×g, 4°C. Protein content of the sample was quantified by Bradford method [95].

### 2-DE, Image and Statistical Analysis

Isoelectric focusing (IEF) was performed on IPG strips in an IPG-Phor unit (GE Healthcare Bio-Sciences). For semi-preparative separations, 500 µg of protein extracts were mixed with a rehydration buffer (8 M urea, 4% (w:v) CHAPS, 18 mM DTT, 0.5% 3–10 IPG Buffer) and focused at 60 kVhs at 20°C on precast 13 cm linear pH 3–10 and 4–7. The second dimension was carried out with a Protean II Xi system (Bio-Rad); 12% gels were run at 10°C under constant amperage (30mA). Gels were stained with Blue Silver according to Candiano et al. [96].

The gels were scanned in a GS 710 densitometer (Bio-Rad). The gel images were recorded and computationally analyzed using Same Spot software (Progenesis).

The intensity of each protein spot was normalized relative to the total abundance of all valid spots. After normalization and background subtraction, a match set was created for all treatments.

For each treatment five replicates were run. The differential expression analysis was performed comparing the quantity of matched spots in the Poll gels versus the C gels, Gi gels versus control gels, GiPoll gels versus Gi and Poll gels. The program creates a quantitative table with all normalized optical spot densities. This OD raw data were used to perform an Analysis of Variance (ANOVA) to detect statistical differences between the quantitation of the same spot in all replicates. We performed a one way ANOVA, followed by a post-hoc F test, using StatView 4.5 (Abacus Concepts, Berkeley, CA, USA) and P<0.05 was adopted as the level of significance. A two-way ANOVA was also performed (with the same software) for each spot showing significant variations, in order to asses the effect of the polluted soil (factor named “metal”), of the mycorrhizal colonization (“fungus”) and of their interaction (metal x fungus).

A cluster analysis was performed for the optical densities of the differentially expressed spots for each replica using the software R (ver. 2.7.0) [97]; distances were calculated with the "Manhattan" method and a dendrogram was built with the "Ward" method.

### Protein Identification by nanoLC Coupled with Q TOF MS/MS

The peptide samples obtained from in gel trypsin digestion [98], were dried into a vacuum concentrator 5301 (Eppendorf, Hamburg, Germany) and stored at −20°C until nanoHPLC ESI-Q-TOF MS analysis.

All nanoHPLC MS/MS experiments were performed on a Q-Star XL (Applied Biosystems) connected to an Ultimate 3000 system equipped with a WPS-3000 autosampler and two low-pressure gradient micropumps LPG-3600 (LC Packings, Amsterdam, NL). Ultimate 3000 was controlled from Chromeleon (version 6.70 SP2a). The Q-Star mass spectrometer was controlled from the Analyst QS 1.1 software (Applied Biosystems). The peptide pellets were resuspended immediately before analysis in 10 µl of solvent A (95% v/v water, 5% v/v acetonitrile, 0.1% v/v formic acid). Five microliters of each sample were loaded and washed for 5 min onto the precolumn (300 µm i.d.×5 mm, C18 PepMap, 5 µm beads, 100 Å LC-Packings) using a flow rate of 30 µL/min solvent A via the LPG-3600 loading pump. The peptides were subsequently eluted at 300 nL/min from the precolumn over the analytical column (15 cm×75 µm, C18 PepMap100, 3 µm beads, 100 Å LC-Packings) using a 35 min gradient from 5 to 60% solvent B (5% v/v water, 95% v/v acetonitrile, 0.1% v/v formic acid) delivered by the LPG-3600 micro pump and splitted at a ratio 1∶1000 in the flow manager FLM-3100 (LC Packings). The total duration of the LC run was 65 min, including sample loading, column washing and equilibration.

The analytical column was connected with a 8 µm inner diameter PicoTip nano-spray emitter (New Objective, Woburn, MA) by a stainless steel union (Valco Instrument, Houston, TX) mounted on the nano-spray source (Protana Engineering, Odense, Denmark). The spray voltage (usually set between 1800 and 2100 V) was applied to the emitter through the stainless steel union and tuned to get the best signal intensity using standard peptides. The two most intense ions with charge states between 2 and 4 in each survey scan were selected for the MS/MS experiment.

The QStar-XL was operated in information-dependent acquisition (IDA) mode. In MS mode, ions were screened from 400 to 1800 m/z, and MS/MS data were acquired from 60–2000 m/z. Each acquisition cycle was comprised of a 1 sec MS and a 3 sec MS/MS. MS to MS/MS switch threshold was set to 10 counts per second (c.p.s.). All precursor ions subjected to MS/MS in the previous cycle were automatically excluded for 60 sec using a 3 a.m.u. window.

A script (Applied Biosystems) was used to generate Mascot (.mgf) files with peak lists from the Analyst 1.1 (.wiff) files. The IDA settings were as follows: default charge state was set to 2+, 3+, and 4+; MS centroid parameters were 50% height percentage and 0.05 a.m.u. merge distance; all MS/MS data were centroided, with a 50% height percentage and a merge distance of 0.05 a.m.u. The threshold peak intensity was set to 4 c.p.s. The MS/MS data from the protein sample was searched as a Mascot file against all entries in the public NCBInr database (http://www.ncbi.nlm.nih.gov/) using the on line Mascot search engine (http://www.matrixscience.com) [99], [100]. A final check was carried out on NCBInr 20091103, with 10107245 sequences and 3447514936 residuals. Carbamidomethylation of cysteine residues, oxidation of methionine, deamidation of asparagine and glutamine were set as a variable modification for all Mascot searches. One missed trypsin cleavage site was allowed, and the peptide MS and MS/MS tolerance was set to 0.25 Da for both.

## Supporting Information

Figure S1 2-DE maps of poplar leaf proteins stained with Blue silver, colloidal Coomassie. The gel of each replica is shown for four treatments (Control; Gi – plants inoculated with *G. intraradices*, grown on control soil; Poll – plants grown on polluted soil; GiPoll – plants grown on polluted soil and inoculated with *G. intraradices*).
(PDF)

Table S1 **List of poplar leaf proteins from the first sampling, identified by MS/MS analysis, including average ratio of protein abundance.** a) In brackets, corresponding spot number in the other samplings (manually checked and confirmed by MS/MS analysis). b) Number of identified peptides. c) Graphical representation of the average ratios of the protein abundance: Poll/C (1), Gi/C (2), GiPoll/Gi (3), GiPoll/Poll (4). Positive values are given as such, whereas negative values are given according to the following formula: given value  =  −1/ratio. Value exceeding ±2 are indicative of strong protein induction and reduction, respectively. Asterisks indicate a statistically significant average ratio.
(PDF)

Table S2 **List of poplar leaf proteins from the second sampling, identified by MS/MS analysis, including average ratio of protein abundance.** a) In brackets, corresponding spot number in the other samplings (manually checked and confirmed by MS/MS analysis). b) Number of identified peptides and sequence coverage. c) Graphical representation of the average ratios of the protein abundance: Poll/C (1), Gi/C (2), GiPoll/Gi (3), GiPoll/Poll (4). Positive values are given as such, whereas negative values are given according to the following formula: given value  =  −1/ratio. Value exceeding ±2 are indicative of strong protein induction and reduction, respectively. Asterisks indicate a statistically significant average ratio.
(PDF)

Table S3 **List of poplar leaf proteins from the third sampling, identified by MS/MS analysis, including average ratio of protein abundance.** a) In brackets, corresponding spot number in the other samplings (manually checked and confirmed by MS/MS analysis). b) Number of identified peptides and sequence coverage. c) Graphical representation of the average ratios of the protein abundance: Poll/C (1), Gi/C (2), GiPoll/Gi (3), GiPoll/Poll (4). Positive values are given as such, whereas negative values are given according to the following formula: given value  = −1/ratio. Value exceeding ±2 are indicative of strong protein induction and reduction, respectively. The presence of asterisk is indicative of a statistically significant average ratio.
(PDF)

Table S4 **OD Values - first sampling (S1).** List of the spots showing significantly different average optical densities (± standard errors) and relative P values. Different letters indicate statistically significant differences (P<0.05).
(PDF)

Table S5 **OD Values - second sampling (S2).** List of the spots showing significantly different average optical densities (± standard errors) and relative P values. Different letters indicate statistically significant differences (P<0.05).
(PDF)

Table S6 **OD Values - third sampling (S3).** List of the spots showing significantly different average optical densities (± standard errors) and relative P values. Different letters indicate statistically significant differences (P<0.05).
(PDF)

Table S7 **Identification of poplar leaf proteins – first sampling (S1).** Precursor ion m/z, calculated peptide mass, ion score, modification, protein name, theoretical molecular weight and pI, accession number and reference organism, and blast results for each identified spot.
(PDF)

Table S8 **Identification of poplar leaf proteins – second sampling (S2).** Precursor ion m/z, calculated peptide mass, ion score, modification, protein name, theoretical molecular weight and pI, accession number and reference organism, and blast results for each identified spot.
(PDF)

Table S9 **Identification of poplar leaf proteins – third sampling (S3).** Precursor ion m/z, calculated peptide mass, ion score, modification, protein name, theoretical molecular weight and pI, accession number and reference organism, and blast results for each identified spot.
(PDF)

Table S10 **BLAST results – first sampling (S1).** Protein name, accession number and reference organism, BLAST results, percentage of homology, and percentage of identity.
(PDF)

Table S11 **BLAST results – second sampling (S2).** Protein name, accession number and reference organism, BLAST results, percentage of homology, and percentage of identity.
(PDF)

Table S12 **BLAST results – third sampling (S3).** Protein name, accession number and reference organism, BLAST results, percentage of homology, and percentage of identity.
(PDF)

Table S13 **Two-way ANOVA – first sampling (S1).** List of the spots showing significant P values for the two-way ANOVA for the factors Fungus, Metal or Fungus×Metal. Empty cells in the table correspond to non-significant P-values.
(PDF)

Table S14 **Two-way ANOVA – second sampling (S2).** List of the spots showing significant P values for the two-way ANOVA for the factors Fungus, Metal or Fungus×Metal. Empty cells in the table correspond to non-significant P-values.
(PDF)

Table S15 **Two-way ANOVA – third sampling (S3).** List of the spots showing significant P values for the two-way ANOVA for the factors Fungus, Metal or Fungus×Metal. Empty cells in the table correspond to non-significant P-values.
(PDF)
