# Supplementary material for: Effects of Heavy Metals and Arbuscular Mycorrhiza on the Leaf Proteome of a Selected Poplar Clone: A Time Course Analysis
Source: PLoS One. 2012 Jun 26;7(6):e38662. doi: 10.1371/journal.pone.0038662 (PMC3383689; doi:10.1371/journal.pone.0038662)

Figure S1. **2-DE maps of poplar leaf proteins stained with Blue silver, colloidal Coomassie**. The gel of each replica is shown for four treatments (**Control**; **Gi** – plants inoculated with *G. intraradices*, grown on control soil; **Poll** – plants grown on polluted soil; **GiPoll** – plants grown on polluted soil and inoculated with *G. intraradices*).

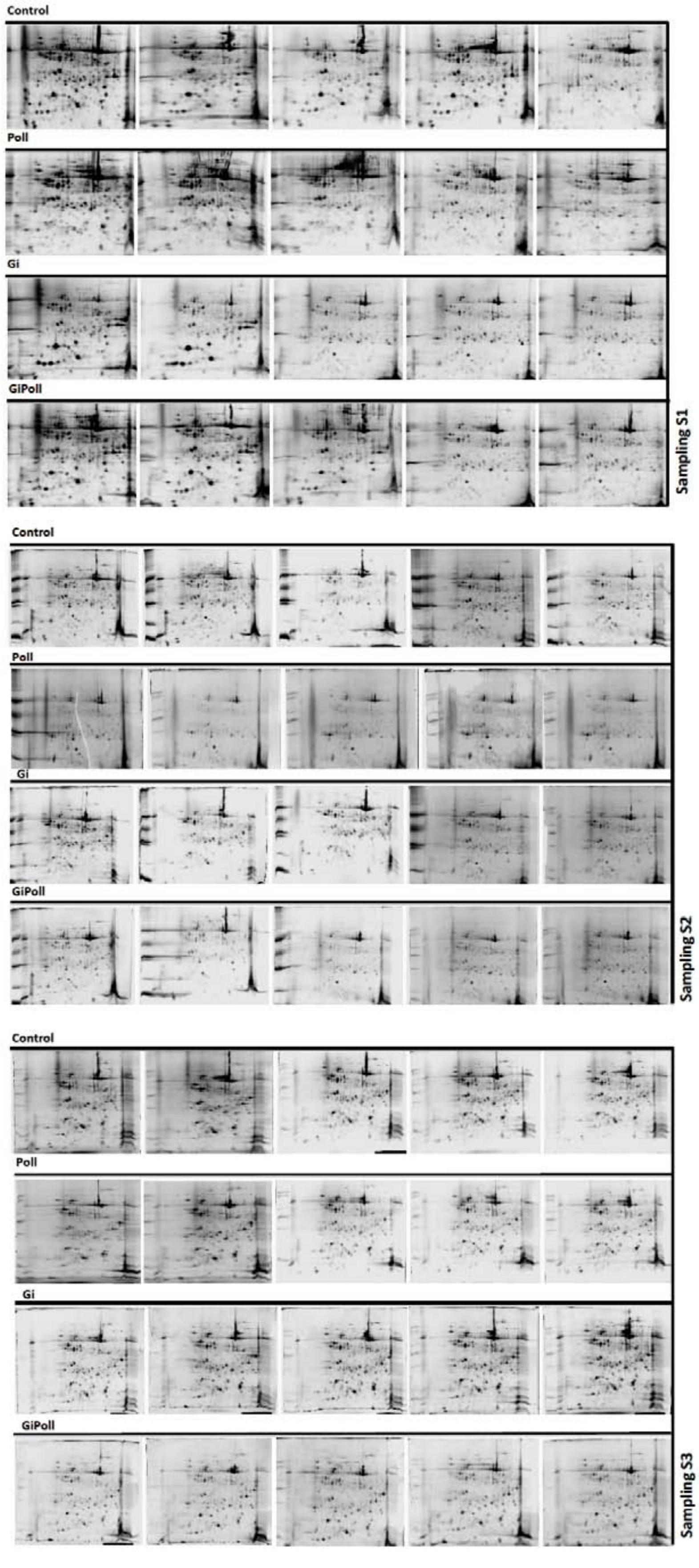

Supplement: Figure S1 — 2-DE maps of poplar leaf proteins stained with Blue silver, colloidal Coomassie. The gel of each replica is shown for four treatments (Control; Gi – plants inoculated with G. intraradices, grown on control soil; Poll – plants grown on polluted soil; GiPoll – plants grown on polluted soil and inoculated with G. intraradices). (PDF) [file pone.0038662.s001.pdf]
