# Supplementary material for: Effects of Heavy Metals and Arbuscular Mycorrhiza on the Leaf Proteome of a Selected Poplar Clone: A Time Course Analysis
Source: PLoS One. 2012 Jun 26;7(6):e38662. doi: 10.1371/journal.pone.0038662 (PMC3383689; doi:10.1371/journal.pone.0038662)
Supplement: Table S1 — List of poplar leaf proteins from the first sampling, identified by MS/MS analysis, including average ratio of protein abundance. a) In brackets, corresponding spot number in the other samplings (manually checked and confirmed by MS/MS analysis). b) Number of identified peptides. c) Graphical representation of the average ratios of the protein abundance: Poll/C (1), Gi/C (2), GiPoll/Gi (3), GiPoll/Poll (4). Positive values are given as such, whereas negative values are given according to the following formula: given value = −1/ratio. Value exceeding ±2 are indicative of strong protein induction and reduction, respectively. Asterisks indicate a statistically significant average ratio. (PDF) [file pone.0038662.s002.pdf]

Table S1. List of poplar leaf proteins from the first sampling, identified by MS/MS analysis, including average ratio of protein abundance.

Spot (Cor.)[a)]	Pep.[b)]	Seq. Cov.	Protein (BLAST results)	M_r (kDa) / pI Theor	M_r (kDa) / pI Exp	AC number (gi NCBI) and reference organism	Protein expression Profile[c)]
**104_I**	2	6%	RuBisCO large subunit	52.9/6.14	70.0/5.68	gi|2961315 *Spigelia anthelmia*	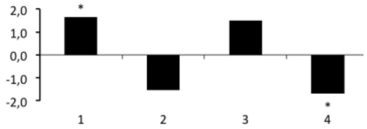
**112_I**	2	6%	Heat shock protein 70	71.4/5.07	71.1/5.13	gi|6911551 *Cucumis sativus*	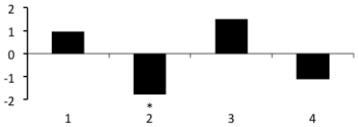
**124_I**	6	17%	ATP synthase beta subunit	51.8/5.20	71.0/5.20	gi|14718046 *Eucryphia lucida*	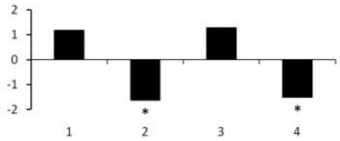
**130_I**	4	12%	Predicted protein (Enolase)	47.9/5.67	50.3/5.70	gi|224136806 *Populus trichocarpa*	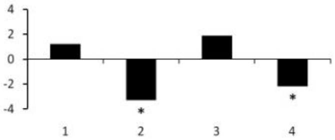
**153_I**	15	51%	ATP synthase beta subunit	53.6/5.09	62.6/4.92	gi|110227086 *Populus alba*	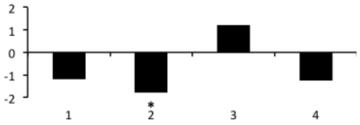
**154_I**	28	73%	ATP synthase beta subunit	53.6/5.09	62.6/5.15	gi|110227086 *Populus alba*	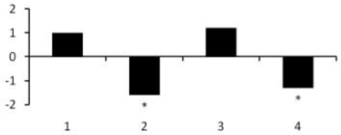
**165_I**	4	6%	RuBisCO large subunit	49.6/6.60	49.6/5.80	gi|46326306 *Salvia chamaedryo-ides*	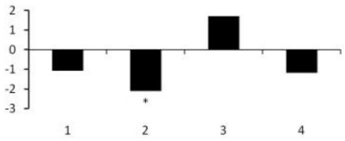

**230_I**	1	2%	Putative clathrin binding protein (epsin)	30.8/9.30	45.9/5.64	gi|3763925 *Arabidopsis thaliana*	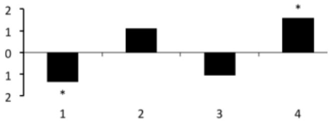
**247_I** **(174_II)** **(613_III)**	5	21%	Unknown (Fructose bisphosphate aldolase)	42.9/8.17	43.5/6.24	gi|118489355 *Populus trichocarpa x Populus deltoides*	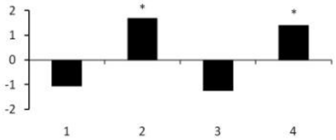
**283_I**	3	21%	Unknown (Thiamine biosynthetic enzyme)	29.3/5.26	38.7/5.74	gi|118488026 *Populus trichocarpa*	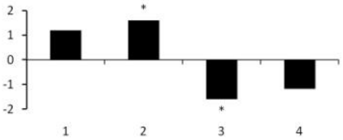
**304_I**	8	42%	Predicted protein	29.1/5.69	36.3/6.12	gi|224072767 *Populus trichocarpa*	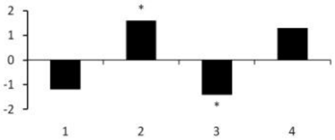
**314_I** **(245_II)** **(301_III)**	2	11%	Predicted protein (NAD-dependent epimerase / dehydratase)	27.0/5.68	38.8/5.30	gi|224090705 *Populus trichocarpa*	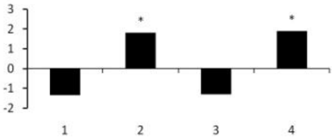
**397_I**	6	10%	RuBisCO large subunit	52.9/5.88	23.5/5.40	gi|1346967 *Brassica oleracea*	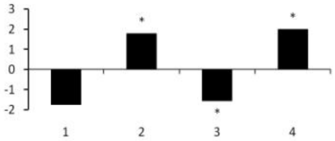
**470_I**	2	17%	Heat shock protein 17.0	17.0/5.78	17.0/6.47	gi|1122315 *Pennisetum glaucum*	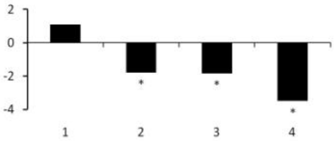
**471_I**	7	24%	Isomerase peptidyl-prolyl cis-trans isomerase	28.2/9.40	17.0/6.48	gi|224057792 *Populus trichocarpa*	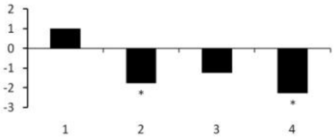
**484_I**	2	4%	BiP isoform B	73.4/5.11	73.4/5.11	gi|475600 *Glycine max*	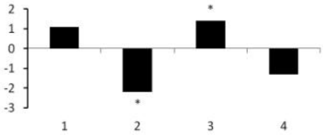

**485_I**	4	8%	Unknown (Hsp70)	71.1/5.10	71.1/5.10	gi|219885633 *Zea mays*	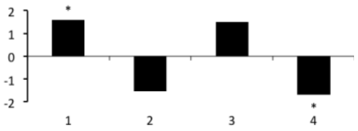
**491_I**	2	1%	Hypothetical protein SORBIDRAFT _03g039980 (Laccase-8)	60.2/6.49	43.9/4.89	gi|242054991 *Sorghum bicolor*	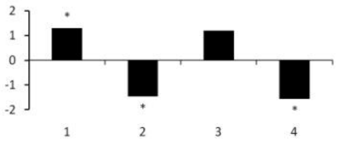
**494_I**	4	11%	Predicted protein (Elongation factor Tu)	52.7/6.00	53.7/5.37	gi|224053971 *Populus trichocarpa*	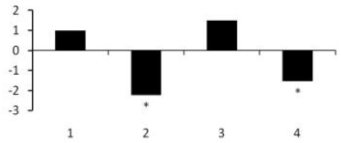

a) In brackets, corresponding spot number in the other samplings (manually checked and confirmed by MS/MS analysis).

b) Number of identified peptides

c) Graphical representation of the average ratios of the protein abundance: Poll/C (1), Gi/C (2), GiPoll/Gi (3), GiPoll/Poll (4). Positive values are given as such, whereas negative values are given according to the following formula: given value = -1/ratio. Value exceeding ± 2 are indicative of strong protein induction and reduction, respectively. Asterisks indicate a statistically significant average ratio.
