# Supplementary material for: Effects of Heavy Metals and Arbuscular Mycorrhiza on the Leaf Proteome of a Selected Poplar Clone: A Time Course Analysis
Source: PLoS One. 2012 Jun 26;7(6):e38662. doi: 10.1371/journal.pone.0038662 (PMC3383689; doi:10.1371/journal.pone.0038662)
Supplement: Table S2 — List of poplar leaf proteins from the second sampling, identified by MS/MS analysis, including average ratio of protein abundance. a) In brackets, corresponding spot number in the other samplings (manually checked and confirmed by MS/MS analysis). b) Number of identified peptides and sequence coverage. c) Graphical representation of the average ratios of the protein abundance: Poll/C (1), Gi/C (2), GiPoll/Gi (3), GiPoll/Poll (4). Positive values are given as such, whereas negative values are given according to the following formula: given value = −1/ratio. Value exceeding ±2 are indicative of strong protein induction and reduction, respectively. Asterisks indicate a statistically significant average ratio. (PDF) [file pone.0038662.s003.pdf]

Table S2. List of poplar leaf proteins from the second sampling, identified by MS/MS analysis, including average ratio of protein abundance.

Spot (Cor.)[a)]	Pep.[b)]	Seq. Cov.	Protein (BLAST result)	M_r (kDa) /pI Theor	M_r (kDa) /pI Exp	AC number (gi NCBI) and reference organism	Protein expression profile[c)]
**118_II** **(176_III)**	14	43%	Unknown (RuBisCO activase)	52.0/6.28	51.5/4.90	gi|118487547 *Populus trichocarpa*	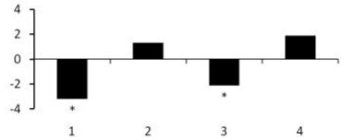
**119_II** **(178_III)**	12	38%	Unknown (RuBisCO activase)	52.0/6.28	51.5/5.00	gi|118487547 *Populus trichocarpa*	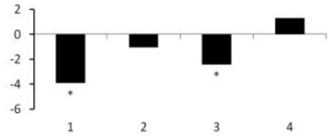
**122_II**	4	13%	Predicted protein (Phospho-glycerate kinase)	50.2/8.25	51.5/5.90	gi|224109060 *Populus trichocarpa*	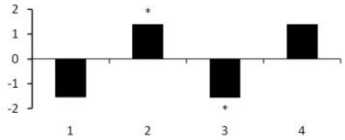
**132_II**	1	3%	Elongation factor Tu	52.1/6.21	50.0/5.50	gi|2494261 *Glycine max*	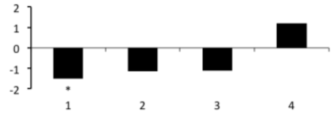
**134_II** **(199_III)**	10	31%	Unknown (RuBisCO activase)	50.6/8.36	51.9/4.90	gi|118489408 *Populus trichocarpa x Populus deltoides*	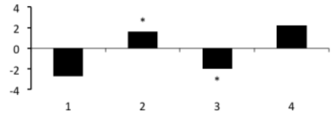
**135_II** **(200_III)**	9	23%	Unknown (RuBisCO activase)	51.9/5.26	51.9/4.90	gi|118486739 *Populus trichocarpa*	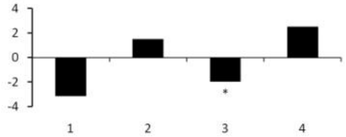
**137_II**	10	25%	Unknown (RuBisCO activase)	52.1/6.28	51.9/5.00	gi|118487547 *Populus trichocarpa*	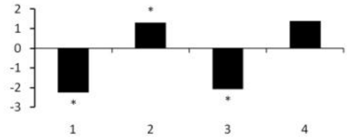

**142_II**<br><br>**(212_III)**	11	18%	Unnamed protein product (RuBisCO activase)	51.9/5.15	51.9/5.15	gi|157345989<br><br>*Vitis vinifera*	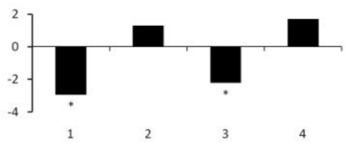
**146_II**<br><br>**(216_III)**	12	36%	Predicted protein (Phospho-glycerate kinase)	50.2/8.25	48.6/5.90	gi|224109060<br><br>*Populus trichocarpa*	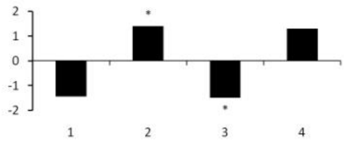
**148_II**	1	1%	Putative plastid isopentenyl diphosphate/dimethylallyl diphosphate synthase precursor	49.9/5.38	51.8/4.90	gi|209402463<br><br>*Mantoniella squamata*	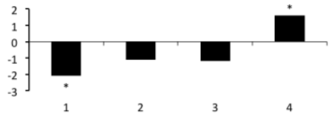
**149_II**	5	27%	Predicted protein (Glutamine synthetase)	39.2/5.52	47.7/6.15	gi|224079530<br><br>*Populus trichocarpa*	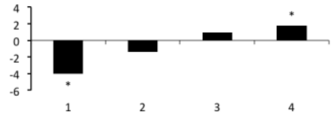
**150_II**	3	10%	Predicted protein (Uroporphy-rinogen decarboxylase)	44.5/7.14	47.7/6.85	gi|224145917<br><br>*Populus trichocarpa*	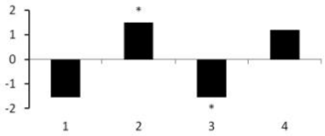
**152_II**	8	30%	Predicted protein (Phospho-ribulokinase)	45.0/5.90	51.8/5.00	gi|224071429<br><br>*Populus trichocarpa*	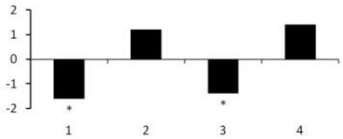
**155_II**<br><br>**(227_III)**	11	40%	Predicted protein (Phosphoribulo kinase)	45.0/5.90	51.8/5.15	gi|224071429<br><br>*Populus trichocarpa*	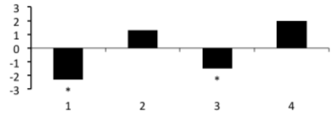
**161_II**	3	13%	Unknown (Protein disulfide isomerase, putative)	34.9/5.31	46.5/5.70	gi|118482960<br><br>*Populus trichocarpa*	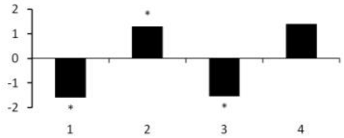
**162_II**	11	46%	Predicted protein (Malate dehydro-genase)	35.7/6.11	45.0/6.25	gi|224102193<br><br>*Populus trichocarpa*	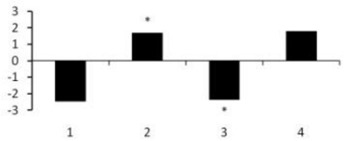

**163_II**	3	14%	Cytosolic malate dehydrogenase	35.5/5.92	44.9/6.30	gi|10334493<br><br>*Cicer arietinum*	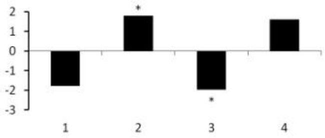
**164_II**	7	22%	Predicted protein (Aldo/keto reductase AKR)	37.4/5.97	45.7/6.14	gi|224069096<br><br>*Populus trichocarpa*	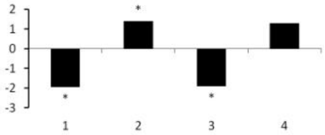
**165_II**	2	7%	Cytosolic malate dehydrogenase	35.5/5.92	44.9/6.53	gi|10334493<br><br>*Cicer arietinum*	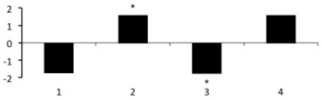
**166_II**	3	12%	Cytosolic malate dehydrogenase	35.5/5.92	44.9/6.80	gi|10334493<br><br>*Cicer arietinum*	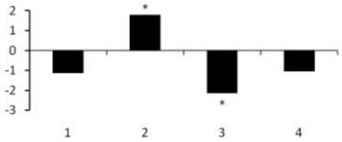
**171_II**	2	15%	RuBisCO activase	25.9/5.01	45.6/5.28	gi|100380<br><br>*Nicotiana tabacum*	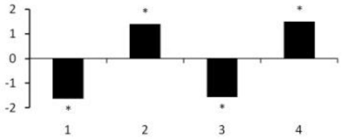
**172_II**	2	6%	Hypothetical protein	20.1/5.54	44.9/6.40	gi|147835353<br><br>*Vitis vinifera*	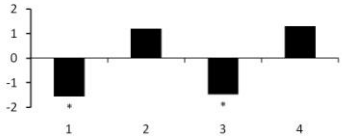
**174_II**<br><br>**(247_I)**<br><br>**(613_III)**	3	14%	Unknown (Fructose-bisphosphate aldolase)	42.9/8.17	44.9/6.21	gi|118489355<br><br>*Populus trichocarpa x Populus deltoides*	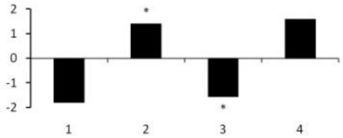
**181_II**	3	15%	Unknown (Fructose-bisphosphate aldolase)	42.8/7.55	44.0/6.08	gi|118487575<br><br>*Populus trichocarpa*	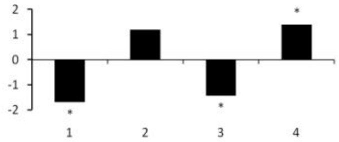
**193_II**	2	8%	GGDP synthase	39.2/5.38	41.4/5.52	gi|9971808<br><br>*Tagetes erecta*	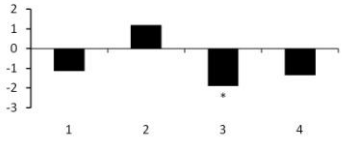

**202_II**	3	9%	Ferredoxin-NADP+ reductase	40.1/8.66	40.0/6.40	gi|5730139 *Arabidopsis thaliana*	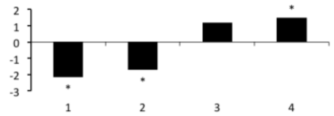
**245_II** **(314_I)** **(301_III)**	4	25%	Predicted protein (NAD-dependent epimerase / dehydratase)	27.0/5.68	33.9/5.34	gi|224090705 *Populus trichocarpa*	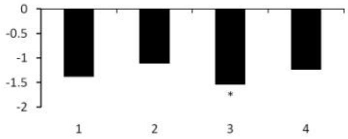
**246_II**	7	41%	Predicted protein (Ascorbate peroxidase)	27.3/5.53	34.1/5.70	gi|224104631 *Populus trichocarpa*	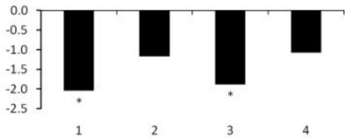
**253_II** **(319_III)**	3	18%	Predicted protein (Groes chaperonin)	27.1/7.77	32.0/5.22	gi|224141565 *Populus trichocarpa*	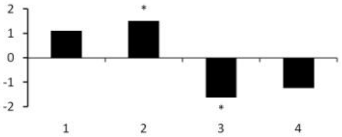
**254_II**	5	39%	Putative ascorbate peroxidase	22.4/4.83	32.0/5.70	gi|46911557 *Populus x canadensis*	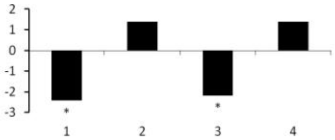
**255_II**	6	42%	Predicted protein (Ribose-5-phosphate isomerase, putative)	30.9/5.36	32.0/4.60	gi|224130670 *Populus trichocarpa*	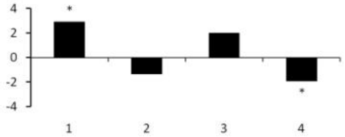
**269_II**	8	20%	Predicted protein (Tau class glutathione transferase)	25.4/5.31	29.7/5.33	gi|224117556 *Populus trichocarpa*	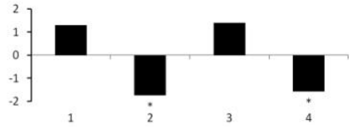
**272_II**	3	22%	Hypothetical protein POPTRDRAFT _551203 (Photosystem II reaction center psbP Protein)	28.2/7.68	29.7/6.53	gi|224062595 *Populus trichocarpa*	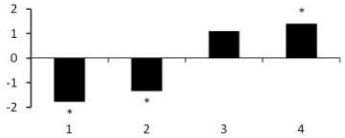
**275_II**	3	9%	Predicted protein (ATP-dependent Clp protease)	32.7/6.79	27.0/6.00	gi|224068558 *Populus trichocarpa*	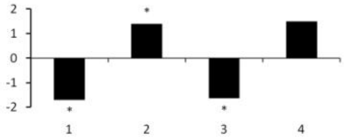

**291_II**	7	33%	Hypothetical protein POPTRDRAFT _818640 (Probable oxygen-evolving enhancer protein 2)	28.1/8.65	24.3/6.54	gi|224085421 *Populus trichocarpa*	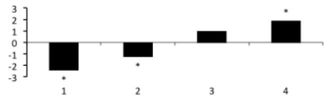
**294_II**	3	12%	RuBisCO activase precursor	40.8/7.59	23.5/5.33	gi|3687652 *Datisca glomerata*	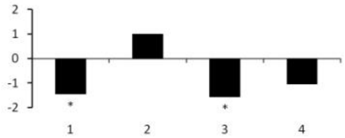
**402_II**	2	10%	Esterase d, s-formylglutathio ne hydrolase	31.9/6.17	40.0/6.80	gi|224086942 *Populus trichocarpa*	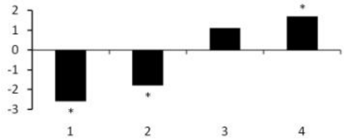
**403_II**	8	28%	Predicted protein (Ferredoxin--NADP reductase)	40.4/8.71	40.2/6.85	gi|224074257 *Populus trichocarpa*	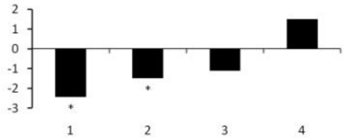
**409_II** **(603_III)**	2	27%	Putative protein (Oxygen-evolving enhancer protein 1)	18.5/5.17	38.3/5.10	gi|190898996 *Populus tremula*	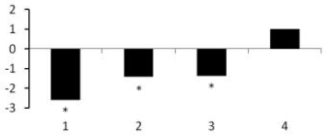
**410_II**	4	18%	Photosystem II protein 33kD	26.6/5.01	38.3/5.10	gi|224916 *Spinacia oleracea*	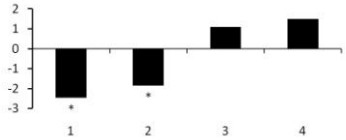
**411_II** **(610_III)**	11	37%	Unknown (Photosystem II oxygen-evolving complex 33)	35.1/5.62	35.1/5.17	gi|118489901 *Populus trichocarpa x Populus deltoides*	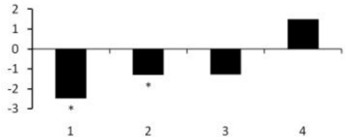
**414_II** **(598_III)**	3	14%	Ascorbate peroxidase	27.5/5.52	34.1/5.80	gi|42558486 *Rehmannia glutinosa*	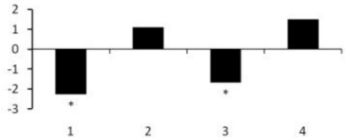

**415_II**	4	14%	Predicted protein (Protein THYLAKOID FORMATION1)	33.6/7.59	33.9/5.80	gi|224146717<br>*Populus trichocarpa*	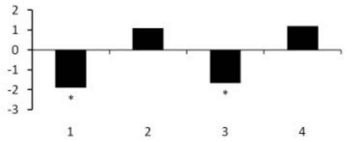
**419_II**	2	16%	Predicted protein (Manganese superoxide dismutase)	25.3/6.80	30.0/6.14	gi|224124440<br>*Populus trichocarpa*	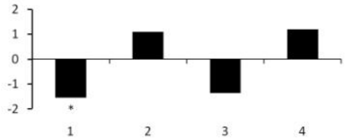
**420_II**<br>**(209_III)**	8	19%	Unknown (RuBisCO Activase)	52.0/6.28	48.3/5.31	gi|118489105<br>*Populus trichocarpa x Populus deltoides*	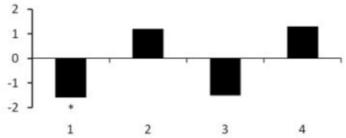
**421_II**	4	59%	Actin	17.2/4.73	48.8/5.31	gi|2887459<br>*Cucumis sativus*	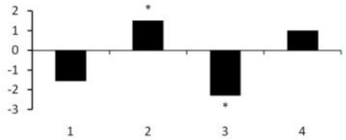
**423_II**	3	27%	Putative protein (OEE protein 1)	18.5/5.17	38.3/5.00	gi|190898996<br>*Populus tremula*	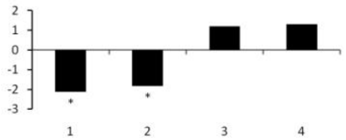

a) In brackets, corresponding spot number in the other samplings (manually checked and confirmed by MS/MS analysis).

b) Number of identified peptides and sequence coverage.

c) Graphical representation of the average ratios of the protein abundance: PolI/C (1), Gi/C (2), GiPolI/Gi (3), GiPolI/PolI (4). Positive values are given as such, whereas negative values are given according to the following formula: given value = -1/ratio. Value exceeding ± 2 are indicative of strong protein induction and reduction, respectively. Asterisks indicate a statistically significant average ratio.
