# Supplementary material for: Effects of Heavy Metals and Arbuscular Mycorrhiza on the Leaf Proteome of a Selected Poplar Clone: A Time Course Analysis
Source: PLoS One. 2012 Jun 26;7(6):e38662. doi: 10.1371/journal.pone.0038662 (PMC3383689; doi:10.1371/journal.pone.0038662)
Supplement: Table S3 — List of poplar leaf proteins from the third sampling, identified by MS/MS analysis, including average ratio of protein abundance. a) In brackets, corresponding spot number in the other samplings (manually checked and confirmed by MS/MS analysis). b) Number of identified peptides and sequence coverage. c) Graphical representation of the average ratios of the protein abundance: Poll/C (1), Gi/C (2), GiPoll/Gi (3), GiPoll/Poll (4). Positive values are given as such, whereas negative values are given according to the following formula: given value = −1/ratio. Value exceeding ±2 are indicative of strong protein induction and reduction, respectively. The presence of asterisk is indicative of a statistically significant average ratio. (PDF) [file pone.0038662.s004.pdf]

Table S3. List of poplar leaf proteins from the third sampling, identified by MS/MS analysis, including average ratio of protein abundance.

Spot (Cor.)[a)]	Pep[b)]	Cov.	Protein (BLAST results)	M_r (kDa) / pI Theor	M_r (kDa) / pI Exp	AC number (gi NCBI) and reference organism	Protein expression profile[c)]
**85_III**	2	3%	Heat shock 70 kDa protein	70.8/5.37	70.1/5.5	gi|123601 *Glycine max*	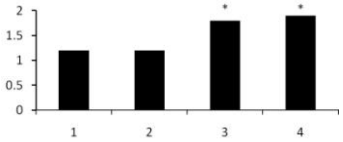
**105_III**	2	4%	Predicted protein (heat shock protein 70 (HSP70)-interacting protein, putative)	65.5/6.17	70.1/6.60	gi|224071575 *Populus trichocarpa*	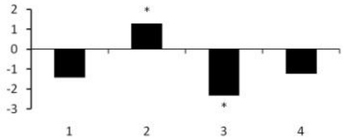
**118_III**	26	48%	Predicted protein (putative rubisco subunit binding-protein alpha subunit (Chaperonin))	62.0/5.24	62.0/5.24	gi|224104681 *Populus trichocarpa*	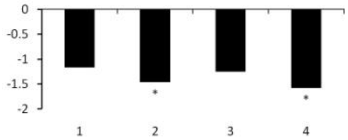
**132_III**	17	45%	ATP synthase beta subunit	52.0/5.05	52.0/5.05	gi|62085107 *Cespedesia bonplandii*	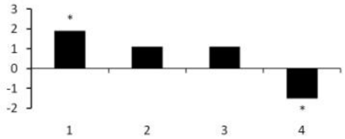
**171_III**	7	19%	Predicted protein (Phospho-ribulose kinase, putative)	45.0/6.11	51.0/4.96	gi|224138316 *Populus trichocarpa*	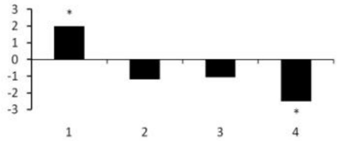
**176_III** **(118_II)**	21	44%	Unknown (RuBisCO activase 1)	52.0/6.28	50.5/4.96	gi|118489105 *Populus trichocarpa x Populus deltoides*	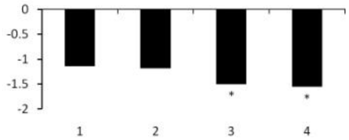
**178_III** **(119_II)**	13	24%	RuBisCO activase	48.0/8.20	50.5/5.03	gi|3914605 *Malus x domestica*	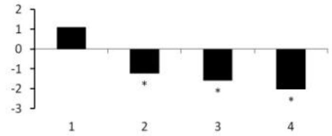

**197_III**	6	20%	Predicted protein (EF-Tu protein)	46.6/5.60	49.5/5.50	gi|224074859<br>*Populus trichocarpa*	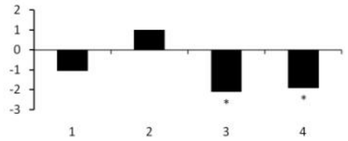
**199_III**<br>**(134_II)**	18	37%	Unknown (RuBisCO activase (RCA))	50.7/8.36	49.5/4.94	gi|118489408<br>*Populus trichocarpa x Populus deltoides*	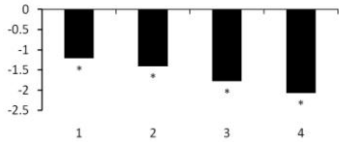
**200_III**<br>**(135_II)**	5	13%	RuBisCO activase 2	48.3/5.06	49.5/4.96	gi|12620883<br>*Gossypium hirsutum*	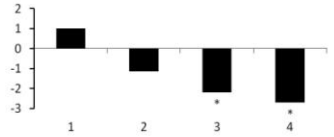
**209_III**<br>**(420_II)**	19	38%	Unknown (RuBisCO activase 1)	52.0/6.28	49.5/5.42	gi|118489105<br>*Populus trichocarpa x Populus deltoides*	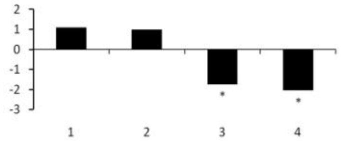
**212_III**<br>**(142_II)**	22	44%	Unknown (RuBisCO activase)	52.1/6.28	49.5/5.33	gi|118487547<br>*Populus trichocarpa*	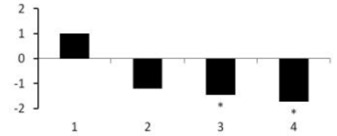
**215_III**	7	16%	Predicted protein (Sedo-heptulose-1,7-bisphospha-tase, chloroplast, putative)	42.4/5.77	47.4/4.96	gi|224112589<br>*Populus trichocarpa*	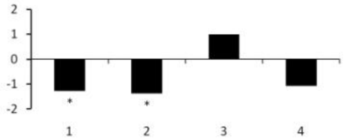
**216_III**<br>**(146_II)**	15	38%	Predicted protein (Phospho-glycerate kinase)	50.2/8.25	48.6/5.90	gi|224109060<br>*Populus trichocarpa*	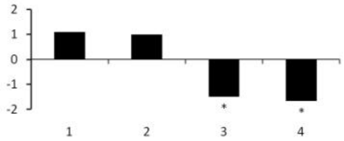
**223_III**	5	17%	Predicted protein (Phospho-ribulose kinase, putative)	45.0/6.11	47.7/5.50	gi|224138316<br>*Populus trichocarpa*	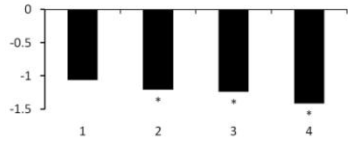
**227_III**<br>**(155_II)**	13	38%	Predicted protein (Phospho-ribulose kinase, putative)	45.0/5.90	47.0/5.40	gi|224071429<br>*Populus trichocarpa*	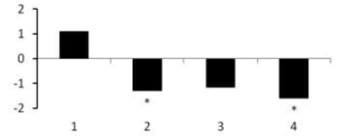

**236_III**	6	27%	Unknown (Alcohol dehydrogena-se, putative)	40.6/8.49	45.0/6.50	gi|118488941 *Populus trichocarpa x Populus deltoides*	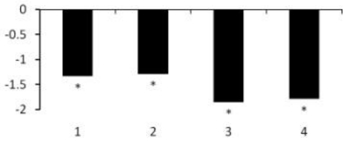
**238_III**	2	5%	Isovaleryl-CoA Dehydrogena-se; auxin binding protein (ABP44)	44.5/6.27	45.0/6.33	gi|5869965 *Pisum sativum*	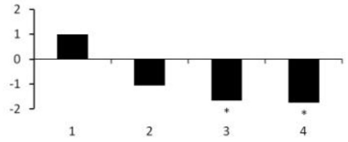
**241_III**	2	5%	Hypothetical protein (Aldo/keto reductase, putative)	40.5/ 6.69	45.0/6.70	gi|225446767 *Vitis vinifera*	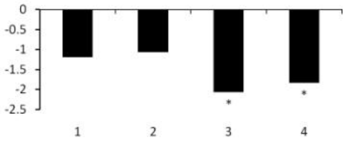
**244_III**	3	11%	Predicted protein (Pyruvate dehydrogenase (acetyl-transferring))	38.6/5.87	44.0/5.47	gi|224053535 *Populus trichocarpa*	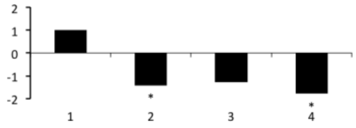
**247_III**	14	46%	Unknown (Alcohol dehydrogena-se, putative)	40.6/8.49	44.9/6.40	gi|118488941 *Populus trichocarpa x Populus deltoides*	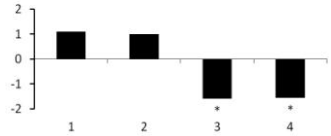
**261_III**	9	22%	Predicted protein	38.4/5.87	41.9/5.54	gi|224073126 *Populus trichocarpa*	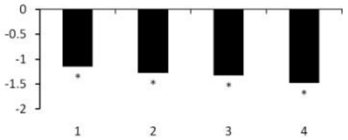
**270_III**	3	6%	RuBisCO large subunit	52.0/6.10	38.1/6.60	gi|1293020 *Polyscias guilfoylei*	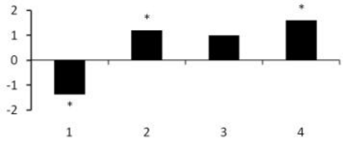
**277_III**	3	4%	RuBisCO large subunit	49.5/6.60	37.9/6.40	gi|46326306 *Salvia chamaedryoid es*	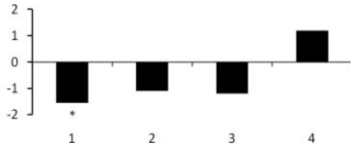

**279_III**	2	5%	RuBisCO large subunit	48.6/6.80	37.9/6.50	gi|14585745 *Veronica arguta*	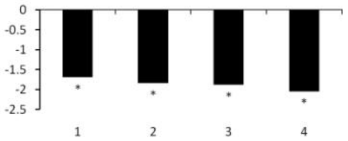
**286_III**	3	13%	Predicted protein	30.2/5.36	35.1/5.24	gi|224110036 *Populus trichocarpa*	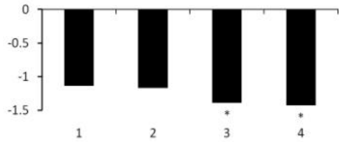
**289_III**	2	10%	Chain A, Profilin I	14.1/4.70	37.9/6.31	gi|157836856 *Arabidopsis thaliana*	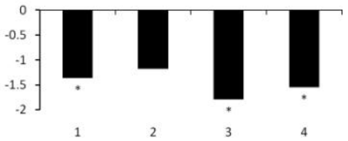
**290_III**	2	7%	Predicted protein (Ferredoxin--NADP reductase, putative)	40.4/8.71	37.9/6.70	gi|224074257 *Populus trichocarpa*	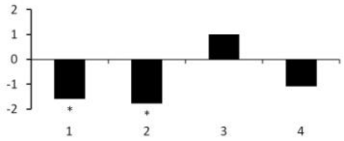
**293_III**	3	13%	Predicted protein (2-deoxyglucose-6-phosphate phosphatase, putative)	28.9/5.12	35.1/5.00	gi|224093744 *Populus trichocarpa*	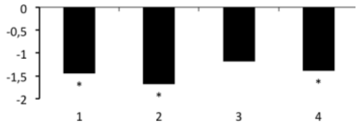
**295_III**	4	12%	Predicted protein (Plastid-specific 30S ribosomal protein 1)	34.1/6.78	37.9/6.50	gi|224118512 *Populus trichocarpa*	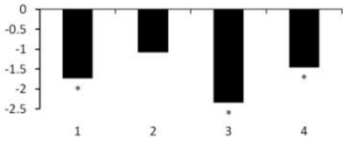
**301_III** **(314_I)** **(245_II)**	3	23%	Predicted protein (NAD-dependent epimerase / dehydratase)	27.0/5.68	35.1/5.45	gi|224090705 *Populus trichocarpa*	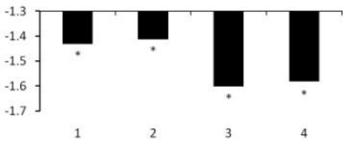
**305_III**	4	17%	Unknown	33.4/6.97	34.8/6.10	gi|118484329 *Populus trichocarpa*	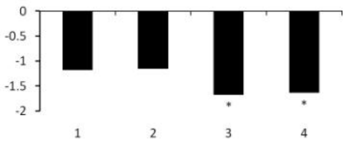
**308_III**	3	20%	Predicted protein (3-hydroxyiso-butyrate dehydrogena-	30.6/6.45	34.8/6.50	gi|224129290 *Populus trichocarpa*	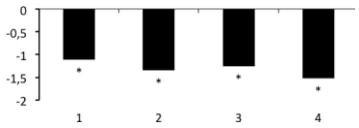

			se, putative)				
**310_III**	6	29%	Predicted protein (Cytosolic ascorbate peroxidase 1)	27.3/5.53	34.8/5.68	gi|224104631 *Populus trichocarpa*	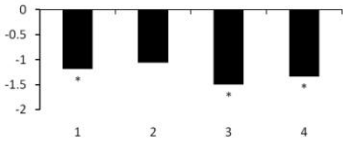
**313_III**	11	56%	Predicted protein (NAD-dependent epimerase / dehydratase)	27.0/5.68	34.8/5.57	gi|224090705 *Populus trichocarpa*	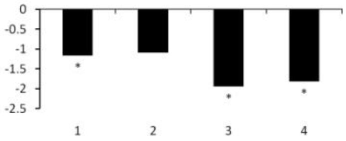
**314_III**	8	39%	Predicted protein (NAD-dependent epimerase / dehydratase)	27.0/ 5.68	33.5/5.35	gi|224090705 *Populus trichocarpa*	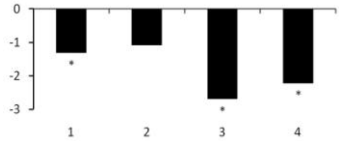
**315_III**	2	10%	Unknown (ATP synthase subunit mitochondrial)	27.8/8.50	34.8/6.33	gi|118484162 *Populus trichocarpa*	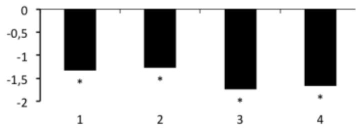
**317_III**	4	21%	Predicted protein (Carboxy-methylenebutenolidase, putative)	26.2/5.24	32.0/5.45	gi|224131618 *Populus trichocarpa*	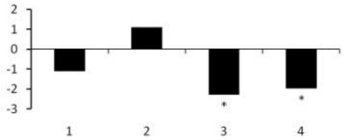
**319_III** **(253_II)**	5	18%	Predicted protein (Groes chaperonin, putative)	27.1/7.77	32.0/5.22	gi|224141565 *Populus trichocarpa*	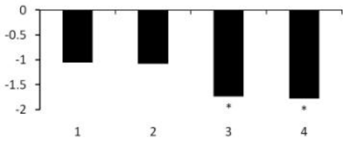
**320_III**	2	10%	Predicted protein (Chloroplast drought-induced stress protein, putative)	26.3/5.94	34.8/6.70	gi|224085954 *Populus trichocarpa*	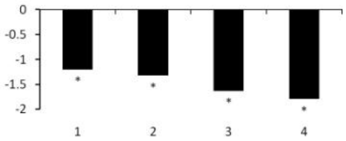
**332_III**	5	23%	Predicted protein (Chloroplast ferritin 2 precursor)	29.4/ 5.72	31.8/5.57	gi|224109256 *Populus trichocarpa*	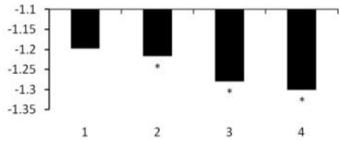

**333_III**	5	34%	Predicted protein (Phi class glutathione transferase GSTF2)	24.6/5.52	31.8/5.35	gi|224065729 *Populus trichocarpa*	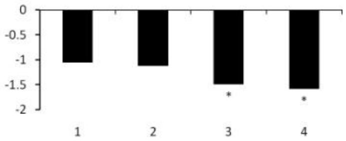
**334_III**	10	66%	Predicted protein (Glutathione-s-transferase theta)	24.6/5.52	31.8/5.60	gi|224065729 *Populus trichocarpa*	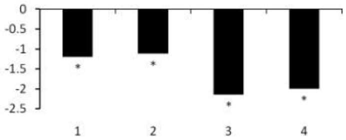
**346_III**	6	42%	Unknown (Light-harvesting complex I protein Lhca3)	29.6/9.10	29.6/6.00	gi|118489937 *Populus trichocarpa x Populus deltoides*	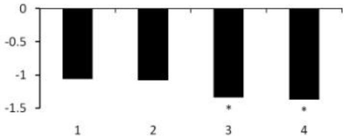
**361_III**	3	16%	Predicted protein (Heat shock protein, putative)	26.2/6.92	26.2/5.80	gi|224120952 *Populus trichocarpa*	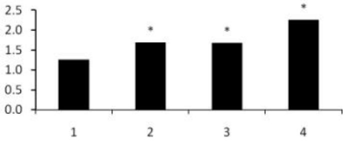
**363_III**	2	9%	Predicted protein (Heat shock protein, putative)	26.2/6.92	26.0/5.57	gi|224120952 *Populus trichocarpa*	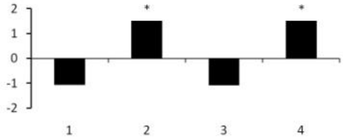
**384_III**	7	11%	RuBisCO	49.9/6.57	25.0/5.68	gi|6513629 *Ascarina* sp. Qiu-M149	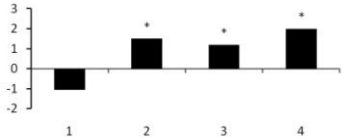
**394_III**	4	7%	RuBisCO large subunit	51.1/6.33	23.5/5.35	gi|493246 *Disporum sessile*	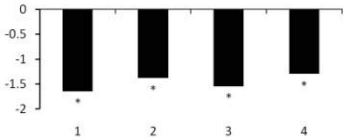
**487_III**	8	39%	Predicted protein (Thylakoid lumenal 15 kDa protein, Chloroplast)	23.4/6.82	20.0/5.17	gi|224098455 *Populus trichocarpa*	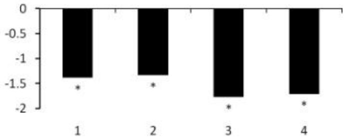

**594_III**	32	50%	RuBisCO large subunit	52.7/5.91	62.0/6.29	gi|110227087<br>*Populus alba*	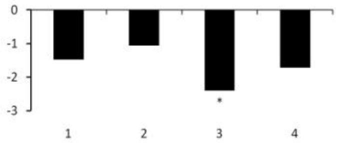
**598_III**<br>**(414_II)**	11	53%	Predicted protein (Cytosolic ascorbate peroxidase 1)	27.3/5.53	34.8/5.80	gi|224104631<br>*Populus trichocarpa*	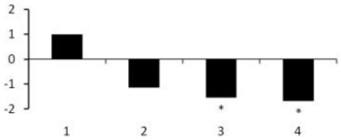
**600_III**	2	7%	Predicted protein	27.8/8.50	34.8/5.72	gi|224093896<br>*Populus trichocarpa*	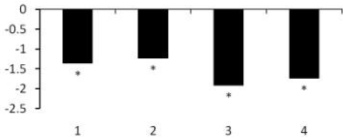
**601_III**	12	41%	Unknown (Groes chaperonin, putative)	26.8/8.76	31.8/5.72	gi|118489858<br>*Populus trichocarpa x Populus deltoids*	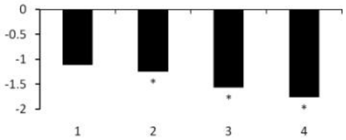
**602_III**	5	14%	Unknown (2-deoxyglucose-6-phosphate phosphatase, putative)	35.2/8.00	37.9/5.33	gi|118488927<br>*Populus trichocarpa x Populus deltoides*	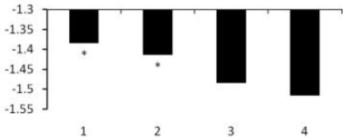
**603_III**<br>**(409_II)**	8	38%	Unknown (Photosystem II oxygen-evolving complex 33KDa subunit)	35.1/5.62	37.7/5.33	gi|118489901<br>*Populus trichocarpa x Populus deltoides*	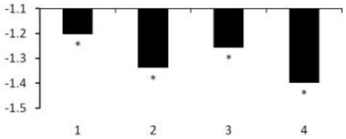
**610_III**<br>**(411_II)**	5	23%	Unknown (Photosystem II oxygen-evolving complex 33 KDa subunit)	35.1/5.62	35.1/5.17	gi|118489901<br>*Populus trichocarpa x Populus deltoides*	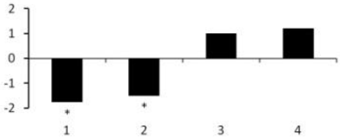
**611_II**	6	45%	Putative protein (Oxygen-evolving enhancer protein 1, chloroplast precursor, putative)	18.5/5.17	35.0/5.17	gi|190898996<br>*Populus tremula*	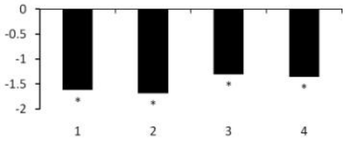

**613_III**<br>**(247_I)**<br>**(174_II)**	6	25%	Unknown (Fructose-bisphosphate aldolase, putative)	42.9/8.17	44.9/6.29	gi|118489355<br>*Populus trichocarpa x Populus deltoides*	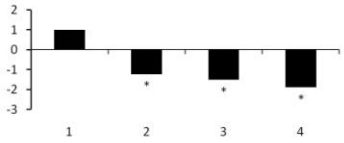
**614_III**	10	47%	Predicted protein (DHAR class glutathione transferase DHAR1)	24.3/4.93	34.8/4.93	gi|224065178<br>*Populus trichocarpa*	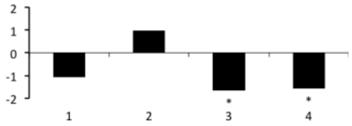

a) In brackets, corresponding spot number in the other samplings (manually checked and confirmed by MS/MS analysis).

b) Number of identified peptides and sequence coverage.

c) Graphical representation of the average ratios of the protein abundance: PolI/C (1), Gi/C (2), GiPolI/Gi (3), GiPolI/PolI (4). Positive values are given as such, whereas negative values are given according to the following formula: given value =-1/ratio. Value exceeding ± 2 are indicative of strong protein induction and reduction, respectively. The presence of asterisk is indicative of a statistically significant average ratio.
