# Supplementary material for: Effects of Heavy Metals and Arbuscular Mycorrhiza on the Leaf Proteome of a Selected Poplar Clone: A Time Course Analysis
Source: PLoS One. 2012 Jun 26;7(6):e38662. doi: 10.1371/journal.pone.0038662 (PMC3383689; doi:10.1371/journal.pone.0038662)
Supplement: Table S4 — OD Values - first sampling (S1). List of the spots showing significantly different average optical densities (± standard errors) and relative P values. Different letters indicate statistically significant differences (P<0.05). (PDF) [file pone.0038662.s005.pdf]

Table S4. OD Values - first sampling (S1). List of the spots showing significantly different average optical densities (± standard errors) and relative P values. Different letters indicate statistically significant differences (P<0.05).

Spot	Control	Polluted	G. intraradices	G. intraradices+polluted	P value
**104**	217369.921 ± 44535.834 a	358246.505 ± 30618.091 b	141062.027 ± 27059.527 a	213825.662 ± 40174.875 a	0.0079
**112**	179596.256 ± 13596.094 a	173780.830 ± 14117.244 a	100481.925 ± 6180.608 b	154447.677 ± 24862.829 ab	0.0385
**124**	186646.874 ± 25381.776 ac	221127.216 ± 18736.704 a	113526.125 ± 19503.639 b	144943.657 ± 7511.881 bc	0.0067
**130**	1010613.123 ± 348938.089 ac	1259160.280 ± 172781.477 a	307737.101 ± 43395.786 b	577510.949 ± 57528.722 bc	0.0349
**153**	515420.788 ± 29626.958 a	433549.914 ± 52704.816 ac	288760.292 ± 38902.470 bc	343197.076 ± 38800.726 c	0.0081
**154**	1502775.061 ± 177282.222 a	1439107.000 ± 83160.720 a	937132.335 ± 100108.653 b	1096031.931 ± 63011.535 b	0.0121
**165**	5896568.483 ± 852493.135 a	5486384.146 ± 650223.189 a	2791271.769 ± 308267.165 b	4676283.653 ± 713156.205 ab	0.0488
**230**	95583.820 ± 8997.524 a	70586.018 ± 7405.287 b	107476.534 ± 4419.226 a	101193.957 ± 5238.917 a	0.0106
**247**	806427.218 ± 66997.817 ac	759791.774 ± 100202.768 a	1343770.149 ± 62826.960 b	1068828.129 ± 125054.073 bc	0.0034
**283**	146778.636 ± 11446.961 a	170703.202 ± 10783.799 a	232546.793 ± 17974.662 b	145786.272 ± 10460.011 a	0.0007
**304**	533206.004 ± 57313.543 a	448731.951 ± 68706.738 a	842888.992 ± 131946.645 b	597632.390 ± 14249.029 a	0.0092
**314**	132314.032 ± 14266.014 ac	98910.731 ± 9077.006 a	240142.720 ± 39864.834 b	184264.151 ± 29405.157 bc	0.0042
**366**	708417.596 ± 102977.820 a	387956.966 ± 16447.484 b	311870.115 ± 23781.042 b	271075.766 ± 8371.555 b	0.0001
**397**	1170411.640 ± 92916.021 ac	668130.492 ± 176638.824 a	2101381.813 ± 333599.829 b	1336224.900 ± 218728.990 c	0.0017
**445**	735071.633 ± 145231.336 a	353464.958 ± 32826.947 a	1475189.794 ± 502586.795 b	730402.494 ± 97536.753 a	0.0148
**470**	1549248.760 ± 139854.922 a	1651400.128 ± 128392.010 a	868944.543 ± 61173.801 b	474690.467 ± 47116.468 c	< 0.0001
**471**	337642.230 ± 34524.533 a	348523.858 ± 33550.718 a	191482.293 ± 16098.106 b	153209.432 ± 19546.731 b	0.0001
**484**	60881.799 ± 8751.576 a	62081.865 ± 8101.671 a	27693.004 ± 1668.564 b	47382.895 ± 6699.255 a	0.0287
**485**	217369.921 ± 44535.834 a	358246.505 ± 30618.091 b	141062.027 ± 27059.527 a	213825.662 ± 40147.875 a	0.0079
**489**	1844281.643 ± 132460.588 a	1226964.680 ± 21958.315 b	1456144.458 ± 146172.281 b	1330220.579 ± 67778.318 b	0.0010
**491**	232007.723 ± 20912.535 a	304894.130 ± 39238.841 b	174372.858 ± 11293.869 c	207362.934 ± 13341.204 c	0.0171
**494**	346097.416 ± 25876.553 a	349798.144 ± 21370.964 a	155842.239 ± 19191.162 b	229492.740 ± 26216.067 b	< 0.0001
