# Supplementary material for: Effects of Heavy Metals and Arbuscular Mycorrhiza on the Leaf Proteome of a Selected Poplar Clone: A Time Course Analysis
Source: PLoS One. 2012 Jun 26;7(6):e38662. doi: 10.1371/journal.pone.0038662 (PMC3383689; doi:10.1371/journal.pone.0038662)
Supplement: Table S5 — OD Values - second sampling (S2). List of the spots showing significantly different average optical densities (± standard errors) and relative P values. Different letters indicate statistically significant differences (P<0.05). (PDF) [file pone.0038662.s006.pdf]

Table S5. OD Values - second sampling (S2). List of the spots showing significantly different average optical densities (± standard errors) and relative P values. Different letters indicate statistically significant differences (P<0.05).

Spot	Control	Polluted	G. intraradices	G. intraradices+polluted	P value
**57**	105855.011 ± 22189.264 a	159642.994 ± 60451.928 ab	99215.784 ± 6433.463 a	223710.010 ± 17552.131 b	0,0024
**118**	674239.613 ± 48048.595 ac	211986.413 ± 33192.265 b	868154.314 ± 169359.267 a	409949.541 ± 45852.219 bc	0,0039
**119**	408856.034 ± 108390.473 a	104291.868 ± 18037.453 b	386650.295 ± 67646.678 a	159817.082 ± 17201.883 b	0,0180
**122**	106527.674 ± 8283.670 a	68648.493 ± 9433.677 a	153997.661 ± 15622.486 b	98326.832 ± 12144.045 a	0,0016
**132**	143432.792 ± 6724.302 a	95796.638 ± 11843.291 b	126462.439 ± 4490.640 ab	114934.509 ± 5460.719 b	0,0009
**134**	486007.129 ± 34508.653 a	179180.781 ± 26659.775 a	781572.717 ± 156147.585 b	389036.045 ± 40683.300 a	0,0054
**135**	182559.191 ± 13328.737 ab	58106.704 ± 2528.324 a	281553.557 ± 74104.419 b	142488.112 ± 12431.216 a	0,0395
**137**	187040.366 ± 16001.277 a	82801.423 ± 5663.967 b	242327.824 ± 25792.292 c	116481.831 ± 5176.951 b	< 0.0001
**142**	875789.596 ± 104912.382 ac	298819.547 ± 18279.372 b	1148282.879 ± 211566.695 a	519087.199 ± 24872.744 bc	0,0031
**146**	336328.936 ± 17874.232 a	234512.412 ± 20444.871 a	457724.899 ± 58039.621 b	305112.903 ± 9444.996 a	0,0051
**148**	301454.534 ± 26165.369 a	144887.665 ± 20799.015 b	276643.958 ± 23994.775 a	238229.067 ± 12630.597 a	0,0018
**149**	375650.630 ± 59596.035 a	94081.903 ± 13206.485 b	271758.958 ± 31339.830 a	264702.473 ± 52781.782 a	0,0122
**150**	186678.385 ± 22968.326 a	120303.928 ± 5251.835 a	248009.585 ± 31168.604 b	160528.960 ± 13258.764 a	0,0062
**152**	130845.133 ± 10052.296 ac	81813.318 ± 13419.814 b	156225.736 ± 9542.298 a	112635.069 ± 5535.447 bc	0,0003
**155**	289936.873 ± 29504.381 ac	126345.261 ± 14781.441 b	382051.458 ± 56660.712 a	253760.480 ± 24027.808 bc	0,0056
**161**	191322.120 ± 9778.754 a	119965.043 ± 21538.449 b	256408.940 ± 13466.280 c	166444.600 ± 16188.471 ab	< 0.0001
**162**	133166.678 ± 6408.351 a	53862.725 ± 4398.719 a	231013.554 ± 38062.849 b	97639.952 ± 2610.940 a	0,0003
**163**	154538.700 ± 12321.532 a	87191.576 ± 8600.242 a	262449.312 ± 46590.376 b	132537.586 ± 10782.745 a	0,0042
**164**	108239.799 ± 7402.900 a	55786.163 ± 5383.249 b	146434.787 ± 19108.406 c	77005.867 ± 5099.379 b	0,0005
**165**	527478.407 ± 72768.098 a	308200.267 ± 30245.726 a	854709.112 ± 148424.117 b	487803.906 ± 17173.623 a	0,0098
**166**	131376.561 ± 14877.301 a	115178.534 ± 6100.048 a	233051.314 ± 25109.121 b	109127.156 ± 14849.281 a	0,0002
**171**	59119.407 ± 1921.524 a	36406.865 ± 2594.558 b	84136. 363 ± 7615.554 c	53792.882 ± 3214.166 a	< 0.0001
**172**	265107.341 ± 16363.188 ac	169066.195 ± 8805.212 b	313115.066 ± 32815.156 a	211861.199 ± 9469.176 bc	0,0022
**174**	504584.091 ± 20140.335 a	279910.547 ± 12233.430 a	697184.016 ± 108752.154 b	441346.731 ± 24748.068 a	0,0059
**181**	456369.823 ± 41436.111 ac	270490.669 ± 19184.784 b	556753.873 ± 70253.576 a	381470.390 ± 25564.157 c	0,0100
**193**	128275.274 ± 4029.924 ab	111783.072 ± 40981.634 ab	157563.023 ± 17548.263 a	82879.903 ± 3448.792 b	0,0189

**202**	327907.728 ± 15955.869 a	152469.563 ± 20924.179 b	192861.984 ± 6522.641 bc	233223.520 ± 17527.492 c	< 0.0001
**214**	79099.056 ± 7112.637 a	148172.494 ± 8453.804 b	85748.081 ± 2830.057 a	47097.434 ± 6708.658 c	< 0.0001
**241**	248722.058 ± 8516.653 a	146901.041 ± 11387.556 b	254772.921 ± 31105.072 a	230890.634 ± 9649.699 a	0,0189
**245**	212613.868 ± 15889.850 a	154244.052 ± 14023.473 ab	191208.324 ± 22466.197 a	124154.251 ± 13563.967 b	0,0105
**246**	176603.635 ± 14751.627 a	86237.873 ± 7831.449 b	150911.934 ± 20102.043 a	80120.504 ± 10050.196 b	0,0005
**253**	210590.501 ± 14683.177 a	236033.616 ± 14554.218 a	310962.893 ± 22316.769 b	190692.613 ± 23025.001 a	0,0012
**254**	319126.200 ± 18138.543 ac	131704.667 ± 12525.100 b	437634.246 ± 87472.652 a	200246.044 ± 9851.704 bc	0,0056
**255**	303433.124 ± 28680.409 a	864809.501 ± 126183.535 b	225690.977 ± 45097.210 a	449107.710 ± 59348.836 a	< 0.0001
**269**	742431.397 ± 162571.276 ab	961521.693 ± 51625.136 a	424312.095 ± 22682.262 c	609165.746 ± 71561.368 bc	0,0123
**272**	916561.376 ± 55538.781 a	513900.756 ± 52176.053 b	687676.043 ± 39400.305 c	737804.614 ± 23250.268 c	< 0.0001
**275**	75697.326 ± 8404.618 a	44528.791 ± 5601.018 b	107284.911 ± 9306.157 c	65469.843 ± 5510.474 b	0,0003
**291**	509291.840 ± 37573.612 a	211888.097 ± 49467.716 b	412983.416 ± 25549.483 c	402075.496 ± 30554.237 c	0,0002
**294**	242006.719 ± 18254.499 a	165553.383 ± 5687.895 b	246910.790 ± 22305.470 a	156359.010 ± 17883.976 b	0,0036
**303**	691644.607 ± 105300.380 a	933848.564 ± 117298.427 a	377865.613 ± 50218.082 b	724233.842 ± 52694.796 a	0,0009
**312**	106912.660 ± 8347.738 a	230042.309 ± 63915.148 b	85431.328 ± 11268.078 a	326723.456 ± 50496.272 b	< 0.0001
**402**	182066.327 ± 8378.098 a	70183.843 ± 11609.059 b	101484.961 ± 4191.622 bc	115856.277 ± 17850.796 c	< 0.0001
**403**	304388.699 ± 41520.963 a	124876.101 ± 7846.492 b	203993.593 ± 16298.374 b	182884.774 ± 15976.139 b	0,0016
**409**	885715.329 ± 97648.961 a	340973.503 ± 56104.574 b	628338.364 ± 35767.412 c	454900.974 ± 22679.905 b	< 0.0001
**410**	445786.720 ± 70572.905 a	181308.463 ± 20815.096 b	240985.954 ± 20856.707 b	263732.091 ± 48486.041 b	0,0070
**411**	716434.056 ± 88589254 a	289789.075 ± 53220.961 b	549344.418 ± 29103.423 c	428556.085 ± 36616.421 bc	0,0006
**414**	471326.465 ± 32027.867 ac	208368.705 ± 25223.485 b	518470.108 ± 91909.642 a	309926.697 ± 15410.823 bc	0,0099
**415**	381771.824 ± 29701.540 ac	200534.077 ± 16397.487 b	411134.880 ± 75560.426 a	246955.082 ± 13549.526 bc	0,0265
**419**	466792.456 ± 24468.454 a	301084.291 ± 50106.435 b	502673.081 ± 39793.048 a	368029.680 ± 28706.347ab	0,0040
**420**	246858.913 ± 13707.556 a	155215.596 ± 4785.483 b	294592.178 ± 34129.448 a	194833.509 ± 4179.758 ab	0,0025
**421**	232146.064 ± 10546.454 a	148582.266 ± 45484.873 a	352870.227 ± 59780.911 b	153566.405 ± 9327.260 a	0,0041
**423**	216668.062 ± 32676.549 a	102379.226 ± 7107.060 b	119030.787 ± 6850.864 b	137955.588 ± 27145.903 b	0,0129
