# Supplementary material for: Effects of Heavy Metals and Arbuscular Mycorrhiza on the Leaf Proteome of a Selected Poplar Clone: A Time Course Analysis
Source: PLoS One. 2012 Jun 26;7(6):e38662. doi: 10.1371/journal.pone.0038662 (PMC3383689; doi:10.1371/journal.pone.0038662)
Supplement: Table S6 — OD Values - third sampling (S3). List of the spots showing significantly different average optical densities (± standard errors) and relative P values. Different letters indicate statistically significant differences (P<0.05). (PDF) [file pone.0038662.s007.pdf]

Table S6. OD Values - third sampling (S3). List of the spots showing significantly different average optical densities (± standard errors) and relative P values. Different letters indicate statistically significant differences (P<0.05).

Spot	Control	Polluted	G. intraradices	G. intraradices+polluted	P value
85	61582.777 ± 13264.173 a	71426.979 ± 9366.759 a	76859.007 ± 11418.340 a	135182.493 ± 11103.874 b	0,0013
105	167267.807 ± 28574.825 a	117528.560 ± 5963277 ac	222329.445 ± 15749.682 b	95561.947 ± 3494.221 c	0,0003
118	2292302.389 ± 234695.128 a	1968785.627 ± 128475.784 a	1564717.214 ± 44688.334 b	1246710.406 ± 83102.274 b	0,0005
132	235387.605 ± 26053.527 a	440556.899 ± 61560.622 b	263358.453 ± 22876.748 a	292936.787 ± 17291.522 a	0,0050
171	944398.653 ± 125544.114 a	1867357.815 ± 282668.472 b	798339.132 ± 44618.326 a	745073.527 ± 35105.133 a	0,0003
176	1882616.111 ± 151202.052 a	1643522.365 ± 160459.038 a	1587412.260 ± 69202.915 a	1056314.315 ± 54175.882 b	0,0012
178	739962.999 ± 50076.183 a	777540.916 ± 34373.149 a	604059.269 ± 39361.162 b	457158.869 ± 20082.517 c	< 0.0001
197	190893.705 ± 35726.445 a	180058.338 ± 25569.029 a	198150.019 ± 10992.998 a	93660.055 ± 4695.655 b	0,0172
199	1975972.572 ± 148659.445 a	1632734.307 ± 154265.927 b	1402183.546 ± 43284.264 b	788383.812 ± 37097.108 c	< 0.0001
200	351650.026 ± 27542.509 a	377977.867 ± 33667.165 a	306858.341 ± 30197.183 a	140175.158 ± 11782.775 b	< 0.0001
204	749086.669 ± 40945.229 a	613655.634 ± 39731.353 b	565126.064 ± 54077.417 b	368976.817 ± 20111.506 c	< 0.0001
209	801065.074 ± 65053.168 a	916689.645 ± 43074.022 a	778914.162 ± 38779.207a	448173.708 ± 30837.218 b	< 0.0001
212	2089512.953 ± 103693.783 a	2128508.188 ± 109714.296 a	1875889.108 ± 132232.860 a	1228273.173 ± 130549.411 b	0,0002
215	322535.050 ± 19913.266 a	251138.392 ± 19184.809 b	232709.998 ± 13406.897 b	231936.819 ± 12102.648 b	0,0038
216	599785.165 ± 21149.367 a	642824.830 ± 34442.873 a	577330.035 ± 10240.950 a	387101.389 ± 9656.231 b	< 0.0001
223	953807.397 ± 40458.148 a	898726.308 ± 54143.252 a	788704.957 ± 20644.456 b	635439.028 ± 35077.920 c	0,0002
227	649228.977 ± 37194.168 a	680619.207 ± 36689.127 a	495399.364 ± 19436.336 b	422263.920 ± 19382.786 b	< 0.0001
236	347244.076 ± 10979.478 a	259746.115 ± 23273.286 b	269137.591 ± 26774.310 b	145071.301 ± 12327.491 c	< 0.0001
238	698161.139 ± 20197.456 a	697191.337 ± 47747.155 a	662572.442 ± 36377.368 a	397698.672 ± 27356.470 b	< 0.0001
241	354196.067 ± 19566.408 a	295689.122 ± 33237.102 a	333074.199 ± 13836.297 a	161020.015 ± 9237.912 b	< 0.0001
244	101972.017 ± 10195.874 a	101004.730 ± 3569.314 a	71690.816 ± 4831.650 b	56483.645 ± 4156.960 b	0,0002
247	1018686.819 ± 34655.748 a	993890.010 ± 77109.555 a	1016375.486 ± 38377.224 a	635280.243 ± 32441.188 b	< 0.0001
261	260329.462 ± 8531.420 a	226901.176 ± 13691.974 b	204136.511 ± 7855.058 b	153818.165 ± 11418.281 c	< 0.0001
270	367961.391 ± 15899.795 a	268474.027 ± 31970.284 b	445648.672 ± 24118.577 c	431105.149 ± 13905.256 ac	0,0002
277	449345.064 ± 33692.755 a	288842.277 ± 18305.091 b	408774.890 ± 33070.449 ac	342222.189 ± 19954.764 bc	0,0036
279	762299.059 ± 51468.150 a	450048.858 ± 29995.082 b	414142.833 ± 25503.752 b	219687.013 ± 23299.653 c	< 0.0001

**286**	727989.010 ± 70284.669 a	642123.349 ± 43589.743 a	624996.244 ± 55935.355 a	450012.209 ± 46199.410 b	0,0181
**289**	358981.664 ± 11633.603 a	262666.609 ± 25696.346 b	303723.047 ± 14894.135 ab	169223.980 ± 21186.449 c	< 0.0001
**290**	508176.674 ± 28767.722 a	319273.487 ± 30341.702 b	286398.734 ± 23080.031 b	295511.661 ± 7445.473 b	< 0.0001
**293**	797244.541 ± 51602.555 a	552116.925 ± 44387.514 b	472779.520 ± 37100.721 bc	397980.096 ± 17800.090 c	< 0.0001
**295**	107414.216 ± 8826.524 a	61814.762 ± 4484.362 b	99736.176 ± 5823.675 a	42483.787 ± 2612.481 c	< 0.0001
**299**	176833.347 ± 16686.194 a	166166.901 ± 11016.546 a	173988.666 ± 4593.082 a	95199.040 ± 11975.526 b	0,0004
**301**	211489.433 ± 11455.465 a	147728.379 ± 5865.292 b	149662.907 ± 5628.044 b	93424.138 ± 4132.238 c	< 0.0001
**305**	728822.948 ± 54641.500 a	616461.151 ± 55096.047 a	630701.133 ± 78045.225 a	376530.673 ± 19369.992 b	0,0029
**308**	342598.528 ± 6497.242 a	308658.450 ± 5483.380 b	254994.090 ± 10485.886 c	203731.554 ± 10305.720 d	< 0.0001
**310**	353336.371 ± 16769.511 a	297646.564 ± 13626.562 b	334192.392 ± 13936.755 ab	222871.258 ± 11622.781 c	< 0.0001
**313**	720498.714 ± 36399.835 a	617497.510 ± 26412.227 b	659097.593 ± 35078.869 ab	338918.366 ± 17412.312 c	< 0.0001
**314**	284045.880 ± 21669.462 a	215683.831 ± 18476.301 b	261816.014 ± 14279.275 a	97274.486 ± 5757.782 c	< 0.0001
**315**	363251.937 ± 27544.930 a	272589.683 ± 39722.167 b	285545.787 ± 13895.762 b	163810.493 ± 4277.884 c	0,0004
**317**	394441.287 ± 39901.466 a	358126.757 ± 44175.258 a	415527.791 ± 20787.172 a	182017.487 ± 11957.978 b	0,0004
**319**	476271.553 ± 16864.332 a	451171.924 ± 37285.143 a	440564.795 ± 41772.743 a	254219.563 ± 9734.171 b	0,0002
**320**	301508.895 ± 20257.560 a	251024.141 ± 11570.440 b	227917.040 ± 19932.326 b	139725.471 ± 6725.671 c	< 0.0001
**329**	4675.584 ± 477.845 a	13872.116 ± 4394.433 a	4424.431 ± 369.084 a	56562.706 ± 5603.538 b	< 0.0001
**332**	224706.455 ± 6504.366 a	187624.159 ± 20930.659 ab	184623.819 ± 12731.831 b	144257.506 ± 5603.794 c	0,0046
**333**	770264.462 ± 23725.001 a	729200.282 ± 91399.487 a	686508.843 ± 29388.500 a	459193.103 ± 25195.719 b	0,0025
**334**	1004742.792 ± 34800.873 a	838474.765 ± 32143.646 b	899895.213 ± 72832.137 b	419569.527 ± 23653.309 c	< 0.0001
**346**	1611776.572 ± 98822.544 a	1517626.549 ± 125903.870 a	1487421.066 ± 33946.272 a	1109120.771 ± 44225.110 b	0,0036
**361**	268562.389 ± 13616.132 a	337891.714 ± 57061.042 a	454117.705 ± 16516.774 b	763214.417 ± 33884.397 c	< 0.0001
**363**	121885.596 ± 7186.127 a	113623.631 ± 22185.998 a	182568.603 ± 4090.558 b	167497.219 ± 4573.988 b	0,0019
**384**	294997.779 ± 8711.249 a	275939.058 ± 44288.785 a	456152.916 ± 16190.795 b	546334.352 ± 21843.757 c	< 0.0001
**394**	508542.945 ± 18526.525 a	308866.642 ± 46213.495 b	367974.477 ± 16392.540 b	238502.700 ± 14230.582 c	< 0.0001
**471**	339014.787 ± 115648.707 a	167151.686 ± 11317.575 b	164975.107 ± 28561.023 b	114129.976 ± 10055.738 b	0,0478
**487**	136602.695 ± 10663.099 a	98690.062 ± 13173.502 b	102298.873 ± 13454.638 b	57742.156 ± 3812.903 c	0,0012
**594**	6756208.367 ± 1375588.158 a	4557463.974 ± 654900.409 ab	6348139.359 ± 374268.137 a	2653098.517 ± 519964.548 b	0,0110
**598**	1116248.602 ± 64296.590 a	1065560.409 ± 61627.678 a	981904.478 ± 16612.247 a	632697.923 ± 36400.434 b	< 0.0001
**599**	415167.937 ± 62389.025 a	401360.628 ± 65357.812 a	359630.853 ± 18116.163 a	205373.154 ± 16626.706 b	0,0222

**600**	413796.254 ± 9020.143 a	303114.506 ± 13940.022 b	334578.787 ± 10580.851 b	174185.601 ± 21045.586 c	< 0.0001
**601**	787332.400 ± 41876.440 a	708453.078 ± 75367.759 ab	630162.265 ± 20816.444 b	401625.380 ± 12159.629 c	0,0001
**602**	513668.456 ± 84849.860 a	370902.069 ± 33772.328 b	363145.984 ± 16769.230 b	244598.148 ± 9890.436 b	0,0083
**603**	1587397.656 ± 53937.533 a	1319617.308 ± 48028.651 b	1186316.246 ± 59206.988 b	944168.177 ± 21049.372 c	< 0.0001
**608**	152969.159 ± 5606.542 a	306620.759 ± 37582.260 b	116277.945 ± 13363.179 a	490476.964 ± 54203.329 c	< 0.0001
**609**	133173.261 ± 10747.137 a	277117.731 ± 29570.306 b	88597.512 ± 8188.184 a	223242.423 ± 19882.625 c	< 0.0001
**610**	577407.096 ± 83050.195 a	327288.761 ± 21612.960 b	384401.900 ± 12834.622 b	385727.155 ± 17277.477 b	0,0057
**611**	895942.858 ± 38136.845 a	552489.901 ± 25946.713 b	531887.732 ± 35993.350 b	408368.732 ± 11414.423 c	< 0.0001
**613**	787849.533 ± 36978.971 a	796726.432 ± 66560.990 a	640699.981 ± 45297.893 b	422201.491 ± 36920.269 c	0,0001
**614**	788899.167 ± 76934.053 a	733302.882 ± 50075.083 a	775255.502 ± 29959.215 a	471062.350 ± 32298.097 b	0,0013
