# Supplementary material for: Effects of Heavy Metals and Arbuscular Mycorrhiza on the Leaf Proteome of a Selected Poplar Clone: A Time Course Analysis
Source: PLoS One. 2012 Jun 26;7(6):e38662. doi: 10.1371/journal.pone.0038662 (PMC3383689; doi:10.1371/journal.pone.0038662)
Supplement: Table S7 — Identification of poplar leaf proteins – first sampling (S1). Precursor ion m/z, calculated peptide mass, ion score, modification, protein name, theoretical molecular weight and pI, accession number and reference organism, and blast results for each identified spot. (PDF) [file pone.0038662.s008.pdf]

Table S7. Identification of poplar leaf proteins – first sampling (S1). Precursor ion m/z, calculated peptide mass, ion score, modification, protein name, theoretical molecular weight and pI, accession number and reference organism, and blast results for each identified spot.

Spot	Precursor ion *m/z*	Peptide mass calculated	Sequence	Ion Score	Modification	Protein name	M_r (kDa) / pI Theor	AC number (gi NCBI) and reference organism	Blast results
**104**	631.3687	1260.7078	DITLGFVDLLR	52	-------	Ribulose-1,5-bisphosphate carboxylase	52858/	gi|2961315/	-------
	729.3620	2185.0110	GGLDFTKDDENVNSKPFMR	11	Oxidation (M)		6.14	*Spigelia anthelmia*	
**112**	719.3605	1436.7300	VQQLLQDFFDGK	19	------	Heat shock protein 70	71444/	gi|6911551/	-------
	1011.9145	3032.7196	VQDLLLLDVTPLSLGLETAGGVMTVLIPR	65	------		5.07	*Cucumis sativus*	
**124**	504.2850	1006.5699	LSIFETGIK	53	------	ATP synthase beta subunit	51786/	gi|14718046/	-------
	639.3458	1276.6849	MPNIYNALVVK	71	Oxidation (M)		5.20	*Eucryphia lucida*	
	717.3869	1432.7674	FVQAGSEVSALLGR	93	-----				
	744.4279	1486.8680	TVLIMELIINIAK	35	Deamidated (NQ); Oxidation (M)				
	801.4105	1600.8031	VALVYGQMNEPPGAR	31	-------				
	1057.0399	2112.0885	GMEVIDTGAPLSVPVGGATLGR	22	Oxidation (M)				
**130**	489.7642	977.4971	FRAPVEPY.-	14	------	Predicted protein	47897/	gi|224136806/	Enolase
	754.3908	1506.7565	IEEELGSAAVYAGAK	30	------		5.67	*Populus trichocarpa*	
	787.4334	1572.8359	VNQIGSVTESIEAVK	13	------				
	895.9885	1789.9210	AAVPSGASTGVYEALELR	127	------				
**153**	488.2884	974.5549	IGLFGGAGVGK	52	------	ATP synthase beta subunit	53590/	gi|110227086/	-------
	504.2955	1006.5699	LSIFETGIK	34	------		5.09	*Populus alba*	
	523.3024	1044.5968	VVDLLAPYR	70	------				
	595.8187	1189.6343	SAPAFIQLDTK	47	------				
	639.3387	1276.6849	MPNIYNALVVK	42	Oxidation (M)				
	717.3743	1432.7674	FVQAGSEVSALLGR	89	------				
	728.7506	2183.2467	NLGHIAQIIGPVLDVVFPPGK	47	------				
	736.3803	1470.7541	VGLTALTMAEYFR	88	------				
	744.3998	1486.8429	TVLIMELINNIAK	70	Oxidation (M)				
	809.3945	1616.7981	VALVYGQMNEPPGAR	32	Oxidation (M)				
	883.8224	2648.3446	IFNVLGEPVDDLGPVDTGTTSPIHR	88	------				
	969.5054	2905.4960	LILSGELDSLPEQAFYLVGNIDEATAK	72	------				
	975.5003	1948.9894	DVNEQDVLLFIDNIFR	72	------				
	1057.0613	2112.0885	GMEVIDTGAPLSVPVGGATLGR	51	Oxidation (M)				
	1282.3350	3843.9055	EGSITSIQAVYVPADDLTDPAPATTFAHLDATTVLSR	2	------				
**154**	488.2822	974.5549	IGLFGGAGVGK	42	------	ATP synthase beta subunit	53590/	gi|110227086/	--------
	504.2892	1006.5699	LSIFETGIK	43	------		5.09	*Populus alba*	
	523.3024	1044.5968	VVDLLAPYR	62	------				
	596.3127	1190.6183	SAPAFIQLDTK	51	Deamidated (NQ)				
	597.6380	1789.8855	MRVGLTALTMAEYFR	30	2 Oxidation (M)				
	639.3529	1276.6849	MPNIYNALVVK	75	Oxidation (M)				
	678.7258	2033.1561	LSIFETGIKVVDLLAPYR	67	------				
	717.3919	1432.7674	FVQAGSEVSALLGR	97	------				
	727.0698	2178.1936	SAPAFIQLDTKLSIFETGIK	43	------				
	728.7633	2183.2467	NLGHIAQIIGPVLDVVFPPGK	67	------				
	730.7626	2189.2572	LSIFETGIKVVDLLAPYRR	28	------				
	736.3803	1470.7541	VGLTALTMAEYFR	88	------				
	744.4228	1486.8429	TVLIMELINNIAK	80	Oxidation (M)				
	771.4565	2311.3416	KNLGHIAQIIGPVLDVVFPPGK	12	------				
	776.7086	2327.0773	MPSAVGYQPTLSTEMGTLQER	68	2 Oxidation (M)				
	801.4131	1600.8031	VALVYGQMNEPPGAR	70	------				
	821.4246	2461.2587	YKELQDIIAILGLDELSEEDR	37	------				
	861.5004	3441.9210	NLGHIAQIIGPVLDVVFPPGKMPNIYNALVVK	20	Oxidation (M)				
	883.7890	2648.3446	IFNVLGEPVDDLGPVDTGTTSPIHR	93	Deamidated (NQ)				
	921.0792	2760.2105	EGNDLYMEMKESGVINEENIAESK	24	2 Oxidation (M)				
	931.4776	3721.8484	MRVGLTALTMAEYFRDVNEQDVLLFIDNIFR	33	Deamidated (NQ); 2 Oxidation (M)				
	969.5112	2905.4960	LILSGELDSLPEQAFYLVGNIDEATAK	72	-------				
	1001.5466	3001.5971	YKELQDIIAILGLDELSEEDRLTVAR	43	------				
	1049.0688	2096.0936	GMEVIDTGAPLSVPVGGATLGR	18	------				
	1080.2350	3237.6809	GFKLILSGELDSLPEQAFYLVGNIDEATAK	59	------				
	1094.5599	4374.2119	ITSTKEGSITSIQAVYVPADDLTDPAPATTFAHLDATTVLSR	31	Deamidated (NQ)				
	1140.2444	3417.7279	VGLTALTMAEYFRDVNEQDVLLFIDNIFR	71	Oxidation (M)				
	1149.5512	3445.6348	GIYPAVDPLDSTSTMLQPQIVGEEHYETAQR	30	Deamidated (NQ)				
**165**	511.2485	1020.4910	DTDILAAMR	25	Oxidation (M)	Ribulose-1,5-bisphosphate carboxylase/oxygenase large subunit	49551/	gi|46326306/	----------
	511.2612	1020.5240	DTDILAAFR	54	-----		6.60	*Salvia chamaedryoides*	
	631.3499	1260.7078	DITLGFVDLLR	26	-----				
	756.0575	2265.1641	DITLGFVDLLRDDFVEKDR	72	-----				
**230**	515.2921	1028.5614	VIDEIRER	41	-----	Putative clathrin binding protein (epsin)	30781/	gi|3763925/	---------

							9.30	*Arabidopsis thaliana*	
**247**	668.3490	1334.6718	ATPDQVAEYTLK	49	------	Unknown	42861/	gi|118489355	fructose-bisphosphate
	727.4184	1452.8188	TVVSIPNGPSALAVK	25	Deamidated (NQ)		8.17	*Populus trichocarpa x*	aldolase
	1016.8556	3047.5451	YAAISQDNGLVPIVEPEILLDGEHGIER	6	Deamidated (NQ)			*Populus deltoides*	
	1096.5909	3286.6861	TLLVTVPGLGNYVSGAILFEETLYQSTTDGK	50	Deamidated (NQ)				
	1138.9478	3413.7970	TLLVTVPGLGNYVSGAILFEETLYQSTTDGKK	26	------				
**283**	701.9338	1401.7867	LFNAVAAEDLIVK	21	------	Unknown	29308/	gi|118488026	thiamine biosynthetic
	739.3988	2215.0865	EIVPGMIVTGMEVAEIDGAPR	34	2 Oxidation (M)		5.26	*Populus trichocarpa*	enzyme
	920.5126	2758.4476	VAIIEQSVSPGGGAWLGGQLFSAMIVR	19	Oxidation (M)				
**304**	582.9684	1745.9046	SEVSSLIAELASAAAAEK	82	------	Predicted protein	29052/	gi|224072767/	---------
	609.2783	1216.5400	NGWFYSLSDK	17	Deamidated (NQ)		5.69	*Populus trichocarpa*	
	646.3286	1290.6456	GIVFEEAIEER	36	------				
	714.8652	1427.7144	NDDLDAVLEVTPK	23	------				
	746.9221	1491.8272	LIWISAFMLVGAR	46	Oxidation (M)				
	752.8750	1503.7603	ALQEMGSGQDLLVK	38	Oxidation (M)				
	827.4336	1652.8410	GLGDADQVLAYFAVSK	57	------				
	1189.0599	2376.0773	WSDLVFFQNGMLEPWFQSK	6	2 Deamidated (NQ); Oxidation (M)				
**314**	620.3210	1857.9684	ELLVGKDDELLQTETR	28	-----	Predicted protein	27040/	gi|224090705/	NAD-dependent
	674.3518	1346.6830	IGGAEDVFVGDIR	56	-----		5.68	*Populus trichocarpa*	epimerase/dehydratase
**366**						ND			
**397**	472.5989	1414.7820	LEDLRIPPAYTK	24	------	Ribulose bisphosphate carboxylase large chain;	52923/	gi|1346967/	-----------
	489.2560	1464.7474	TFQGPPHGIQVER	23	------		5.88	*Brassica oleracea*	
	511.2739	1020.5240	DTDILAAFR	54	------				
	549.7805	2195.0786	AGVKEYKLNYYTPEYETK	17	------				
	710.8481	1419.6558	LNYYTPEYETK	19	------				
	808.4127	2422.1692	LNYYTPEYETKDTDILAAFR	15	------				
**445**						ND			
**470**	533.9519	1598.8093	IDWKETPEAHVFK	24	------	Heat shock protein 17.0	16954/	gi|1122315	-----------
	699.4177	1396.7926	AGLENGVLTVTVPK	21	------		5.78	*Pennisetum glaucum*	
**471**	538.2733	1611.7967	VIKDFMIQGGDFDK	34	------	Isomerase peptidyl-prolyl cis-trans isomerase	28146/	gi|224057792	-----------
	551.0230	2200.0471	VIKDFMIQGGDFDKGNGTGGK	10	Deamidated (NQ); Oxidation (M)		9.40	Populus trichocarpa	
	563.2989	1686.8651	HVVFGQVLEGMDIVK	51	Deamidated (NQ); Oxidation (M)				
	620.9422	1859.7996	DFMIQGGDFDK	28	-------				
	644.7828	1287.5442	DFMIQGGDFDK	41	Oxidation (M)				
	689.8859	1377.7293	VYFDIGIGNPVGK	74	-------				
	830.4510	1658.8549	RVVILESGELPMTEA.-	14	Oxidation (M)				
**484**	596.6628	1786.9828	IINEPTAAAIAYGLDKK	18	-------	BiP isoform B	73446/	gi|475600/	---------
	754.8726	1507.7307	ITPSWVAFTDSER	29	-------		5.11		
**485**	542.6755	1624.9260	QATKDAGVIAGLNVLR	27	-------	Unknown	71118/	gi|219885633	heat shock protein 70
	599.3807	1196.6877	DAGVIAGLNVLR	41	-------		5.10	*Zea mays*	
	737.3737	1472.6783	TTPSYVAFTDSER	29	-------				
	1009.5456	3025.4655	TLSSTAQTTIEIDSLYEGIDFYSTITR	24	Deamidated (NQ)				
**491**	516.3140	515.3179	ARAAK	34	-------	Hypothetical protein SORBIDRAFT_03g039980	60207/	gi|242054991	putative laccase
	663.3720	662.3533	-.MARAAK	27	Oxidation (M)		6.49	*Sorghum bicolor*	LAC6-8
**494**	541.3290	1080.6219	FEAIVYVLK	29	-------	Predicted protein	52710/	gi|224053971	Elongation factor Tu,
	573.3192	1144.6088	TGDTVDIVGLR	55	-------		6.00	*Populus trichocarpa*	chloroplastic
	595.3072	1782.8636	GITINTATVEYETESR	32	-------				
	1085.5626	2169.0801	QDQVDDEELLQLVELEVR	26	-------				
