# Supplementary material for: Effects of Heavy Metals and Arbuscular Mycorrhiza on the Leaf Proteome of a Selected Poplar Clone: A Time Course Analysis
Source: PLoS One. 2012 Jun 26;7(6):e38662. doi: 10.1371/journal.pone.0038662 (PMC3383689; doi:10.1371/journal.pone.0038662)
Supplement: Table S8 — Identification of poplar leaf proteins – second sampling (S2). Precursor ion m/z, calculated peptide mass, ion score, modification, protein name, theoretical molecular weight and pI, accession number and reference organism, and blast results for each identified spot. (PDF) [file pone.0038662.s009.pdf]

Table S8. Identification of poplar leaf proteins – second sampling (S2). Precursor ion m/z, calculated peptide mass, ion score, modification, protein name, theoretical molecular weight and pI, accession number and reference organism, and blast results for each identified spot.

Spot	Precursor ion *m/z*	Peptide mass calculated	Sequence	Ion Score	Modification	Protein	M_r (kDa) / pI Theor	AC number (gi NCBI) and reference organism	Blast results
**57**						ND			
**118**	503.7875	1005.5681	IGVCIGIFK	25	Carbamidomethyl (C)	Unknown	52057/	gi|118487547	Ribulose bisphosphate
	540.2826	1078.5481	NFMSLPNIK	36	Oxidation (M)		6.28	*Populus trichocarpa*	carboxylase/oxygenase
	586.2935	1170.5743	SFQCELVFAK	10	------				activase 1
	688.3626	2062.0517	LLEYGNMLVQEQENVKR	33	------				
	841.4304	2521.2296	VQLADKYLSEASLGEANQDSIDR	29	------				
	905.4978	2713.4439	EENPRVPIIVTGNDFSTLYAPLIR	81	------				
	934.9285	1867.8435	YLSEASLGEANQDSIDR	21	Deamidated (NQ)				
	941.9957	1881.9625	LVDTFPGQSIDFFGALR	81	------				
	961.9901	1921.9455	LLEYGNMLVQEQENVK	40	Oxidation (M)				
	970.4286	1938.8266	WGGLVTDMSDDQQDISR	37	Deamidated (NQ); Oxidation (M)				
	1045.0913	2088.1619	VPIIVTGNDFSTLYAPLIR	97	------				
	1176.5414	2351.0443	MGINPIMMSAGELESGNAGEPAK	23	3 Oxidation (M)				
	1179.0185	2356.0028	TYNLDNNMDGFYIAPAFMDK	16	Deamidated (NQ); Oxidation (M)				
	1320.6190	3958.8387	LGGTTQYTVNNQMVNATLMNIADNPTNVQLPGMYNK	10	2 Deamidated (NQ); 2 Oxidation (M)				
**119**	503.8128	1005.5681	IGVCIGIFK	11	Carbamidomethyl (C)	Unknown	52057/	gi|118487547	Ribulose bisphosphate
	540.3000	1078.5481	NFMSLPNIK	19	Oxidation (M)		6.28	*Populus trichocarpa*	carboxylase/oxygenase
	614.8326	1227.5958	SFQCELVFAK	47	Carbamidomethyl (C)				activase 1
	693.7224	2078.0466	LLEYGNMLVQEQENVKR	18	Oxidation (M)				
	701.3552	2100.9535	VPVAEGCTDPNAANFDPTAR	20	Carbamidomethyl (C)				
	806.4735	2416.3114	VPIIVTGNDFSTLYAPLIRDGR	13	------				
	807.8877	1613.7000	MCCLFINDLDAGAGR	42	Oxidation (M)				
	905.8504	2714.4279	EENPRVPIIVTGNDFSTLYAPLIR	60	Deamidated (NQ)				
	951.0197	3799.8653	VQLADKYLSEASLGEANQDSIDRGTFYGQAAQQVK	29	------				
	962.0250	1921.9455	LLEYGNMLVQEQENVK	26	Oxidation (M)				
	969.9731	1937.8425	WGGLVTDMSDDQQDISR	50	Oxidation (M)				
	1177.0720	2352.0283	MGINPIMMSAGELESGNAGEPAK	19	Deamidated (NQ); 3 Oxidation (M)				
**122**	599.8605	1197.7333	LSELLGIQVVK	26	------	Predicted protein	50181/	gi|224109060	phosphoglycerate
	720.3772	1438.7667	ELPGVLALDEVER	38	------		8.25	*Populus trichocarpa*	kinase, putative
	852.1398	2553.3901	AQGLPVGSSLVEEDKLGLATSLLEK	18	------				
	883.0134	1764.0145	LVASLPDGGVLLLENVR	39	------				
**132**	1061.0679	2120.1154	QTDLPFLLAVEDVFSITGR	57	------	Elongation factor Tu	52063/	gi|2494261	--------
							6.21		
**134**	503.7917	1005.5681	IGVCIGIFK	33	Carbamidomethyl (C)	Unknown	50638/	gi|118489408	Ribulose bisphosphate
	532.2766	1062.5532	NFMSLPNIK	18	------		8.36	*Populus trichocarpa x*	carboxylase/oxygenase
	576.8576	1151.7067	VPLILGIWGGK	60	------			*Populus deltoides*	activase 1
	586.2912	1170.5743	SFQCELVFAK	33	------				
	609.3292	1216.6452	WVSGVGVESIGK	30	------				
	838.8953	1675.7763	EGPPTFEQPAMTVEK	10	Oxidation (M)				
	941.9986	1881.9625	LVDTFPGQSIDFFGALR	32	------				
	1045.0913	2088.1619	VPIIVTGNDFSTLYAPLIR	87	------				
	1163.0307	2324.0334	MGISPIMMSAGELESGNAGEPAK	22	3 Oxidation (M)				
	1211.0051	2420.0011	TYNLDNMMDGYYIAPAFMDK	44	3 Oxidation (M)				
**135**	449.2632	1344.7402	KWVSGVGVESIGK	28	------	Unknown	51919/	gi|118486739	Ribulose bisphosphate
	449.2751	1344.7402	WVSGVGVESIGKK	12	------		5.26	*Populus trichocarpa*	carboxylase/oxygenase
	503.8064	1005.5681	IGVCIGIFK	44	Carbamidomethyl (C)				activase 1
	576.8779	1151.7067	VPLILGIWGGK	17	------				
	586.3162	1170.5743	SFQCELVFAK	20	------				
	653.0091	1955.9662	LFEYGNMLVKEQENVK	51	Oxidation (M)				
	744.7460	2231.1508	IGVCIGIFKTDNVPEDDIVK	33	Carbamidomethyl (C)				
	775.7164	2324.0334	MGISPIMMSAGELESGNAGEPAK	28	3 Oxidation (M)				
	1045.1338	2088.1619	VPIIVTGNDFSTLYAPLIR	23	------				
**137**	540.3044	1078.5481	NFMSLPNIK	23	Oxidation (M)	Unknown	52057/	gi|118487547	Ribulose bisphosphate
	586.3184	1170.5743	SFQCELVFAK	55	------		6.28	*Populus trichocarpa*	carboxylase/oxygenase
	806.4842	2416.3114	VPIIVTGNDFSTLYAPLIRDGR	23	------				activase 1
	807.8851	1613.7000	MCCLFINDLDAGAGR	24	Oxidation (M)				
	906.1000	2715.4119	EENPRVPIIVTGNDFSTLYAPLIR	28	2 Deamidated (NQ)				
	912.1473	2733.3135	MGINPIMMSAGELESGNAGEPAKLIR	36	3 Oxidation (M)				
	962.0250	1921.9455	LLEYGNMLVQEQENVK	23	Oxidation (M)				
	969.9526	1937.8425	WGGLVTDMSDDQQDISR	26	Oxidation (M)				
	1045.1338	2088.1619	VPIIVTGNDFSTLYAPLIR	21	------				

**142**	576.8531	1151.7067	VPLILGIWGGK	62	------	Unnamed protein product	51848/	gi|157345989	Ribulose bisphosphate
	693.6927	2078.0466	LLEYGNMLVQEQENVKR	24	Oxidation (M)		5.15	*Vitis vinifera*	carboxylase/oxygenase
	738.4152	2212.2330	NFMTLPNIKVPLILGIWGGK	17	2 Deamidated (NQ)				activase 1
	806.4256	2416.3114	VPIIVTGNDFSTLYAPLIRDGR	22	------				
	905.4752	2713.4439	EENPRVPIIVTGNDFSTLYAPLIR	60	------				
	912.1218	2733.3135	MGINPIMMSAGELESGNAGEPAKLIR	23	3 Oxidation (M)				
	941.9900	1881.9625	LVDTFPGQSIDFFGALR	69	------				
	961.9930	1921.9455	LLEYGNMLVQEQENVK	28	Oxidation (M)				
	1045.0913	2088.1619	VPIIVTGNDFSTLYAPLIR	110	------				
	1195.0118	2387.9814	QYNLDNTMDGFYIAPAFMDK	9	3 Deamidated (NQ); 2 Oxidation (M)				
	1325.3174	3972.8656	LGGTTQYTVNNQMVNATLMNIADNPTNVQLPGMYNK	11	3 Oxidation (M)				
**146**	500.2988	998.5913	FSLAPLVPR	32	------	Predicted protein	50181/	gi|224109060	chloroplast
	522.8161	1043.6226	LGLATSLLEK	48	------		8.25	Populus trichocarpa	phosphoglycerate
	550.8272	1099.6488	IGVIESLLEK	29	------				kinase
	645.3399	1932.9694	LASLADLYVNDAFGTAHR	36	------				
	688.0257	2061.0643	KLASLADLYVNDAFGTAHR	17	------				
	720.3998	1438.7667	ELPGVLALDEVER	69	------				
	781.7382	2342.1788	VGVADVMSHISTGGGASLELLEGK	30	Oxidation (M)				
	787.4150	1572.8359	GVTTIIGGGDSVAAVEK	5	------				
	790.7645	2369.2842	GVSLLLPSDVIIADKFAPDANSK	12	------				
	852.1344	2553.3901	AQGLPVGSSLVEEDKLGLATSLLEK	8	------				
	882.9967	1764.0145	LVASLPDGGVLLLENVR	26	------				
	1014.9737	2027.9396	ADLNVPLDDNQNITDDTR	44	------				
**148**	736.3966	735.3803	EEFLAK	45	------	Putative plastid isopentenyl diphosphate/dimethylallyl	49894/	gi|209402463	
						diphosphate synthase precursor	5.38	*Mantoniella squamata*	
**149**	748.4036	2989.5225	IFSHPDVVAEKPWYGLEQEYTLLQK	6	------	predicted protein	39200/	gi|224079530	cytosolic glutamine
	888.4955	1774.9617	IIAEYLWIGGSGLDIR	58	------		5.52	*Populus trichocarpa*	synthetase
	907.4762	2719.3713	RPASNMDPYVVTSMIAETTILWKP.-	15	------				
	918.9490	3671.7281	WNYDGSSTGQAPGQDSEVILYPQAIFRDPFRR	5	+ 2 Deamidated (NQ)				
	1172.5705	3514.6430	WNYDGSSTGQAPGQDSEVILYPQAIFRDPFR	20	Deamidated (NQ)				
**150**	506.8240	1011.5865	GPVIFNPLR	43	------	Predicted protein	44493/	gi|224145917	Uroporphyrinogen
	720.4426	1438.8143	ASSVAEPLLLNAVR	23	------		7.14	*Populus trichocarpa*	decarboxylase,
	1125.1040	2248.1046	LALTGVDVVSLDWSVDMAEGR	7	Oxidation (M)				putative
**152**	483.2923	1446.7831	IRDLFEQIVASR	44	Deamidated (NQ)	Predicted protein	44950/	gi|224071429	phosphoribulose
	499.9612	1496.7947	GHSLESIKASIEAR	36	------		5.90	*Populus trichocarpa*	kinase, putative
	567.3241	1698.8763	ILVIEGLHPMYDQR	18	Oxidation (M)				
	589.3276	1176.6139	DLFEQIVASR	58	-----				
	713.7428	2138.1259	VRDLLDFSIYLDISNEVK	38	-----				
	725.4028	2173.0804	HADFPGSNNGTGLFQTIVGLK	33	Deamidated (NQ)				
	744.3700	1486.6762	ANNFDLMYEQVK	12	Oxidation (M)				
	962.2809	3844.9047	KPDFDAYIDPQKQYADAVIEVLPTQLIPDDNEGK	9	------				
**155**	567.2906	1698.8763	ILVIEGLHPMYDQR	36	Oxidation (M)	Predicted protein	44950/	gi|224071429	phosphoribulose
	589.7942	1177.5979	DLFEQIVASR	64	Deamidated (NQ)		5.90	*Populus trichocarpa*	kinase, putative
	651.3429	1300.6776	LTSVFGGAAEPPR	30	------				
	654.3393	1960.0153	LDELIYVESHLSNISTK	28	------				
	672.3280	1342.6591	FYGEVTQQMLK	25	------				
	723.9039	1445.7990	IRDLFEQIVASR	69	------				
	725.3599	2173.0804	HADFPGSNNGTGLFQTIVGLK	94	Deamidated (NQ)				
	736.3482	1470.6813	ANNFDLMYEQVK	31	------				
	933.4186	2797.1966	FSYGPDAYYGHEVSVLEMDGQFDR	1	------				
	942.5022	1882.9564	DLLDFSIYLDISNEVK	10	Oxidation (M)				
	1206.1006	2410.1904	QYADAVIEVLPTQLIPDDNEGK	55	Gln->pyro-Glu (N-term Q)				
**161**	534.8035	1067.5652	YGVSGFPTLK	20	------	Unknown	34849/	gi|118482960	protein disulfide
	821.9500	1641.8191	YGVSGYPTLQWFPK	5	------		5.31	*Populus trichocarpa*	isomerase, putative
	925.4880	1848.8853	TAEALAEFVNNEGGSNVK	53	------				
**162**	497.6081	1489.7511	KLDLTADELSEEK	23	------	Predicted protein	35693/	gi|224102193	malate dehydrogenase,
	617.3362	2465.2088	NVIIWGNHSSSQYPDVNHATVK	9	------		6.11	*Populus trichocarpa*	putative
	672.7486	2015.1238	VLVTGAAGQIGYALVPMIAR	39	Oxidation (M)				
	681.8581	1361.6562	LDLTADELSEEK	27	------				
	681.8997	1361.7265	MELVDAAFPLLK	62	Oxidation (M)				
	682.3893	1362.7143	IVQGLSIDEFSR	71	------				
	812.1164	2433.2176	ELVKDDEWLNAEFITTVQQR	79	------				
	825.5364	1648.9876	VLVVANPANTNALILK	51	------				
	833.1736	2496.3886	EPVRVLVTGAAGQIGYALVPMIAR	31	Oxidation (M)				
	880.2050	2637.4741	VLVVANPANTNALILKEFAPSISEK	36	------				
	1070.1256	4276.2657	GVMLGPDQPVILHMLDIPPAAEALNGVKMELVDAAFPLLK	5	3 Oxidation (M)				
**163**	672.7437	2015.1238	VLVTGAAGQIGYALVPMIAR	29	Oxidation (M)	Cytosolic malate dehydrogenase	35475	gi|10334493	
	682.3942	1362.7143	IVQGLSIDEFSR	37	------		5.92	*Cicer arietinum*	
	825.5364	1648.9876	VLVVANPANTNALILK	1	------				

**164**	508.6300	1522.8103	YIGLSEASASTIRR	21	------	Predicted protein	37417/	gi|224069096	aldo/keto reductase
	536.9931	1607.8882	IKYIGLSEASASTIR	54	------		5.97	*Populus trichocarpa*	
	559.9821	1676.8522	LQLESIDLYYQHR	66	------				
	684.3841	1366.7092	YIGLSEASASTIR	18	------				
	688.6972	2062.9728	LTPEEMAELESIASSDAVR	48	Oxidation (M)				
	722.9382	1443.8085	ELGIGIVAYSPLGR	37	------				
	766.9298	1531.7994	IENFNQNVGALSVK	19	------				
**165**	681.8679	1361.7265	MELVDAAFPLLK	32	Oxidation (M)	Cytosolic malate dehydrogenase	35475/	gi|10334493	
	682.3698	1362.7143	IVQGLSIDEFSR	45	------		5.92	*Cicer arietinum*	
**166**	681.8874	1361.7265	MELVDAAFPLLK	80	Oxidation (M)	Cytosolic malate dehydrogenase	35475/	gi|10334493	---------
	682.3869	1362.7143	IVQGLSIDEFSR	45	-----		5.92	*Cicer arietinum*	
	825.5310	1648.9876	VLVVANPANTNALILK	14	-----				
**171**	941.9928	1881.9625	IVDTFPGQSIDFFGALR	39	------	Ribulose-bisphosphate carboxylase activase	25913/	gi|100380	---------
	1045.1065	2088.1619	VPIIVTGNDFSTLYAPLIR	24	------		5.01	*Nicotiana tabacum*	
**172**	682.3942	1362.7758	VLKGISVEEVYK	38	------	Hypothetical protein	20118/	gi|147835353	---------
	689.4034	1376.7915	VLKGISIEEVYK	32	------		5.54	*Vitis vinifera*	
**174**	668.3283	1334.6718	ATPDQVAEYTLK	16	------	Unknown	42861/	gi|118489355	fructose-bisphosphate
	727.4089	1452.8188	TVVSIPNGPSALAVK	17	Deamidated (NQ)		8.17	*Populus trichocarpa x*	aldolase, putative
	1138.9468	3413.7970	TLLVTVPGLGNYVSGAILFEETLYQSTTDGKK	13	------			*Populus deltoides*	
**181**	699.0285	2094.0970	TWGGRPENVNAAQEALLIR	32	------	Unknown	42799/	gi|118487575	fructose-bisphosphate
	726.9286	1451.8348	TVVSIPNGPSALAVK	16	------		7.55	*Populus trichocarpa*	aldolase, putative
	1012.1862	3033.5295	YAAISQDNGLVPIVEPEILLDGEHGIDR	34	Deamidated (NQ)				
**193**	760.4302	1518.8558	AAPLIALANYIAYR	16	------	GGDP synthase	39238/	gi|9971808	
	938.0094	1874.0401	SIGLLFQVVDDILDVTK	13	------		5.38	*Tagetes erecta*	
**202**	590.2951	1767.8753	MAEYAEELWELLKK	29	Oxidation (M)	Ferredoxin-NADP+ reductase	40139/	gi|5730139	
	820.8942	1639.7803	MAEYAEELWELLK	16	Oxidation (M)		8.66	*Arabidopsis thaliana*	
	1107.1062	2212.1932	FNGLAWLFLGVPTSSSLLYK	8	------				
**214**						ND			
**241**						ND			
**245**	597.6332	1789.9097	REDLPKYEEELELK	20	------	Predicted protein	28019/	gi|224081262	
	609.3315	1216.6411	EKAGLLSESQR	32	------		7.71	*Populus trichocarpa*	
	640.9894	1919.9476	IQYTIEEETKDIPDAR	46	------				
	655.8604	1309.7030	TYFLQLQEIR	52	------				
**246**	444.5784	1330.6670	LAWHSAGTFDVK	33	-------	Predicted protein	27320/	gi|224104631	ascorbate peroxidase
	545.3145	1632.8875	ALLSDPIFRPYVDK	20	-------		5.53	*Populus trichocarpa*	
	550.3257	1098.5921	EGLLQLPSDK	41	------				
	619.3618	1854.9938	ELLSGEKEGLLQLPSDK	26	------				
	624.3433	1869.9333	YSAELAHGANNGLDIAVR	55	------				
	625.3462	2497.2384	DVFGHMGLSDKDIVALSGGHTLGR	12	Oxidation (M)				
	688.3331	2061.8956	YAADEDAFFADYSEAHLK	14	------				
**253**	437.2737	872.5331	AKLDISVK	21	------	Predicted protein	27099/	gi|224141565	groes chaperonin,
	587.8311	1173.6605	TAGGLLLTEATK	54	------		7.77	*Populus trichocarpa*	putative
	876.1242	2625.3497	TESGILLPSSAQSKPQGGEVVAVGEGK	17	Deamidated (NQ)				
**254**	552.6504	1654.9294	TLLEDPVFRPLVEK	23	------	Putative ascorbate peroxidase	22449/	gi|46911557	
	633.9813	1898.9235	HPDELAHGANNGLDIAVR	35	Deamidated (NQ)		4.83	Populus x canadensis	
	688.9629	2063.8571	YAADEDAFFADYAEAHMK	33	------				
	1099.5398	1098.5233	LSELGFAEAY.-	30	------				
	1151.5536	2301.0742	SGFEGPWTPNPLVFDNSYFK	42	------				
**255**	729.4000	1456.8137	TGELKDIIGVPTSK	26	------	Predicted protein	30925/	gi|224130670	ribose-5-phosphate
	890.4665	2668.4146	EISGLEGVVEHGLFLDMATAVIIAGK	65	-----		5.36	*Populus trichocarpa*	isomerase, putative
	890.9701	3559.8774	SLNIPLSVLDDHPHIDLAIDGADEVDPLLNLVK	32	------				
	921.4173	1840.8401	MVEAASDEFVVVADDTK	36	Oxidation (M)				
	921.6819	3682.7467	AGEDGKPYVTDNFNYIVDLYFENPIKDGYAAGK	31	------				
	1007.8069	3020.4444	AGEDGKPYVTDNFNYIVDLYFENPIK	29	------				
**269**	473.5817	1417.7349	VYDFVLMLMKK	18	2 Oxidation (M)	Predicted protein	25422/	gi|224117556	tau class glutathione
	514.2393	1026.4811	FWADFVDK	58	------		5.31	*Populus trichocarpa*	transferase GSTU2
	578.2904	1154.5760	FWADFVDKK	45	------				
	645.8187	1289.6400	VYDFVLMLMK	34	2 Oxidation (M)				
	672.3693	1342.7278	SALLLQMNPVNK	58	Oxidation (M)				
	686.0183	2055.0421	ALEDPQKVYDFVLMLMK	54	Oxidation (M)				
	734.0505	2199.1319	ALEDPQKVYDFVLMLMKK	17	2 Oxidation (M)				
	837.4298	836.4280	DFIDSLK	27	------				
**272**	645.3328	1288.6816	QYYFLSVLTR	65	------	Hypothetical protein POPTRDRAFT_551203	28173/	gi|224062595	Oxygen-evolving
	795.3447	1588.7046	TNTDFLPYNGDGFK	37	Deamidated (NQ)		7.68	*Populus trichocarpa*	enhancer protein 2
	1153.5426	3457.6737	QSSLFATSSEGGFDTNTVATANILETSTPVIDGK	58	------				
**275**	497.2840	1488.7838	FQSVISQLFQHR	36	------	Predicted protein	32704/	gi|224068558	ATP-dependent Clp
	620.0432	1857.0247	EAKDYGLIDGVILNPLK	33	------		6.79	*Populus trichocarpa*	protease proteolytic
	765.4663	1528.8501	DYGLIDGVILNPLK	39	------				subunit, putative

**291**	536.2765	2141.0600	TADGDEGGKHQLITATVKDGK	4	Deamidated (NQ)	Hypothetical protein POPTRDRAFT_818640	28122/	gi|224085421	Oxygen-evolving
	628.8345	1255.6601	QYFFLSVLTR	75	Gln->pyro-Glu (N-term Q)		8.65	*Populus trichocarpa*	enhancer protein 2,
	782.3988	2344.1838	SITDYGSPEEFLSKVDFLLGK	83	------				chloroplast precursor
	786.8706	1571.7355	SITDYGSPEEFLSK	33	------				
	791.4606	790.4589	VDFLLGK	34	------				
	795.8523	1589.6886	TDTDFLPYNGDGFK	39	Deamidated (NQ)				
	797.0534	2388.1155	YEDNFDATSNVSVMVIPTDKK	33	Oxidation (M)				
**294**	576.8531	1151.7067	VPLILGIWGGK	27	------	Rubisco activase precursor	40800/	gi|3687652	
	941.9928	1881.9625	LVDTFPGQSIDFFGALR	11	------		7.59	*Datisca glomerata*	
	1045.0974	2088.1619	VPIIVTGNDFSTLYAPLIR	15	------				
**303**						ND			
**312**						ND			
**402**	753.4307	1504.7773	LLSENFPQLETSK	49	------	Esterase d, s-formylglutathione hydrolase	31900/	gi|224086942	
	863.5146	1724.9309	VASTEGIVLIAPDTSPR	37	------		6.17	*Populus trichocarpa*	
**403**	503.2490	1506.7579	APDNFRLDFAVSR	16	------	Predicted protein	40444/	gi|224074257	ferredoxin--NADP
	616.8308	1231.6482	GIDDIMVSLAAK	68	------		8.71	*Populus trichocarpa*	reductase, putative
	714.3665	1426.7304	EGQSIGVIPDGIDK	13	------				
	815.8928	1629.7886	LYSIASSAIGDFGDSK	43	------				
	820.8942	1639.7803	MAEYAEELWELLK	24	Oxidation (M)				
	884.9560	1767.8753	MAEYAEELWELLKK	22	Oxidation (M)				
	898.7292	2693.2068	ITGDDAPGETWHMVFSTEGEVPYR	8	------				
	1065.4848	1064.4927	AGQWNVEVY.-	21	------				
**409**	762.0389	2283.1059	QLTASGKPESFSGEFLVPSYR	21	Deamidated (NQ); Gln->pyro-Glu (N-term Q)	Putative protein	18483/	gi|190898996	Oxygen-evolving
	953.1254	2856.3957	LTYTLDEIEGPFEVSPDGTIKFEEK	42	------		5.17	*Populus tremula*	enhancer protein 1
**410**	482.7917	963.5793	VPFLFTIK	62	------	Photosystem II protein 33kD	26645/	gi|224916	--------
	762.0493	2283.1535	QLVASGKPESFSGDFLVPSYR	19	------		5.01		
	880.9393	1759.8741	DGIDYAAVTVQLPGGER	22	------				
	902.8256	2705.4429	DGIDYAAVTVQLPGGERVPFLFTIK	7	------				
**411**	482.7876	963.5793	VPFLFTIK	48	------	Unknown	35133/	gi|118489901	Oxygen-evolving
	636.3201	1270.6268	FCLEPTSFTVK	14	------		5.62	*Populus trichocarpa x*	enhancer protein 1,
	741.1255	2960.4363	GGSTGYDNAVALPAGGRGDEEELLKENIK	14	Deamidated (NQ)			*Populus deltoides*	chloroplast precursor
	781.8797	1561.7485	GGSTGYDNAVALPAGGR	38	------				
	826.0725	2475.1877	SKPETGEIIGVFESLQPSDTDLGAK	9	------				
	873.4570	2617.3123	SKPETGEIIGVFESLQPSDTDLGAK	67	------				
	881.4514	1760.8581	DGIDYAAVTVQLPGGER	35	Deamidated (NQ)				
	953.1514	2856.3957	LTYTLDEIEGPFEVSPDGTIKFEEK	65	------				
	1150.5995	4598.2592	LTYTLDEIEGPFEVSPDGTIKFEEKDGIDYAAVTVQLPGGER	29	------				
	1162.5864	2323.1471	LTYTLDEIEGPFEVSPDGTIK	12	------				
	1220.6312	1219.6237	IQGIWYAQLE.-	22	------				
**414**	922.4391	921.4444	LSELGFADA.-	8	------	Ascorbate peroxidase	27481/	gi|42558486	
	1016.4884	3046.4600	SGFEGPWTANPLIFDNSYFKELLSGEK	12	Deamidated (NQ)		5.52	*Rehmannia glutinosa*	
	1145.5461	2289.0742	SGFEGPWTANPLIFDNSYFK	54	------				
**415**	478.7631	955.5280	FFAVGLFR	37	------	Predicted protein	33600/	gi|224146717	Psb29 like protein
	541.7871	1081.5808	EAIFQAYIK	19	------		7.59	*Populus trichocarpa*	(thylakoid formation 1)
	697.8418	1393.6838	AQTPSSLVDFSSR	61	------				
	780.4047	1558.7838	EGEIEGTLKDIAER	42	------				
**419**	820.7787	2459.2889	GPLVPLLGVDVWEHAYYLQYK	25	------	Predicted protein	25299/	gi|224124440	manganese superoxide
	843.4520	1684.8883	LVVETTENQDPLVTK	34	------		6.80	*Populus trichocarpa*	dismutase
**420**	503.7875	1005.5681	IGVCIGIFK	47	Carbamidomethyl (C)	Unknown	52038/	gi|118489105	ribulose-1,5-
	540.2913	1078.5481	NFMSLPNIK	20	Oxidation (M)		6.28	*Populus trichocarpa x*	bisphosphate
	576.8688	1151.7067	VPLILGIWGGK	53	------			*Populus deltoides*	carboxylase/oxygenase
	586.2935	1170.5743	SFQCELVFAK	13	------				activase 2
	738.4127	2212.2442	NFMSLPNIKVPLILGIWGGK	18	Oxidation (M)				
	941.9928	1881.9625	LVDTFPGQSIDFFGALR	51	------				
	1045.1004	2088.1619	VPIIVTGNDFSTLYAPLIR	69	------				
	1195.0312	2387.9926	TYNLDNNMDGYYIAPAFMDK	55	Deamidated (NQ); 2 Oxidation (M)				
**421**	788.6544	3150.6350	TTGIVLDSGDGVSHTVPIYEGYALPHAILR	34		Actin	17210/	gi|2887459	
	887.9591	1773.8897	NYELPDGQVITIGAER	19			4.73	*Cucumis sativus*	
	928.4619	1854.9251	LAYVALDYEQELETAK	15					
	1100.5565	3298.6076	MTQIMFETFNVPAMYVAIQAVLSLYASGR	77	3 Oxidation (M)				
**423**	475.7936	949.5637	VPFLFTVK	45		Putative protein	18483/	gi|190898996	Oxygen-evolving
	767.3894	2299.1485	QLTASGKPESFSGEFLVPSYR	11			5.17	*Populus tremula*	enhancer protein 1
	898.1420	2691.4272	DGIDYAAVTVQLPGGERVPFLFTVK	8					
