# Supplementary material for: Effects of Heavy Metals and Arbuscular Mycorrhiza on the Leaf Proteome of a Selected Poplar Clone: A Time Course Analysis
Source: PLoS One. 2012 Jun 26;7(6):e38662. doi: 10.1371/journal.pone.0038662 (PMC3383689; doi:10.1371/journal.pone.0038662)
Supplement: Table S9 — Identification of poplar leaf proteins – third sampling (S3). Precursor ion m/z, calculated peptide mass, ion score, modification, protein name, theoretical molecular weight and pI, accession number and reference organism, and blast results for each identified spot. (PDF) [file pone.0038662.s010.pdf]

Table S9. Identification of poplar leaf proteins – third sampling (S3). Precursor ion m/z, calculated peptide mass, ion score, modification, protein name, theoretical molecular weight and pI, accession number and reference organism, and blast results for each identified spot.

Spot	Precursor ion *m/z*	Peptide mass calculated	Sequence	Ion Score	Modification	Protein	M_r (kDa) / pI Theor	AC number (gi NCBI) and reference organism	Blast results
**85**	593.3487	1184.6513	DAGAISGLNVLR	22	------	Heat shock 70 kDa protein	70835/	gi|123601	---------
	744.3879	1486.6940	TTPSYVAFTDTER	40	------		5.37		
**105**	650.8741	1299.7584	VMQAIGVLLNVK	34	Oxidation (M)	Predicted protein	65463/	gi|224071575	heat shock protein 70 (HSP70)-
	877.9120	1753.8006	ATELDDEDISYLTNR	6	------		6.17	*Populus trichocarpa*	interacting protein
**118**	457.7834	913.5596	GILNVSAIK	46	------	Predicted protein	61953/	gi|224104681	Putative rubisco subunit
	467.5889	1399.7671	TVQGLVEELEKR	21	------		5.24	*Populus trichocarpa*	binding-protein alpha subunit=
	473.2492	1416.7361	GRNVVLDEFGSPK	31	------				chaperonin 60 alpha subunit
	484.9713	1451.8963	ISAIKDIIPLLEK	38	------				
	522.2954	1042.5771	VVNDGVTIAR	34	------				
	557.6287	1669.8999	IKDADERLGADIVQK	19	------				
	562.0003	1682.9931	LGLLSVTSGANPVSIKK	73	------				
	591.8335	1181.6768	LADAVGLTLGPR	57	------				
	602.8144	1203.6136	NVVLDEFGSPK	61	------				
	605.3418	1812.9945	GIDKTVQGLVEELEKR	35	------				
	611.3347	2441.3027	GRNVVLDEFGSPKVVNDGVTIAR	10	------				
	635.6535	1903.9487	DSTTIIADAASKDELQAR	44	------				
	657.6381	1969.9116	ELSETDSVYDSEKLAER	18	------				
	680.4048	2038.2038	EIIKLGLLSVTSGANPVSIK	52	------				
	689.3911	2065.1531	AALQSGIDKLADAVGLTLGPR	83	------				
	705.7114	2114.1219	LSGGVAVIKVGAATETELEDR	63	------				
	723.1122	2166.2987	EIIKLGLLSVTSGANPVSIKK	36	------				
	735.9057	2939.5869	NATFAAIEEGIVPGGGAALVHLSTHVPAIK	23	------				
	740.3728	1478.7405	GYISPQFVTNPEK	11	------				
	743.7359	2228.1801	NVVLDEFGSPKVVNDGVTIAR	41	------				
	746.1332	2235.3453	VLVTDQKISAIKDIIPLLEK	23	------				
	760.0994	2277.2580	ALVAPASLIAQNAGIEGEVVVEK	27	------				
	771.0760	2310.1889	AIELPDPMENAGAALIREVASK	9	Oxidation (M)				
	778.4497	1554.8981	LGLLSVTSGANPVSIK	15	------				
	818.8891	1635.7700	TNDSAGDGTTTASVLAR	34	------				
	898.9737	1795.9138	AIELPDPMENAGAALIR	89	Oxidation (M)				
**132**	401.2373	1200.6979	VVDLLAPYRR	16	------	ATP synthase beta subunit	51910/	gi|62085107	------------
	443.5630	1327.6633	AHGGVSVFGGVGER	36	------		5.05	*Cespedesia bonplandii*	
	488.2737	974.5549	IGLFGGAGVGK	52	------				
	504.2926	1006.5699	LSIFETGIK	60	------				
	523.3070	1044.5968	VVDLLAPYR	66	------				
	595.8242	1189.6343	SAPAFIQLDTK	27	------				
	597.6309	1789.8855	MRVGLTALTMAEYFR	24	2 Oxidation (M)				
	639.3529	1276.6849	MPNIYNALVVK	29	Oxidation (M)				
	678.7309	2033.1561	LSIFETGIKVVDLLAPYR	25	------				
	717.3938	1432.7674	FVQAGSEVSALLGR	79	------				
	744.3751	1486.7490	VGLTALTMAEYFR	57	Oxidation (M)				
	744.9328	1487.8269	TVLIMELINNIAK	56	Deamidated (NQ); Oxidation (M)				
	751.3968	3001.5971	YKELQDIIAILGLDELSEEDRLTVAR	32	------				
	809.3965	1616.7981	VALVYGQMNEPPGAR	31	Oxidation (M)				
	969.5235	2905.4960	LILSGELDSLPEQAFYLVGNIDEATAK	39	------				
	975.5134	1948.9894	DVNEQDVLLFIDNIFR	58	------				
	996.8853	2987.5797	FLSQPFFVAEVFTGSPGKYVGLVETIR	57	------				
**171**	487.6090	1459.8147	IRDLFEQIIASR	52	------	Predicted protein	44997/	gi|224138316	Phosphoribulose kinase,
	567.2861	1698.8763	ILVIEGLHPMYDQR	31	Oxidation (M)		6.11	*Populus trichocarpa*	putative
	596.3277	1190.6295	DLFEQIIASR	52	------				
	713.7177	2138.1259	VRDLLDFSIYLDISNEVK	13	------				
	725.3650	2173.0804	HADFPGSNNGTGLFQTIVGLK	42	Deamidated (NQ)				
	737.8371	1473.6446	ANDFDLMYDQVK	24	Oxidation (M)				
	942.4906	1882.9564	DLLDFSIYLDISNEVK	35	------				

**176**	442.8762	1325.6153	FYWAPTRDDR	30	------	Unknown	52038/	gi|118489105	Ribulose bisphosphate
	448.8798	1343.6332	MEKFYWAPTR	21	Oxidation (M)		6.28	*Populus trichocarpa x*	carboxylase/oxygenase activase
	464.9150	1391.7231	DDRIGVCIGIFK	25	Carbamidomethyl (C)			*Populus deltoides*	1
	470.7361	939.4603	FYWAPTR	31	------				
	503.7833	1005.5681	IGVCIGIFK	41	Carbamidomethyl (C)				
	540.2782	1078.5481	NFMSLPNIK	41	Oxidation (M)				
	576.8621	1151.7067	VPLILGIWGGK	61	------				
	577.5986	1729.7882	MEKFYWAPTRDDR	25	Oxidation (M)				
	586.2912	1170.5743	SFQCELVFAK	19	------				
	694.0187	2079.0306	LLEYGNMLVQEQENVKR	67	Deamidated (NQ); Oxidation (M)				
	784.6869	2351.0443	MGINPIMMSAGELESGNAGEPAK	48	3 Oxidation (M)				
	792.3839	3165.4675	TYNLDNNMDGYYIAPAFMDKLVVHISK	23	2 Deamidated (NQ); 2 Oxidation (M)				
	797.0058	2387.9926	TYNLDNNMDGYYIAPAFMDK	23	Deamidated (NQ); 2 Oxidation (M)				
	806.4496	2416.3114	VPIIVTGNDFSTLYAPLIRDGR	35	------				
	828.3507	1654.7266	MCCLFINDLDAGAGR	16	Carbamidomethyl (C)				
	841.4141	2521.2296	VQLADKYLSEASLGEANQDSIDR	86	------				
	912.1190	2733.3135	MGINPIMMSAGELESGNAGEPAKLIR	59	3 Oxidation (M)				
	941.9957	1881.9625	LVDTFPGQSIDFFGALR	83	------				
	950.9908	3799.8653	VQLADKYLSEASLGEANQDSIDRGTFYGQAAQQVK	20	------				
	961.9814	1921.9455	LLEYGNMLVQEQENVK	45	Oxidation (M)				
	1045.1004	2088.1619	VPIIVTGNDFSTLYAPLIR	69	------				
**178**	453.9206	1358.7558	WITGVGVDSIGKK	18	------	Ribulose bisphosphate carboxylase/oxygenase	48046/	gi|3914605	-----------
	453.9206	1358.7558	KWITGVGVDSIGK	20	------	activase	8.20		
	470.7341	939.4603	FYWAPTR	31	------				
	496.6257	1486.8508	KWITGVGVDSIGKK	7	------				
	508.3002	1521.9031	VPLILGIWGGKGQGK	9	------				
	576.8553	1151.7067	VPLILGIWGGK	51	------				
	586.2912	1170.5743	SFQCELVFAK	18	------				
	616.3397	1230.6609	WITGVGVDSIGK	42	------				
	693.7001	2078.0466	LLEYGNMLVQEQENVKR	32	Oxidation (M)				
	806.4469	2416.3114	VPIIVTGNDFSTLYAPLIRDGR	15	------				
	905.8363	2714.4279	EENPRVPIIVTGNDFSTLYAPLIR	31	Deamidated (NQ)				
	941.9957	1881.9625	LVDTFPGQSIDFFGALR	54	------				
	1045.5945	2089.1459	VPIIVTGNDFSTLYAPLIR	58	Deamidated (NQ)				
**197**	593.8351	1185.6605	TVGAGVIQSIIE.-	14	------	Predicted protein	46575/	gi|224074859	Elongation factor Tu,
	642.3310	1923.9645	TTLTAALTMALASMGGSAPK	34	2 Oxidation (M)		5.60	*Populus trichocarpa*	chloroplastic
	779.4335	1556.8410	EGGKTVGAGVIQSIIE.-	9	------				
	841.4767	1680.9410	ILDEALAGDNVGLLLR	84	------				
	892.4481	1782.8636	GITINTATVEYETESR	75	------				
	1061.0618	2120.1154	QTDLPFLLAVEDVFSITGR	58	------				
**199**	449.2552	1344.7402	WVSGVGVESIGKK	11	------	Unknown	50658/	gi|118489408	Ribulose bisphosphate
	449.2572	1344.7402	KWVSGVGVESIGK	40	------		8.36	*Populus trichocarpa x*	carboxylase/oxygenase activase
	470.7361	939.4603	FYWAPTR	28	------			*Populus deltoides*	1
	491.9511	1472.8351	KWVSGVGVESIGKK	17	------				
	540.2761	1078.5481	NFMSLPNIK	40	Oxidation (M)				
	576.8621	1151.7067	VPLILGIWGGK	51	------				
	586.2844	1170.5743	SFQCELVFAK	13	------				
	609.3315	1216.6452	WVSGVGVESIGK	47	------				
	705.0217	2112.0673	LFEYGNMLVKEQENVKR	21	Oxidation (M)				
	806.4602	2416.3114	VPIIVTGNDFSTLYAPLIRDGR	28	------				
	807.6746	2420.0011	TYNLDNMMDGYYIAPAFMDK	6	3 Oxidation (M)				
	839.4004	1676.7603	EGPPTFEQPAMTVEK	49	Deamidated (NQ); Oxidation (M)				
	900.8297	2699.4283	EDNPRVPIIVTGNDFSTLYAPLIR	55	------				
	903.1298	2706.3026	MGISPIMMSAGELESGNAGEPAKLIR	18	3 Oxidation (M)				
	941.9957	1881.9625	LVDTFPGQSIDFFGALR	77	------				
	978.9772	1955.9662	LFEYGNMLVKEQENVK	15	Oxidation (M)				
	1045.0944	2088.1619	VPIIVTGNDFSTLYAPLIR	76	------				
	1163.0403	2324.0334	MGISPIMMSAGELESGNAGEPAK	24	3 Oxidation (M)				
**200**	540.2739	1078.5481	NFMSLPNIK	6	Oxidation (M)	Ribulose-1,5-bisphosphate carboxylase/oxygenase	48324/	gi|12620883	-----------
	576.8553	1151.7067	VPLILGIWGGK	41	------	activase 2	5.06	*Gossypium hirsutum*	
	806.7717	2417.2955	VPIIVTGNDFSTLYAPLIRDGR	9	Deamidated (NQ)				
	941.9871	1881.9625	LVDTFPGQSIDFFGALR	64	------				
	1045.5945	2089.1459	VPIIVTGNDFSTLYAPLIR	32	Deamidated (NQ)				
**204**						ND			

**209**	442.8683	1325.6153	FYWAPTRDDR	26	------	Unknown	52038/ 6.28	gi|118489105 *Populus trichocarpa x Populus deltoides*	Ribulose bisphosphate carboxylase/oxygenase activase
	470.7402	939.4603	FYWAPTR	31	------				
	503.7875	1005.5681	IGVCIGIFK	45	Carbamidomethyl (C)				
	508.3002	1521.9031	VPLILGIWGGKGQGK	17	------				
	540.2782	1078.5481	NFMSLPNIK	32	Oxidation (M)				
	576.8621	1151.7067	VPLILGIWGGK	60	------				
	586.2957	1170.5743	SFQCELVFAK	14	-------				
	670.3388	2677.3307	RVQLADKYLSEASLGEANQDSIDR	30	------				
	694.0137	2079.0306	LLEYGNMLVQEQENVKR	52	Deamidated (NQ); Oxidation (M)				
	704.0439	2109.1007	LVDTFPGQSIDFFGALRAR	22	------				
	738.4254	2212.2442	NFMSLPNIKVPLILGIWGGK	45	Oxidation (M)				
	784.6843	2351.0443	MGINPIMMSAGELESGNAGEPAK	37	3 Oxidation (M)				
	792.1385	3164.4834	TYNLDNNMDGYYIAPAFMDKLVVHISK	17	Deamidated (NQ); 2 Oxidation (M)				
	806.4496	2416.3114	VPIIVTGNDFSTLYAPLIRDGR	28	------				
	841.4196	2521.2296	VQLADKYLSEASLGEANQDSIDR	52	------				
	941.9986	1881.9625	LVDTFPGQSIDFFGALR	102	------				
	961.9581	1921.9455	LLEYGNMLVQEQENVK	16	Oxidation (M)				
	1045.0974	2088.1619	VPIIVTGNDFSTLYAPLIR	67	------				
	1195.0215	2387.9926	TYNLDNNMDGYYIAPAFMDK	27	Deamidated (NQ); 2 Oxidation (M)				
**212**	442.8743	1325.6153	FYWAPTRDDR	24	------	Unknown	52057/ 6.28	gi|118487547 *Populus trichocarpa*	Ribulose bisphosphate Carboxylase/oxygenase activase
	448.8778	1343.6332	MEKFYWAPTR	28	Oxidation (M)				
	464.9069	1391.7231	DDRIGVCIGIFK	29	Carbamidomethyl (C)				
	470.7341	939.4603	FYWAPTR	32	------				
	503.7896	1005.5681	IGVCIGIFK	46	Carbamidomethyl (C)				
	540.7708	1079.5321	NFMSLPNIK	41	Deamidated (NQ); Oxidation (M)				
	577.6121	1729.7882	MEKFYWAPTRDDR	27	Oxidation (M)				
	586.2980	1170.5743	SFQCELVFAK	36	------				
	671.3124	2681.1988	QTDKDRWGGLVTDMSDDQQDISR	45	Oxidation (M)				
	694.0187	2079.0306	LLEYGNMLVQEQENVKR	60	Deamidated (NQ); Oxidation (M)				
	697.0595	2088.1619	VPIIVTGNDFSTLYAPLIR	67	------				
	761.4077	3041.5934	EENPRVPIIVTGNDFSTLYAPLIRDGR	4	------				
	784.6922	2351.0443	MGINPIMMSAGELESGNAGEPAK	73	3 Oxidation (M)				
	806.4496	2416.3114	VPIIVTGNDFSTLYAPLIRDGR	45	------				
	841.4196	2521.2296	VQLADKYLSEASLGEANQDSIDR	31	------				
	905.4696	2713.4439	EENPRVPIIVTGNDFSTLYAPLIR	54	------				
	912.1218	2733.3135	MGINPIMMSAGELESGNAGEPAKLIR	35	3 Oxidation (M)				
	934.4413	1866.8595	YLSEASLGEANQDSIDR	29	------				
	941.9928	1881.9625	LVDTFPGQSIDFFGALR	68	------				
	961.9901	1921.9455	LLEYGNMLVQEQENVK	67	Oxidation (M)				
	969.9351	1937.8425	WGGLVTDMSDDQQDISR	90	Oxidation (M)				
	1325.6314	3973.8496	LGGTTQYTVNNQMVNATLMNIADNPTNVQLPGMYNK	13	Deamidated (NQ); 3 Oxidation (M)				
**215**	452.2465	902.4974	LFSPGNLR	62	------	Predicted protein	42412/ 5.77	gi|224112589 *Populus trichocarpa*	Chloroplast sedoheptulose-1,7-bisphosphatase
	454.7758	907.5379	TTYVLALK	42	------				
	456.7550	911.5116	LINYYVK	19	------				
	498.2670	1491.7933	EKGVFTNVISPSSK	27	------				
	537.9471	1610.8304	NEIIRFEETLYGK	27	------				
	618.3381	1234.6558	GVFTNVISPSSK	45	------				
	825.4341	1648.8495	YTGGMVPDVNQIIVK	35	Oxidation (M)				
**216**	488.6155	1462.8548	FLKPSVSGFLLQK	29	------	Predicted protein	50181/ 8.25	gi|224109060 *Populus trichocarpa*	Phosphoglycerate kinase, putative
	500.2946	998.5913	FSLAPLVPR	54	------				
	522.8096	1043.6226	LGLATSLLEK	28	------				
	599.8720	1197.7333	LSELLGIQVVK	26	------				
	645.6543	1933.9534	LASLADLYVNDAFGTAHR	44	Deamidated (NQ)				
	688.0282	2061.0643	KLASLADLYVNDAFGTAHR	14	------				
	696.3717	1390.7344	ELDYLVGAVSTPK	10	------				
	720.3847	1438.7667	ELPGVLALDEVER	48	------				
	766.7091	2297.1248	ADLNVPLDDNQNITDDTRIR	28	------				
	787.4203	1572.8359	GVTTIIGGGDSVAAVEK	5	------				
	790.7750	2369.2842	GVSLLLPSDVIIADKFAPDANSK	12	------				
	852.1344	2553.3901	AQGLPVGSSLVEEDKLGLATSLLEK	15	------				
	854.9799	1707.9407	ELPGVLALDEVERVAV.-	34	------				
	883.0134	1764.0145	LVASLPDGGVLLLENVR	46	------				
	1014.9677	2027.9396	ADLNVPLDDNQNITDDTR	69	------				
**223**	487.6049	1459.8147	IRDLFEQIIASR	50	------	Predicted protein	44997/ 6.11	gi|224138316 *Populus trichocarpa*	Phosphoribulose kinase, putative
	567.3062	1698.8763	ILVIEGLHPMYDQR	27	Oxidation (M)				
	680.3242	1358.6540	FYGEVTQQMLK	12	Oxidation (M)				
	725.3751	2173.0804	HADFPGSNNGTGLFQTIVGLK	40	Deamidated (NQ)				
	737.8244	1473.6446	ANDFDLMYDQVK	36	Oxidation (M)				

**227**	482.9380	1445.7990	IRDLFEQIVASR	40	------	Predicted protein	44950/	gi|224071429	Phosphoribulose kinase,
	499.9445	1496.7947	GHSLESIKASIEAR	44	------		5.90	*Populus trichocarpa*	putative
	529.7708	2115.0378	DMAERGHSLESIKASIEAR	11	Oxidation (M)				
	567.2973	1698.8763	ILVIEGLHPMYDQR	34	Oxidation (M)				
	589.3025	1176.6139	DLFEQIVASR	52	------				
	651.3453	1300.6776	LTSVFGGAAEPPR	29	------				
	680.8206	1359.6380	FYGEVTQQMLK	28	Deamidated (NQ); Oxidation (M)				
	713.7177	2138.1259	VRDLLDFSIYLDISNEVK	24	------				
	725.3776	2173.0804	HADFPGSNNGTGLFQTIVGLK	60	Deamidated (NQ)				
	744.3470	1486.6762	ANNFDLMYEQVK	14	Oxidation (M)				
	901.2265	3600.8689	HADFPGSNNGTGLFQTIVGLKIRDLFEQIVASR	12	Deamidated (NQ)				
	942.5079	1882.9564	DLLDFSIYLDISNEVK	18	------				
	1054.8143	4215.1263	KPDFDAYIDPQKQYADAVIEVLPTQLIPDDNEGKVLR	21	2 Deamidated (NQ				
**236**	477.9512	1430.8344	IAATSSTGKLELLK	34	------	Unknown	40586/	gi|118488941	Alcohol dehydrogenase,
	584.3363	1166.6659	VVAAALNPVDAK	37	------		8.49	*Populus trichocarpa x*	putative
	637.3752	1909.1248	SILVLNGAGGVGSLVIQLAK	40	Deamidated (NQ)			*Populus deltoids*	
	722.0186	2163.0273	GPFTFSQVAEAFSYIETNR	30	------				
	835.4480	2503.2958	NLDFIQAAGLPLAIETAYEGLER	61	------				
	857.9509	1713.9050	EGGSVVALTGAVTPPGFR	25	------				
**238**	488.9596	1463.7732	LYEIGAGTSEIRR	21	------	Isovaleryl-CoA Dehydrogenase; auxin binding	44517/	gi|5869965	
	496.3124	990.5750	GITAFIIEK	47	------	protein (ABP44)	6.27	*Pisum sativum*	
**241**	430.7355	859.4704	FAALPWR	12	------	Hypothetical protein	40450/	gi|225446767	Aldo/keto reductase, putative
	832.3999	1662.8114	NAEQAKEFSGALGWR	29	------		6.69	*Vitis vinifera*	
**244**	540.3284	1617.8726	VLAPYSSEDARGLLK	16	------	Predicted protein	38561/	gi|224053535	Pyruvate dehydrogenase,
	568.6489	1702.8348	IAGADVPMPYAANLER	25	Oxidation (M)		5.87	*Populus trichocarpa*	putative
	619.8845	1237.7030	LAVPQVEDIVR	21	------				
**247**	477.9532	1430.8344	IAATSSTGKLELLK	48	------	Unknown	40586/	gi|118488941	Alcohol dehydrogenase,
	584.3295	1166.6659	VVAAALNPVDAK	50	------		8.49	*Populus trichocarpa x*	putative
	590.8054	1179.6136	FVVTSNGNTLK	33	Deamidated (NQ)			*Populus deltoides*	
	633.8373	1265.6503	SLGADLAIDYTK	33	------				
	662.3607	1984.0517	ATDSPLPTVPGYDVAGVVVK	27	------				
	666.8944	2663.5123	SILVLNGAGGVGSLVIQLAKHVFGASR	28	Deamidated (NQ)				
	681.0481	2040.1368	VVKEGGSVVALTGAVTPPGFR	46	------				
	754.0818	2259.2151	FKATDSPLPTVPGYDVAGVVVK	54	------				
	761.7517	2282.1794	FVVTSNGNTLKTLNPYLESGK	11	Deamidated (NQ)				
	835.4480	2503.2958	NLDFIQAAGLPLAIETAYEGLER	38	------				
	857.9619	1713.9050	EGGSVVALTGAVTPPGFR	75	------				
	870.8271	2609.4065	ATDSPLPTVPGYDVAGVVVKVGNQVK	3	------				
	955.5811	1909.1248	SILVLNGAGGVGSLVIQLAK	67	Deamidated (NQ)				
	1082.5194	2163.0273	GPFTFSQVAEAFSYIETNR	73	------				
**261**	412.2286	1233.6717	DLREGLQLYK	12	------	Predicted protein	38389/	gi|224073126	----------
	425.7326	849.4596	EGLQLYK	21	------		5.87	*Populus trichocarpa*	
	432.2153	1293.6387	TAEGFEPLMKR	31	Oxidation (M)				
	433.9067	1298.6942	IRTDPDLSNLR	36	------				
	470.7666	939.5178	SLFGFNKK.-	29	------				
	485.2418	968.4716	AGFEDFKR	24	------				
	489.2643	1464.7507	VREIQMQNYLR	25	Oxidation (M)				
	515.7625	1029.5091	TDPDLSNLR	54	------				
	683.3373	2046.9568	YGNMDYTGELTEKEIIR	38	Oxidation (M)				
**270**	489.2643	1464.7474	TFQGPPHGIQVER	20	------	Ribulose-1,5-bisphosphate carboxylase/oxygenase	52000/	gi|1293020	----------
	493.2972	984.5716	ALRLEDLR	20	------	large subunit	6.10	*Polyscias guilfoylei*	
	511.2718	1020.5240	DTDILAAFR	67	------				
**277**	489.2540	1464.7474	TFQGPPHGIQVER	5	------	Ribulose-1,5-bisphosphate carboxylase/oxygenase	49551/	gi|46326306	----------
	511.2549	1020.4910	DTDILAAMR	35	Oxidation (M)	large subunit	6.60	*Salvia chamaedryoides*	
	511.2612	1020.5240	DTDILAAFR	41	------				
**279**	472.6315	1414.8184	LEDLRIPVAYVK	20	------	Ribulose-1,5-bisphosphate carboxylase/oxygenase	48604/	gi|14585745	
	489.2913	1464.7838	TFQGPPHGIKVER	33	------	large subunit	6.80	*Veronica arguta*	
**286**	541.6605	1621.9879	AISKPLQLVLANSLR	29	------	Predicted protein	30263/	gi|224110036	----------
	638.3435	1274.6904	LGALLDTMNALK	31	Oxidation (M)		5.36	*Populus trichocarpa*	
	699.3929	1396.7562	KVDDPELLEAIR	22	------				
**289**	702.9077	1403.7409	YAVIQGEQGAVIR	19	Deamidated (NQ)	Chain A, Profilin I	14100/	gi|157836856	
	710.8931	1419.7358	YSVIQGEQGAVIR	28	Deamidated (NQ)		4.70	*Arabidopsis thaliana*	
**290**	503.2679	1506.7579	APDNFRLDFAVSR	35	------	Predicted protein	40444/	gi|224074257	Ferredoxin--NADP reductase,
	815.9008	1629.7886	LYSIASSAIGDFGDSK	31	------		8.71	*Populus trichocarpa*	putative
**293**	423.2373	1266.7044	SGTVEPRPGVLR	32	------	Predicted protein	28920/	gi|224093744	2-deoxyglucose-6-phosphate
	566.3064	1130.5972	LIDTLQDWK	31	------		5.12	*Populus trichocarpa*	phosphatase
	723.8787	1445.7514	DAIAIYPDLSNVR	43	------				
**295**	494.7926	987.5753	AGGYGLIIPK	13	------	Predicted protein	34060/	gi|224118512	Plastid-specific 30S ribosomal
	718.6361	2870.4774	HGVVRAEEDAETIYASIDLVSSIIQR	22	------		6.78	*Populus trichocarpa*	protein 1
	775.0727	2322.1590	AEEDAETIYASIDLVSSIIQR	57	------				
	817.7643	2450.2540	AEEDAETIYASIDLVSSIIQRK	12	------				

**299**						ND			
**301**	674.3616	1346.6830	IGGAEDVFVGDIR	74	------	Predicted protein	27040/	gi|224090705	NAD-dependent
	827.5118	2479.4149	ESKSIVPAIQGIDSLIILTSAVPK	43	Deamidated (NQ)		5.68	*Populus trichocarpa*	epimerase/dehydratase
	872.4516	2614.2129	GRPEFYFEDGAFPEQVDWIGQK	31	------				
**305**	499.9529	1496.7736	HPGATVGAVEKEFR	30	------	Unknown	33398/	gi|118484329	---------
	608.8063	1215.5560	NGWFYSLSDK	34	------		6.97	*Populus trichocarpa*	
	753.4127	1504.7443	ALQEMGSGQDLLVK	20	Deamidated (NQ); Oxidation (M)				
	827.4498	1652.8410	GLGDADQVLAYFAVSK	57	------				
**308**	613.3087	1224.6139	SFFLGQVGNGAK	51	Deamidated (NQ)	Predicted protein	30624/	gi|224129290	3-hydroxyisobutyrate
	832.1159	2493.2686	SGLNPHDLLDILDLGGIANPMFR	19	Oxidation (M)		6.45	*Populus trichocarpa*	dehydrogenase
	845.4351	2533.2733	LALALGDENAVSMPVAAAANESFKK	22	Deamidated (NQ); Oxidation (M)				
**310**	444.5664	1330.6670	LAWHSAGTFDVK	10	------	Predicted protein	27302/	gi|224104631	Cytosolic ascorbate peroxidase
	550.3132	1098.5921	EGLLQLPSDK	40	------		5.53	*Populus trichocarpa*	1
	619.3355	1854.9938	ELLSGEKEGLLQLPSDK	27	------				
	624.3213	1869.9333	YSAELAHGANNGLDIAVR	22	------				
	688.3066	2061.8956	YAADEDAFFADYSEAHLK	4	------				
	922.4635	921.4444	LSELGFADA.-	36	------				
**313**	496.7762	991.5451	ALFSQVTAR	58	------	Predicted protein	27040/	gi|224090705	NAD-dependent
	535.6081	1603.8206	EKIGGAEDVFVGDIR	28	------		5.68	*Populus trichocarpa*	epimerase/dehydratase
	570.3088	1138.6135	ALFSQVTARF.-	28	------				
	599.3486	1196.6877	APSIVLVTGAGGR	35	------				
	620.3327	1857.9684	ELLVGKDDELLQTETR	61	------				
	642.0008	1923.0101	KAEQYLADSGVPYTILR	20	------				
	655.9877	1964.9480	AFDLASKPEGTGTPANDFK	40	------				
	674.3494	1346.6830	IGGAEDVFVGDIR	95	------				
	827.4714	2479.4149	ESKSIVPAIQGIDSLIILTSAVPK	10	Deamidated (NQ)				
	898.4791	1794.9152	AEQYLADSGVPYTILR	64	------				
	1021.5425	3061.6019	QIVLVGSMGGTNLNHPLNSLGNGNILVWK	18	Deamidated (NQ); Oxidation (M)				
**314**	496.7783	991.5451	ALFSQVTAR	39	------	Predicted protein	27040/	gi|224090705	NAD-dependent
	570.3132	1138.6135	ALFSQVTARF.-	53	------		5.68	*Populus trichocarpa*	epimerase/dehydratase
	620.3304	1857.9684	ELLVGKDDELLQTETR	39	------				
	642.0175	1923.0101	KAEQYLADSGVPYTILR	22	------				
	655.9901	1964.9480	AFDLASKPEGTGTPANDFK	22	------				
	674.3494	1346.6830	IGGAEDVFVGDIR	101	------				
	898.4595	1794.9152	AEQYLADSGVPYTILR	37	------				
	1240.7207	2479.4149	ESKSIVPAIQGIDSLIILTSAVPK	30	Deamidated (NQ)				
**315**	503.2827	1004.5655	FETAIGVLR	6	------	Unknown	27756/	gi|118484162	Putative ATP synthase
	952.9575	1903.9163	IQFTIEEETQDIPDAR	53	------		8.50	*Populus trichocarpa*	
**317**	480.7691	959.5440	ALIPDLYR	37	------	Predicted protein	26189/	gi|224131618	Carboxymethylenebutenolidase,
	587.3040	1172.6190	DIQASVNWLK	30	------		5.24	*Populus trichocarpa*	putative
	665.8007	1329.6089	DDTTFDAYVVGK	9	------				
	808.0716	2421.1489	APVQAHFGELDNFVGFSDVTAAK	42	2 Deamidated (NQ)				
**319**	521.3018	1560.8875	YTSITPLGDRVLVK	18	------	Predicted protein	27099/	gi|224141565	Groes chaperonin, putative
	521.9655	1562.8668	KALSVSPGNTVLYSK	23	------		7.77	*Populus trichocarpa*	
	587.8334	1173.6605	TAGGLLLTEATK	61	------				
	644.3473	1930.0258	VAEAEEKTAGGLLLTEATK	47	------				
	718.3985	1434.7718	ALSVSPGNTVLYSK	25	------				
**320**	429.9965	1715.8730	IHSVEEFDEALKTAK	54	------	Predicted protein	26271/	gi|224085954	Chloroplast drought-induced
	500.3197	998.5760	GELIGEILR	47	------		5.94	*Populus trichocarpa*	stress protein
**329**						ND			
**332**	412.2495	1233.6829	ISEYVAQLRR	32	------	Predicted protein	29441/	gi|224109256	Ferritin
	424.5671	1270.6193	FFKESSIEER	5	------		5.72	*Populus trichocarpa*	
	539.8208	1077.5818	ISEYVAQLR	60	------				
	558.5766	2230.1746	GANNRPLTGVVFEPFEEVKK	23	------				
	799.4397	2395.1795	DVQLTDFVESEFLAEQVDAIK	29	------				
**333**	545.9329	1634.7477	WWNEISDRESWK	12	------	Predicted protein	24603/	gi|224065729	Glutathione-s-transferase Phi
	553.2527	1104.4988	WWNEISDR	24	------		5.52	*Populus trichocarpa*	
	623.8482	1245.6717	VYGPPLSTAVSR	19	------				
	799.0685	2394.1856	IQPFGQVPAFQDESISLFESR	40	------				
	1055.8656	3164.5567	ASIDQWVEAEGQSFGPSSGALVFQLAFAPR	53	------				
**334**	457.7914	913.5848	VLVTLLEK	54	------	Predicted protein	24603/	gi|224065729	Glutathione-s-transferase theta
	518.2755	1034.5396	VLDIYEQR	53	------		5.52	*Populus trichocarpa*	
	547.0364	2184.1208	MNIPQDQGVIKQNEEKLGK	13	Oxidation (M)				
	623.8421	1245.6717	VYGPPLSTAVSR	71	------				
	752.9008	1503.7643	DVPFQIIPVDMSK	25	Oxidation (M)				
	799.0607	2394.1856	IQPFGQVPAFQDESISLFESR	64	------				
	800.7920	2399.3386	VLVTLLEKDVPFQIIPVDMSK	26	Oxidation (M)				
	943.2246	3768.8060	FLAGDEFTFADLSHLPNGDYLVNATDKGHLFTSR	43	Deamidated (NQ)				
	990.8212	2969.4083	FLAGDEFTFADLSHLPNGDYLVNATDK	10	------				
	1055.8802	3164.5567	ASIDQWVEAEGQSFGPSSGALVFQLAFAPR	81	------				

**346**	440.2406	878.4650	QLWFASK	31	------	Unknown	29600/ 9.10	gi|118489937 *Populus trichocarpa x Populus deltoides*	Light-harvesting complex I protein Lhca3
	516.2544	1030.5124	QYFLGFEK	37	------				
	646.3452	1290.6720	WLAYGEIINGR	53	------				
	889.7925	2666.3017	YLGGSGEPAYPGGPLFNPLGFGKDEK	21	------				
	1080.2567	3237.7624	YAMLGAVGAIAPEILGKAGLIPPETALPWFR	4	Oxidation (M)				
	1171.9029	3512.6512	QSLSYLDGSLPGDFGFDPLGLSDPEGTGGFIEPK	12	Deamidated (NQ)				
**361**	667.8582	1333.6878	LFDDALTIPSSR	48	------	Predicted protein	26186/ 6.92	gi|224120952 *Populus trichocarpa*	Heat shock protein, putative
	695.7237	2084.1340	KLAVDISPFGLLDPLSPMR	7	Oxidation (M)				
	786.3632	2356.1039	QMLDTMDRLFDDALTIPSSR	22	2 Oxidation (M)				
**363**	558.3439	1114.6386	NGVLFINIPK	35	Deamidated (NQ)	Predicted protein	26186/ 6.92	gi|224120952 Populus trichocarpa	Heat shock protein, putative
	667.8655	1333.6878	LFDDALTIPSSR	29	------				
**384**	472.5989	1414.7820	LEDLRIPPAYTK	49	------	Ribulose-1,5-bisphosphate carboxylase oxygenase	49852/ 6.57	gi|6513629 *Ascarina* sp. Qiu-M149	------------
	493.2931	984.5716	ALRLEDLR	39	------				
	511.2697	1020.5240	DTDILAAFR	64	------				
	614.2981	1839.8566	EYKLNYYTPEYETK	13	------				
	710.8381	1419.6558	LNYYTPEYETK	46	------				
	733.3852	1464.7474	TFQGPPHGIQVER	55	------				
	808.3967	2422.1692	LNYYTPEYETKDTDILAAFR	50	------				
**394**	489.5858	1465.7314	TFQGPPHGIQVER	17	Deamidated (NQ)	Ribulose-1,5-bisphosphate carboxylase, large subunit	51138/ 6.33	gi|493246 *Disporum sessile*	-------------
	511.2655	1020.5240	DTDILAAFR	48	------				
	710.8306	1419.6558	LNYYTPEYQTK	14	Deamidated (NQ)				
	808.3941	2422.1692	LNYYTPEYQTKDTDILAAFR	19	Deamidated (NQ)				
**471**						ND			
**487**	496.7887	991.5702	TLIKQDFK	38	------	Predicted protein	23366/ 6.82	gi|224098455 *Populus trichocarpa*	Thylakoid lumenal 15 kDa protein
	521.6422	1561.9192	TLIKQDFKTSILR	19	------				
	554.3154	1106.6084	QDFKTSILR	54	------				
	640.8233	1279.6157	GQDLTGKDFSGR	31	------				
	697.6685	2090.0029	GSNITGADFTDVPLREDQR	17	------				
	781.8928	1561.7737	GSNITGADFTDVPLR	67	------				
	1011.0175	2019.9973	ANLSNANLEGALATGNTSFR	20	------				
	1142.0745	2282.1067	LLGASFFDADLTGADLSDADLR	95	------				
**594**	439.7608	1755.0043	ALRLEDLRIPPAYTK	41	------	Ribulose-1,5-bisphosphate carboxylase/oxygenase large subunit	52703/ 5.91	gi|110227087 *Populus alba*	------------
	440.7246	879.4464	NHGIHFR	40	------				
	456.7350	911.4647	AMHAVIDR	55	------				
	482.2749	962.5549	TGVGFKAGVK	45	------				
	493.2889	984.5716	ALRLEDLR	34	------				
	512.2409	1022.4669	DDFVEKDR	21	------				
	530.2888	1058.5542	VALEACVQAR	43	------				
	539.7990	1077.5931	LGLSAKNYGR	85	------				
	559.7359	1117.4638	FEFEAMDTL.-	13	Oxidation (M)				
	568.8121	1135.5999	QKNHGIHFR	50	------				
	577.3373	576.3384	AIFAR	13	------				
	581.3060	1740.8867	NEGRDLAREGNEIIR	11	------				
	594.3378	1186.6571	DNGLLLHIHR	53	------				
	614.2958	1839.8566	EYKLNYYTPEYETK	19	------				
	616.0186	1844.9996	LEGERDITLGFVDLLR	58	------				
	623.3248	1244.6360	EGNEIIREASK	39	------				
	631.3664	1260.7078	DITLGFVDLLR	82	------				
	643.3411	1284.6786	DLAREGNEIIR	25	------				
	665.7015	1994.0361	DITLGFVDLLRDDFVEK	52	------				
	694.6856	2080.9960	MSGGDHIHSGTVVGKLEGER	25	Oxidation (M)				
	708.4009	1414.7820	LEDLRIPPAYTK	46	------				
	710.8306	1419.6558	LNYYTPEYETK	39	------				
	711.6156	2842.3701	EYKLNYYTPEYETKDTDILAAFR	55	------				
	729.3215	2184.9746	GGLDFTKDDENVNSQPFMR	47	Oxidation (M)				
	732.7050	2195.0786	AGVKEYKLNYYTPEYETK	57	------				
	733.3802	1464.7474	TFQGPPHGIQVER	78	------				
	744.8509	1487.6854	EIKFEFEAMDTL.-	23	Oxidation (M)				
	749.3630	1496.7042	MSGGDHIHSGTVVGK	54	Oxidation (M)				
	756.0756	2265.1641	DITLGFVDLLRDDFVEKDR	55	------				
	808.4021	2422.1692	LNYYTPEYETKDTDILAAFR	84	------				
	1021.5305	1020.5240	DTDILAAFR	58	------				
	1285.6567	3853.8647	VTPQPGVPPEEAGAAVAAESSTGTWTTVWTDGLTSLDR	51	------				

**598**	432.5701	1294.6994	DIVALSGGHTLGR	24	------	Predicted protein	27302/	gi|224104631	Ascorbate peroxidase
	545.3057	1632.8875	ALLSDPIFRPYVDK	63	------		5.53	*Populus trichocarpa*	
	550.2971	1098.5921	EGLLQLPSDK	52	------				
	619.3362	1854.9938	ELLSGEKEGLLQLPSDK	68	------				
	624.6502	1870.9173	YSAELAHGANNGLDIAVR	71	Deamidated (NQ)				
	625.3181	2497.2384	DVFGHMGLSDKDIVALSGGHTLGR	54	Oxidation (M)				
	666.3329	1330.6670	LAWHSAGTFDVK	50	------				
	688.3085	2061.8956	YAADEDAFFADYSEAHLK	49	------				
	764.0354	2289.0742	SGFEGPWTANPLIFDNSYFK	44	------				
	920.2055	3676.7725	ALLSDPIFRPYVDKYAADEDAFFADYSEAHLK	27	------				
	922.4533	921.4444	LSELGFADA.-	36	------				
**599**						ND			
**600**	432.2582	1293.7445	TFFLKLQEIR	29	------	Predicted protein	27827/	gi|224093896	------------
	489.2726	976.5342	SLDIRNFL.-	30	------		8.50	*Populus trichocarpa*	
**601**	452.2764	902.5437	TKLDISVK	42	------	Unknown	26829/	gi|118489858	Groes chaperonin, putative
	512.9562	1535.8559	KALSVSPGSTVLYSK	63	------		8.76	*Populus trichocarpa x*	
	530.3082	1587.9348	YTSIKPLGDRVLVK	14	------			*Populus deltoids*	
	575.3096	1148.6190	YTSIKPLGDR	44	------				
	587.8357	1173.6605	TAGGLLLTEATK	70	------				
	591.5781	2362.2492	EKPSIGTVIAVGPGPLDEEGNRK	11	------				
	635.3061	1902.9112	YAGNDFKGSDGANYIALK	55	------				
	649.0268	1944.0415	IAEAEEKTAGGLLLTEATK	17	------				
	704.8898	1407.7609	ALSVSPGSTVLYSK	64	------				
	745.7261	2234.1543	EKPSIGTVIAVGPGPLDEEGNR	10	------				
	922.4647	921.4477	ASDVMAILS.-	10	Oxidation (M)				
	936.4683	2806.3483	YAGNDFKGSDGANYIALKASDVMAILS.-	4	Oxidation (M)				
**602**	405.5752	1213.7183	IKIFAGDVVPR.	12	------	Unknown	35280/	gi|118488927	2-deoxyglucose-6-phosphate
	487.2799	972.5393	IFAGDVVPR	51	------		8.00	*Populus trichocarpa x*	phosphatase
	532.3523	1062.6913	LLPLRPGVAK	4	------			*Populus deltoides*	
	622.2838	1242.5517	ISFNDTFNER	69	Deamidated (NQ)				
	925.4880	1848.9509	ELGVTWDVDLYGELLK	30	------				
**603**	482.7896	963.5793	VPFLFTIK	53	------	Unknown	35133/	gi|118489901	Oxygen-evolving enhancer
	665.0317	1992.0739	ENIKNTASSTGKITLSVTK	4	Deamidated (NQ)		5.62	*Populus trichocarpa x*	protein 1
	766.7195	2297.1328	QLAASGKPENFSGEFLVPSYR	34	Deamidated (NQ)			*Populus deltoides*	
	781.8824	1561.7485	GGSTGYDNAVALPAGGR	54	------				
	873.4348	2617.3123	SKPETGEIIGVFESLQPSDTDLGAK	37	------				
	880.9449	1759.8741	DGIDYAAVTVQLPGGER	54	------				
	902.8228	2705.4429	DGIDYAAVTVQLPGGERVPFLFTIK	44	------				
	1162.5768	2323.1471	LTYTLDEIEGPFEVSPDGTIK	35	------				
**608**						ND			
**609**						ND			
**610**	482.7938	963.5793	VPFLFTIK	51	------	Unknown	35133/	gi|118489901	Oxygen-evolving enhancer
	610.8203	1219.6237	IQGIWYAQLE.-	19	------		5.62	*Populus trichocarpa x*	protein 1
	782.3883	1562.7325	GGSTGYDNAVALPAGGR	54	Deamidated (NQ)			*Populus deltoides*	
	873.4404	2617.3123	SKPETGEIIGVFESLQPSDTDLGAK	44	------				
	880.9449	1759.8741	DGIDYAAVTVQLPGGER	54	------				
**611**	425.7171	849.4232	GSSFLDPK	11	------	Putative protein	18483/	gi|190898996	Oxygen-evolving enhancer
	475.7813	949.5637	VPFLFTVK	46	------		5.17	*Populus tremula*	protein 1
	555.2803	1662.8035	NAPPEFQNTKLMTR	1	Deamidated (NQ); Oxidation (M)				
	626.8239	1251.6459	RLTYDEIQSK	30	------				
	767.3842	2299.1485	QLTASGKPESFSGEFLVPSYR	58	------				
	898.1560	2691.4272	DGIDYAAVTVQLPGGERVPFLFTVK	25	------				
**613**	668.3477	1334.6718	ATPDQVAEYTLK	46	------	Unknown	42900/	gi|118489355	Fructose-bisphosphate aldolase
	694.3669	1386.7103	LASIGLENTEANR	102	------		8.17	*Populus trichocarpa x*	
	727.4266	1452.8188	TVVSIPNGPSALAVK	45	Deamidated (NQ)			*Populus deltoides*	
	1016.5362	3046.5611	YAAISQDNGLVPIVEPEILLDGEHGIER	7	------				
	1096.2629	3285.7021	TLLVTVPGLGNYVSGAILFEETLYQSTTDGK	20	------				
	1138.9594	3413.7970	TLLVTVPGLGNYVSGAILFEETLYQSTTDGKK	11	------				
**614**	472.7783	943.5590	VLLTLEEK	51	------	Predicted protein	24340/	gi|224065178	DHAR class glutathione
	484.5977	1450.7932	ALPEDVIAGWRPK	16	------		4.93	*Populus trichocarpa*	transferase DHAR1
	486.2603	1455.7874	LYHLEIALGHYK	23	------				
	521.2932	1040.5866	VSAADLALGPK	79	------				
	531.6172	1591.8399	FVDLGNKPEWFLK	24	------				
	567.9874	1700.9501	ASVGSKIFSTFIGFLK	35	------				
	586.8338	1171.6641	IFSTFIGFLK	65	------				
	607.9878	1820.9594	VLLTLEEKNLPYDMK	21	Oxidation (M)				
	660.8537	1319.7125	FPDPPLAIPPEK	46	------				
	709.8737	1417.7242	NWSVPESLPYVK	34	------				
