# Supplementary material for: Effects of Heavy Metals and Arbuscular Mycorrhiza on the Leaf Proteome of a Selected Poplar Clone: A Time Course Analysis
Source: PLoS One. 2012 Jun 26;7(6):e38662. doi: 10.1371/journal.pone.0038662 (PMC3383689; doi:10.1371/journal.pone.0038662)
Supplement: Table S10 — BLAST results – first sampling (S1). Protein name, accession number and reference organism, BLAST results, percentage of homology, and percentage of identity. (PDF) [file pone.0038662.s011.pdf]

Table S10. BLAST results – first sampling (S1). Protein name, accession number and reference organism, BLAST results, percentage of homology, and percentage of identity.

Spot	Protein	AC number (gi NCBI) and reference organism	Blast results	% Homology	% Identity
**130**	Predicted protein	gi|224136806 *Populus trichocarpa*	Enolase [*Populus trichocarpa*]	100%	100%
**247**	Unknown	gi|118489355 *Populus trichocarpa x Populus deltoides*	Fructose-bisphosphate aldolase [*Ricinus communis*]	95%	90%
**283**	Unknown	gi|118488026 *Populus trichocarpa*	Thiamine biosynthetic enzyme [*Vitis vinifera*]	95%	91%
**304**	Predicted protein	gi|224072767 *Populus trichocarpa*	---------		
**314**	Predicted protein	gi|224090705 *Populus trichocarpa*	NAD-dependent epimerase/dehydratase [*Zea mays*]	92%	82%
**485**	Unknown	gi|219885633 *Zea mays*	Heat shock protein 70 [*Oryza sativa Japonica Group*]	97%	95%
**491**	Hypothetical protein SORBIDRAFT_03g039980	gi|242054991/ *Sorghum bicolor*	Laccase-8 [*Oryza sativa Japonica Group*]	86%	77%
**494**	Predicted protein	gi|224053971 *Populus trichocarpa*	Elongation factor Tu, chloroplastic [*Glycine max*]	91%	84%
