# Supplementary material for: Effects of Heavy Metals and Arbuscular Mycorrhiza on the Leaf Proteome of a Selected Poplar Clone: A Time Course Analysis
Source: PLoS One. 2012 Jun 26;7(6):e38662. doi: 10.1371/journal.pone.0038662 (PMC3383689; doi:10.1371/journal.pone.0038662)
Supplement: Table S11 — BLAST results – second sampling (S2). Protein name, accession number and reference organism, BLAST results, percentage of homology, and percentage of identity. (PDF) [file pone.0038662.s012.pdf]

Table S11. BLAST results – second sampling (S2). Protein name, accession number and reference organism, BLAST results, percentage of homology, and percentage of identity.

Spot	Protein	AC number (gi NCBI) and reference organism	Blast results	% Homology	% Identity
**118**	Unknown	gi|118487547 *Populus trichocarpa*	RuBisCO activase (RCA) [*Populus trichocarpa*]	100%	100%
**119**	Unknown	gi|118487547 *Populus trichocarpa*	RuBisCO activase (RCA) [*Populus trichocarpa*]	100%	100%
**122**	Predicted protein	gi|224109060 *Populus trichocarpa*	phosphoglycerate kinase [*Populus trichocarpa*]	98%	96%
**134**	Unknown	gi|118489408 *Populus trichocarpa x Populus deltoides*	RuBisCO activase (RCA) [*Populus trichocarpa*]	99%	98%
**135**	Unknown	gi|118486739 *Populus trichocarpa*	RuBisCO activase (RCA) [*Populus trichocarpa*]	100%	99%
**137**	Unknown	gi|118487547 *Populus trichocarpa*	RuBisCO activase (RCA) [*Populus trichocarpa*]	100%	100%
**142**	Unnamed protein product	gi|157345989 *Vitis vinifera*	Ribulose bisphosphate carboxylase/oxygenase activase 1, chloroplast precursor, putative [*Ricinus communis*]	94%	87%
**146**	Predicted protein	gi|224109060 *Populus trichocarpa*	phosphoglycerate kinase [*Populus trichocarpa*]	98%	96%
**149**	Predicted protein	gi|224079530 *Populus trichocarpa*	glutamine synthetase [*Populus trichocarpa*]	100%	100%
**150**	Predicted protein	gi|224145917 *Populus trichocarpa*	Uroporphyrinogen decarboxylase [*Populus trichocarpa*]	100%	100%
**152**	Predicted protein	gi|224071429 *Populus trichocarpa*	Phosphoribulokinase [*Populus trichocarpa*]	100%	100%
**155**	Predicted protein	gi|224071429 *Populus trichocarpa*	phosphoribulokinase [*Populus trichocarpa*]	99%	95%

**161**	Unknown	gi|118482960 *Populus trichocarpa*	Protein disulfide isomerase, putative [*Ricinus communis*]	94%	85%
**162**	Predicted protein	gi|224102193 *Populus trichocarpa*	Malate dehydrogenase [*Populus trichocarpa*]	100%	100%
**164**	Predicted protein	gi|224069096 *Populus trichocarpa*	Aldo/keto reductase AKR [*Manihot esculenta*]	91%	84%
**172**	Hypothetical protein	gi|147835353 *Vitis vinifera*	---------		
**174**	Unknown	gi|118489355 *Populus trichocarpa x Populus deltoides*	Fructose-bisphosphate aldolase, putative [*Ricinus communis*]	95%	90%
**181**	Unknown	gi|118487575 *Populus trichocarpa*	Fructose-bisphosphate aldolase, putative [*Ricinus communis*]	95%	91%
**245**	Predicted protein	gi|224090705 *Populus trichocarpa*	NAD-dependent epimerase/dehydratase [*Zea mays*]	92%	82%
**246**	Predicted protein	gi|224104631 *Populus trichocarpa*	Cytosolic ascorbate peroxidase 1 [*Gossypium hirsutum*]	93%	88%
**253**	Predicted protein	gi|224141565 *Populus trichocarpa*	Groes chaperonin, putative [*Ricinus communis*]	91%	84%
**255**	Predicted protein	gi|224130670 *Populus trichocarpa*	Ribose-5-phosphate isomerase, putative [*Ricinus communis*]	93%	85%
**269**	Predicted protein	gi|224117556 *Populus trichocarpa*	Tau class glutathione transferase GSTU30 [*Populus trichocarpa*]	100%	100%
**272**	Hypothetical protein POPTRDRAFT_551203	gi|224062595 *Populus trichocarpa*	Photosystem II reaction center psbP protein [*Populus trichocarpa*]	100%	100%
**275**	Predicted protein	gi|224068558 *Populus trichocarpa*	ATP-dependent Clp protease proteolytic subunit, putative [*Ricinus communis*]	91%	87%

**291**	Hypothetical protein POPTRDRAFT_818640	gi|224085421 *Populus trichocarpa*	Probable oxygen-evolving enhancer protein 2 [*Vitis vinifera*]	90%	80%
**403**	Predicted protein	gi|224074257 *Populus trichocarpa*	Ferredoxin--NADP reductase, putative [*Ricinus communis*]	96%	91%
**409**	Putative protein	gi|190898996 *Populus tremula*	Oxygen-evolving enhancer protein 1, chloroplast precursor, putative [*Ricinus communis*]	97%	92%
**411**	Unknown	gi|118489901 *Populus trichocarpa x Populus deltoides*	Photosystem II oxygen-evolving complex 33 KDa subunit [*Populus trichocarpa*]	99%	98%
**415**	Predicted protein	gi|224146717 *Populus trichocarpa*	Protein THYLAKOID FORMATION1, chloroplast precursor, putative [*Ricinus communis*]	89%	77%
**419**	Predicted protein	gi|224124440 *Populus trichocarpa*	manganese superoxide dismutase [*Populus trichocarpa*]	93%	89%
**420**	Unknown	gi|118489105 *Populus trichocarpa x Populus deltoides*	Ribulose bisphosphate carboxylase/oxygenase activase 1, chloroplast precursor, putative [*Ricinus communis*]	94%	88%
**423**	Putative protein	gi|190898996 *Populus tremula*	Oxygen-evolving enhancer protein 1, chloroplast precursor, putative [*Ricinus communis*]	97%	92%
