# Supplementary material for: Effects of Heavy Metals and Arbuscular Mycorrhiza on the Leaf Proteome of a Selected Poplar Clone: A Time Course Analysis
Source: PLoS One. 2012 Jun 26;7(6):e38662. doi: 10.1371/journal.pone.0038662 (PMC3383689; doi:10.1371/journal.pone.0038662)
Supplement: Table S12 — BLAST results – third sampling (S3). Protein name, accession number and reference organism, BLAST results, percentage of homology, and percentage of identity. (PDF) [file pone.0038662.s013.pdf]

Table S12. BLAST results – third sampling (S3). Protein name, accession number and reference organism, BLAST results, percentage of homology, and percentage of identity.

Spot	Protein	AC number (gi NCBI) and reference organism	Blast results	% Homology	% Identity
**105**	Predicted protein	gi|224071575 *Populus trichocarpa*	heat shock protein 70 (HSP70)-interacting protein, putative [*Ricinus communis*]	90%	82%
**118**	Predicted protein	gi|224104681 *Populus trichocarpa*	putative rubisco subunit binding-protein alpha subunit (Chaperonin) [*Trifolium pratense*]	93%	87%
**171**	Predicted protein	gi|224138316 *Populus trichocarpa*	phosphoribulose kinase, putative [*Ricinus communis*]	97%	93%
**176**	Unknown	gi|118489105 *Populus trichocarpa x Populus deltoides*	Ribulose bisphosphate carboxylase/oxygenase activase 1, chloroplast precursor, putative [*Ricinus communis*]	94%	88%
**197**	Predicted protein	gi|224074859 *Populus trichocarpa*	EF-Tu protein [*Glycine max*]	96%	91%
**199**	Unknown	gi|118489408 *Populus trichocarpa x Populus deltoides*	RuBisCO activase (RCA) [*Populus trichocarpa*]	99%	98%
**209**	Unknown	gi|118489105 *Populus trichocarpa x Populus deltoides*	Ribulose bisphosphate carboxylase/oxygenase activase 1, chloroplast precursor, putative [*Ricinus communis*]	94%	88%
**212**	Unknown	gi|118487547 *Populus trichocarpa*	Ribulose bisphosphate carboxylase/oxygenase activase - RuBisCO activase (RCA) [*Populus trichocarpa*]	100%	100%
**215**	Predicted protein	gi|224112589 *Populus trichocarpa*	Sedoheptulose-1,7-bisphosphatase, chloroplast, putative [*Ricinus communis*]	93%	90%

**216**	Predicted protein	gi|224109060 *Populus trichocarpa*	Phosphoglycerate kinase [*Populus trichocarpa*]	100%	100%
**223**	Predicted protein	gi|224138316 *Populus trichocarpa*	Phosphoribulose kinase, putative [*Ricinus communis*]	97%	93%
**227**	Predicted protein	gi|224071429 *Populus trichocarpa*	Phosphoribulose kinase, putative [*Ricinus communis*]	97%	92%
**236**	Unknown	gi|118488941 *Populus trichocarpa x Populus deltoides*	Alcohol dehydrogenase, putative [*Ricinus communis*]	90%	83%
**241**	Hypothetical protein	gi|225446767 *Vitis vinifera*	Aldo/keto reductase, putative [*Ricinus communis*]	89%	80%
**244**	Predicted protein	gi|224053535 *Populus trichocarpa*	Pyruvate dehydrogenase (acetyl-transferring) [*Populus trichocarpa*]	100%	100%
**247**	Unknown	gi|118488941 *Populus trichocarpa x Populus deltoides*	Alcohol dehydrogenase, putative [*Ricinus communis*]	90%	83%
**261**	Predicted protein	gi|224073126 *Populus trichocarpa*	----------		
**286**	Predicted protein	gi|224110036 *Populus trichocarpa*	--------		
**290**	Predicted protein	gi|224074257 *Populus trichocarpa*	Ferredoxin--NADP reductase, putative [*Ricinus communis*]	96%	91%
**293**	Predicted protein	gi|224093744 *Populus trichocarpa*	2-deoxyglucose-6-phosphate phosphatase, putative [*Ricinus communis*]	94%	91%
**295**	Predicted protein	gi|224118512 *Populus trichocarpa*	Plastid-specific 30S ribosomal protein 1, chloroplast precursor, putative [*Ricinus communis*]	80%	70%
**301**	Predicted protein	gi|224090705 *Populus trichocarpa*	NAD-dependent epimerase/dehydratase [*Zea mays*]	92%	82%
**305**	Unknown	gi|118484329 *Populus trichocarpa*	-------		

**308**	Predicted protein	gi|224129290 *Populus trichocarpa*	3-hydroxyisobutyrate dehydrogenase, putative [*Ricinus communis*]	94%	85%
**310**	Predicted protein	gi|224104631 *Populus trichocarpa*	Cytosolic ascorbate peroxidase 1 [*Gossypium hirsutum*]	93%	88%
**313**	Predicted protein	gi|224090705 *Populus trichocarpa*	NAD-dependent epimerase/dehydratase [*Zea mays*]	92%	82%
**314**	Predicted protein	gi|224090705 *Populus trichocarpa*	NAD-dependent epimerase/dehydratase [*Zea mays*]	92%	82%
**315**	Unknown	gi|118484162 *Populus trichocarpa*	ProbableATP synthase subunit, mitochondrial [*Vitis vinifera*]	92%	82%
**317**	Predicted protein	gi|224131618 *Populus trichocarpa*	Carboxymethylenebutenolidase, putative [*Ricinus communis*]	92%	86%
**319**	Predicted protein	gi|224141565 *Populus trichocarpa*	Groes chaperonin, putative [*Ricinus communis*]	91%	84%
**320**	Predicted protein	gi|224085954 *Populus trichocarpa*	Chloroplast drought-induced stress protein, putative [*Arabidopsis thaliana*]	90%	83%
**332**	Predicted protein	gi|224109256 *Populus trichocarpa*	Chloroplast ferritin 2 precursor [*Jatropha curcas*]	89%	84%
**333**	Predicted protein	gi|224065729 *Populus trichocarpa*	Phi class glutathione transferase GSTF2 [*Populus trichocarpa*]	100%	100%
**334**	Predicted protein	gi|224065729 *Populus trichocarpa*	Glutathione-s-transferase theta, gst, putative [*Ricinus communis*]	94%	83%
**346**	Unknown	gi|118489937 *Populus trichocarpa x Populus deltoides*	Light-harvesting complex I protein Lhca3 [*Populus trichocarpa*]	99%	99%
**361**	Predicted protein	gi|224120952 *Populus trichocarpa*	Heat shock protein, putative [*Ricinus communis*]	83%	73%

**363**	Predicted protein	gi|224120952 *Populus trichocarpa*	Heat shock protein, putative [*Ricinus communis*]	83%	73%
**487**	Predicted protein	gi|224098455 *Populus trichocarpa*	Thylakoid lumenal 15 kDa protein, chloroplast [*Arabidopsis lyrata subsp. lyrata*]	91%	84%
**598**	Predicted protein	gi|224104631 *Populus trichocarpa*	Cytosolic ascorbate peroxidase 1 [*Gossypium hirsutum*]	93%	88%
**600**	Predicted protein	gi|224093896 *Populus trichocarpa*	---------		
**601**	Unknown	gi|118489858 *Populus trichocarpa x Populus deltoids*	Groes chaperonin, putative [*Ricinus communis*]	92%	84%
**602**	Unknown	gi|118488927 *Populus trichocarpa x Populus deltoides*	2-deoxyglucose-6-phosphate phosphatase [*Ricinus communis*]	88%	83%
**603**	Unknown	gi|118489901 *Populus trichocarpa x Populus deltoides*	Photosystem II oxygen-evolving complex 33 KDa subunit [*Populus trichocarpa*]	99%	98%
**610**	Unknown	gi|118489901 *Populus trichocarpa x Populus deltoides*	Photosystem II oxygen-evolving complex 33 KDa subunit [*Populus trichocarpa*]	99%	98%
**611**	Putative protein	gi|190898996 *Populus tremula*	Oxygen-evolving enhancer protein 1, chloroplast precursor, putative [*Ricinus communis*]	97%	92%
**613**	Unknown	gi|118489355 *Populus trichocarpa x Populus deltoides*	Fructose-bisphosphate aldolase, putative [*Ricinus communis*]	95%	90%
**614**	Predicted protein	gi|224065178 *Populus trichocarpa*	DHAR class glutathione transferase DHAR1 [*Populus trichocarpa*]	100%	99%
