# Supplementary material for: Effects of Heavy Metals and Arbuscular Mycorrhiza on the Leaf Proteome of a Selected Poplar Clone: A Time Course Analysis
Source: PLoS One. 2012 Jun 26;7(6):e38662. doi: 10.1371/journal.pone.0038662 (PMC3383689; doi:10.1371/journal.pone.0038662)
Supplement: Table S13 — Two-way ANOVA – first sampling (S1). List of the spots showing significant P values for the two-way ANOVA for the factors Fungus, Metal or Fungus×Metal. Empty cells in the table correspond to non-significant P-values. (PDF) [file pone.0038662.s014.pdf]

Table S13. Two-way ANOVA – first sampling (S1). List of the spots showing significant P values for the two-way ANOVA for the factors Fungus, Metal or Fungus x Metal. Empty cells in the table correspond to non-significant P-values.

Spot	Fungus	Metal	Fungus x Metal
104	0.0108	0.0132	
112	0.0129		
124	0.0012		
130	0.0057		
153	0.0015		
154	0.0014		
165	0.0168		
230	0.0094	0.0464	
247	0.0005		
283	0.0238	0.0201	0.0003
304	0.0037	0.0278	
314	0.0007		
366	0.0004	0.0068	0.0294
397	0.0010	0.0059	
445	0.0134	0.0127	
470	< 0.0001		0.0390
471	< 0.0001		
484	0.0059		
485	0.0024		
489	0.0486		
491	0.0085		
494	< 0.0001		
