# Supplementary material for: Effects of Heavy Metals and Arbuscular Mycorrhiza on the Leaf Proteome of a Selected Poplar Clone: A Time Course Analysis
Source: PLoS One. 2012 Jun 26;7(6):e38662. doi: 10.1371/journal.pone.0038662 (PMC3383689; doi:10.1371/journal.pone.0038662)
Supplement: Table S14 — Two-way ANOVA – second sampling (S2). List of the spots showing significant P values for the two-way ANOVA for the factors Fungus, Metal or Fungus×Metal. Empty cells in the table correspond to non-significant P-values. (PDF) [file pone.0038662.s015.pdf]

Table S14. Two-way ANOVA – second sampling (S2). List of the spots showing significant P values for the two-way ANOVA for the factors Fungus, Metal or Fungus x Metal. Empty cells in the table correspond to non-significant P-values.

Spot	Fungus	Metal	Fungus x Metal
57		0.0014	
118	0.0007		
119		0.0020	
122	0.0086	0.0020	
132	0.0002	0.0131	
134	0.0260	0.0032	
135		0.0180	
137	0.0308	< 0.0001	
142		0.0006	
146	0.0246	0.0042	
148		0.0004	0.0193
149		0.0078	0.0108
150		0.0058	
152	0.0089	0.0001	
155	0.0175	0.0025	
161	0.0013	< 0.0001	
162	0.0097	0.0003	
163	0.0243	0.0051	
164	0.0383	0.0002	
165	0.0251	0.0109	
166	0.0271	0.0022	0.0141
171	0.0008	< 0.0001	
172		0.0004	
174	0.0237	0.0033	
181		0.0027	
193		0.0137	
202		0.0002	< 0.0001
214	< 0.0001	0.0249	< 0.0001
241	0.0499	0.0084	
245		0.0035	
246		< 0.0001	
253		0.0385	0.0027
254		0.0014	
255	0.0005	< 0.0001	0.0106
269	0.0034		
272		0.0008	< 0.0001
275	0.0050	0.0003	
291		0.0002	0.0005
294		0.0005	
303	0.0041	0.0016	

312		< 0.0001	
402		0.0005	0.0001
403		0.0013	0.0081
409		< 0.0001	0.0076
410		0.0252	0.0092
411		0.0001	0.0170
414		0.0012	
415		0.0034	
419		0.0005	
420		0.0005	
421		0.0027	
423			0.0121
