# Supplementary material for: Effects of Heavy Metals and Arbuscular Mycorrhiza on the Leaf Proteome of a Selected Poplar Clone: A Time Course Analysis
Source: PLoS One. 2012 Jun 26;7(6):e38662. doi: 10.1371/journal.pone.0038662 (PMC3383689; doi:10.1371/journal.pone.0038662)
Supplement: Table S15 — Two-way ANOVA – third sampling (S3). List of the spots showing significant P values for the two-way ANOVA for the factors Fungus, Metal or Fungus×Metal. Empty cells in the table correspond to non-significant P-values. (PDF) [file pone.0038662.s016.pdf]

Table S15. Two-way ANOVA – third sampling (S3). List of the spots showing significant P values for the two-way ANOVA for the factors Fungus, Metal or Fungus x Metal. Empty cells in the table correspond to non-significant P-values.

Spot	Fungus	Metal	Fungus x Metal
85	0.0031	0.0085	0.0489
105		< 0.0001	0.0346
118	0.0001	0.0380	
132	0.0053	0.0281	
171	0.0010	0.0138	0.0068
176	0.0019	0.0051	
178	< 0.0001	0.0084	0.0024
197		0.0222	
199	< 0.0001	0.0005	
200	< 0.0001	0.0198	0.0026
204	< 0.0001	0.0009	
209	< 0.0001	0.0334	0.0002
212	0.0003	0.0217	0.0112
215	0.0045	0.0441	0.0483
216	< 0.0001	0.0034	< 0.0001
223	< 0.0001	0.0178	
227	< 0.0001		
236	0.0002	0.0014	0.0015
238	0.0002	< 0.0001	
241	0.0019	< 0.0001	0.0157
244	< 0.0001		
247	0.0210	0.0008	0.0023
261	< 0.0001	0.0012	
270	< 0.0001	0.0228	
277		0.0007	
279	< 0.0001	< 0.0001	
286	0.0164	0.0306	
289	0.0013	< 0.0001	
290	0.0001	0.0019	0.0008
293	< 0.0001	0.0010	0.0480
295	0.0357	< 0.0001	
299	0.0068	0.0017	0.0112
301	< 0.0001	< 0.0001	
305	0.0080	0.0047	
308	< 0.0001	0.0001	
310	0.0043	< 0.0001	
313	< 0.0001	< 0.0001	0.0002
314	0.0005	< 0.0001	0.0090
315	0.0020	0.0007	
317	0.0280	0.0007	0.0073
319	0.0120	0.0026	0.0151

320	< 0.0001	0.0004	
329	< 0.0001	< 0.0001	< 0.0001
332	0.0054	0.0088	
333	0.0032	0.0182	
334	< 0.0001	< 0.0001	0.0030
346	0.0063	0.0132	
361	< 0.0001	< 0.0001	0.0034
363	0.0002		
384	< 0.0001		
394	0.0013	< 0.0001	
487	0.0035	0.0017	
594		0.0026	
598	< 0.0001	0.0008	0.0075
599	0.0162		
600	< 0.0001	< 0.0001	
601	< 0.0001	0.0034	
602	0.0091	0.0129	
603	< 0.0001	< 0.0001	
608	0.0446	< 0.0001	0.0049
609	0.0200	< 0.0001	
610		0.0125	0.0118
611	< 0.0001	< 0.0001	0.0020
613	< 0.0001	0.0442	0.0308
614	0.0155	0.0028	0.0266
